# UHRF1 suppresses retrotransposons and cooperates with PRMT5 and PIWI proteins in male germ cells

**DOI:** 10.1038/s41467-019-12455-4

**Published:** 2019-10-17

**Authors:** Juan Dong, Xiaoli Wang, Congcong Cao, Yujiao Wen, Akihiko Sakashita, Si Chen, Jin Zhang, Yue Zhang, Liquan Zhou, Mengcheng Luo, Mingxi Liu, Aihua Liao, Satoshi H. Namekawa, Shuiqiao Yuan

**Affiliations:** 1 Institute of Reproductive Health, Tongji Medical College, Huazhong University of Science and Technology, Wuhan, 430030 China; 2 Division of Reproductive Sciences, Cincinnati Children’s Hospital Medical Center, Cincinnati, OH 45229 USA; 3 College of Animal Science and Technology, Northwest A&F University, Yangling, 712100 China; 4 State Key Laboratory of Reproductive Medicine, Nanjing Medical University, Nanjing, 210029 China; 5 Department of Tissue and Embryology, School of Basic Medical Sciences, Wuhan University, Wuhan, 430071 China; 6 Hubei Provincial Key Laboratory of Developmentally Originated Disease, Wuhan, 430071 China; 7 Shenzhen Huazhong University of Science and Technology Research Institute, Shenzhen, 518057 China

**Keywords:** Development, DNA methylation, Infertility, Infertility

## Abstract

DNA methylation, repressive histone marks, and PIWI-interacting RNA (piRNA) are essential for the control of retrotransposon silencing in the mammalian germline. However, it remains unknown how these repressive epigenetic pathways crosstalk to ensure retrotransposon silencing in the male germline. Here, we show that UHRF1 is responsible for retrotransposon silencing and cooperates with repressive epigenetic pathways in male germ cells. Conditional loss of UHRF1 in postnatal germ cells causes DNA hypomethylation, upregulation of retrotransposons, the activation of a DNA damage response, and switches in the global chromatin status, leading to complete male sterility. Furthermore, we show that UHRF1 interacts with PRMT5, an arginine methyltransferase, to regulate the repressive histone arginine modifications (H4R3me2s and H3R2me2s), and cooperates with the PIWI pathway during spermatogenesis. Collectively, UHRF1 regulates retrotransposon silencing in male germ cells and provides a molecular link between DNA methylation, histone modification, and the PIWI pathway in the germline.

## Abstract (web summary)

Retrotransposons are silenced by an epigenetic mechanism and piRNA pathway in the mammalian germline. Here, the authors report that UHRF1 suppresses retrotransposons by interacting with PRMT5 and PIWI proteins during spermatogenesis.

## Introduction

Germline genome integrity is fundamental for living organisms, and transposable element (TE) silencing is essential for protecting the mammalian germline. In mammalian evolution, TEs are responsible for genetic diversification^1^. Nevertheless, in the short term, TE-induced events can threaten genomic integrity and contribute to oncogenesis, developmental pathologies, and infertility^2–4^. To protect the germline from TE-induced events, several repressive pathways are responsible for silencing retrotransposons^5,6^. DNA methylation plays a key role in silencing retrotransposons and functions together with histone modifications^7–12^. Mutations in DNA methyltransferase genes (such as *Dnmt1, Dnmt3a/3l*) result in retrotransposon reactivation and lead to mouse embryonic lethality and/or male sterility^13–15^. However, in mouse embryonic stem cells (ESCs), repressive histone marks (H3K9 methylation) are the main mechanisms in repressing retrotransposon transcription with some assistance from DNA methylation^16–18^. In addition, PIWI-interacting RNAs (piRNAs) play a crucial role in silencing retrotransposons in the germline and maintaining genome integrity^19^. Disruption of the piRNA pathway and loss-of-function of PIWI family proteins (MIWI, MILI, and MIWI2) often result in transposon upregulation, spermatogenic arrest, and male sterility^20–22^. Recent studies demonstrated that these repressive epigenetic pathways are interrelated in germ cell development^23–25^, yet an important question remains as to how they interplay to ensure retrotransposon silencing.

UHRF1 (ubiquitin-like, containing PHD and RING finger domains 1), also known as ICBP90 in humans and NP95 in mice, emerges as a key regulator between DNA methylation maintenance and histone modifications. UHRF1 binds to hemimethylated DNA and recruits DNMT1 to maintain DNA methylation during DNA replication^26–30^ and also binds to H3K9me3 in mitosis^31–33^. Thus, UHRF1 likely serves as a molecular link between DNA methylation and H3K9me3 in mitosis. In addition, UHRF1 interacts with several histone modulators: DNMT3a/b (the de novo methyltransferases)^34^, HDAC1 (the histone deacetylase)^35^, TIP60 (the histone acetyltransferase)^36^, and PRMT5 (the type II arginine methyltransferase)^37^. PRMT5 mediates repressive histone modifications, symmetrically dimethylated H2A and H4 (H2A/H4R3me2s)^38^ and regulates retrotransposon silencing in early primordial germ cells (PGCs)^25^.

Here, we show that UHRF1 is essential for spermatogenesis and underlies the crosstalk of repressive epigenetic pathways to suppress retrotransposons in meiotic prophase I. Conditional deletion of *Uhrf1* in differentiating spermatogonia leads to meiotic defects and sterility, presumably due to a combination of effects from the loss of DNA methylation, the decrease of histone arginine methylation, and aberrance of piRNA pathways. We discovered that UHRF1 is required for suppression of retrotransposons and identified a critical role for UHRF1 in cooperation with UHRF1, PRMT5, and PIWI proteins in male meiosis. These results unveil UHRF1 as a molecular link among DNA methylation, repressive histone marks and the PIWI pathway to safeguard germ cell genomic integrity during spermatogenesis.

## Results

### UHRF1 displays a dynamic nuclear-cytoplasmic expression

Multi-alignment and phylogenetic analyses of UHRF1 revealed that *Uhrf1* encodes a highly conserved protein expressed in multiple vertebrate species, including mice, humans, rats, bovines, and zebra fish, etc. (Supplementary Fig. 1a, b). In this study, we found that UHRF1 is expressed in mouse reproductive organs and both mRNA and protein of *Uhrf1* are continually expressed in postnatal day 0 (P0) testes to adult testes (Supplementary Fig. 1c–f). Immunofluorescence staining of UHRF1 in adult testes showed a high level of UHRF1 in spermatogonia and spermatocytes but not in Sertoli cells (Supplementary Fig. 1g). These results indicate that *Uhrf1* is continually expressed in male germ cells postnatally.

**Fig. 1:**
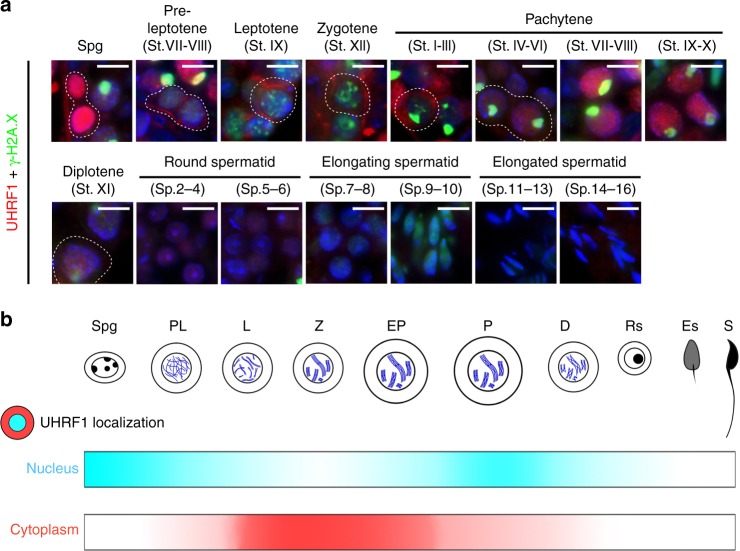
UHRF1 displays a dynamic expression profile during adult spermatogenesis. **a** Double immunostaining with UHRF1 and γ-H2A.X on WT (wild-type) germ cells from adult testis sections are shown. Scale bar = 10 μm. **b** A schematic summary of the dynamic localizations of UHRF1 in adult testis during spermatogenesis. Note: the localization drawing based on the fluorescent signal analyses from five independent experiments. Spg, Spermatogonia; PL, Pre-leptotene; L, Leptotene; Z, Zygotene; EP, early pachytene; P, Pachytene; D, Diplotene; Rs, Round spermatids; Es, Elongating spermatids; S, Spermatozoa

We next determined the subcellular localization of UHRF1 during spermatogenesis by co-staining UHRF1 with γ-H2AX (a marker of meiotic DNA damage response) and/or SYCP3 (a marker of meiotic chromosome axes). We observed the presence of UHRF1 throughout most stages of germ cell development and spermatogenesis, including in mitotic spermatogonia, meiotic spermatocytes (pre-leptotene to diplotene) and early round spermatids (Fig. 1a, Supplementary Fig. 2a). Interestingly, UHRF1 was abundant in the nuclei of neonatal pro-spermatonia at P0, spermatogonia, late pachytene spermatocytes and early round spermatids (steps 1–6); by comparison, UHRF1 was strongly expressed in the cytoplasm of fetal prospermatogonia at E15.5, pre-leptotene, leptotene, zygotene and early pachytene spermatocytes (Fig. 1a, b, Supplementary Fig. 2b). This dynamic of nuclear-cytoplasmic translocation of UHRF1 was also observed during the first wave of spermatogenesis (Supplementary Fig. 2c). Nuclear localization of UHRF1 during meiotic prophase was confirmed by immunostaining of chromosome spreads (Supplementary Fig. 2d). Interestingly, a recent study reported the cytoplasmic and nuclear localization of UHRF1 in mouse oocytes^39^. Therefore, cytoplasmic localization of UHRF1 is a common feature both in the male and female germline.

### UHRF1 is essential for spermatogenesis and male fertility

**Fig. 2:**
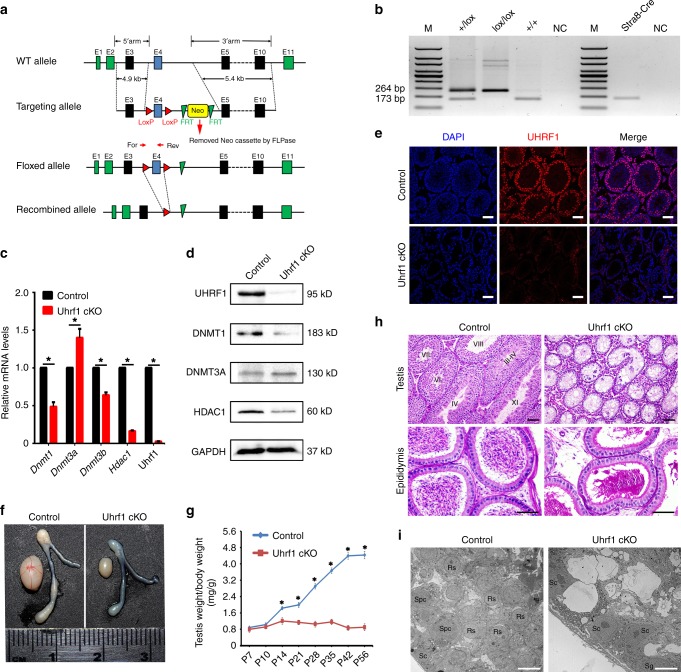
Conditional inactivation of *Uhrf1* in postnatal male germ cells results in spermatogenic arrest and male infertility in mice. **a** Schematic representation of the targeting strategy for generating a floxed *Uhrf1* allele through homologous recombination in the murine embryonic stem cells. Exons 4 will be deleted after *Cre*-mediated recombination. Position of the forward (For) and reverse (Rev) primers used for genotyping are shown. **b** Representative PCR genotyping results showing the floxed (lox) and the WT ( + ) alleles can be detected at 173 and 264 bp bands, respectively. **c** RT-qPCR analyses showing *Uhrf1* mRNA level was nearly undetectable and *Dnmt1*, *Dnmt3b*, and *Hdac1* mRNAs levels were also dramatic downregulated in *Uhrf1* cKO adult testes, whereas the expression level of *Dnmt3a* was upregulated. Data are presented as mean ± SEM, *n* = 3. *P < 0.05 by Student’s *t-*test. Source data are provided as a source data file. **d** Western blot of UHRF1, DNMT1, DNMT3A, and HDAC1 expression in adult control and *Uhrf1* cKO testes. GAPDH served as a loading control. **e** Representative immunofluorescent images showing UHRF1 was undetectable in adult *Uhrf1* cKO testis. Scale bar = 50 μm. **f** Gross morphology of the testis and the epididymis from control and *Uhrf1* cKO mice at postnatal day 56 (P56). **g** Testes growth curve shows the *Uhrf1* cKO testes were significantly decreased from P14. Data are presented as mean ± SEM, *n* = 2–5. Source data are provided as a source data file. **h** Periodic acid-Schiff (PAS) staining showing the histology of testis and epididymis sections from control and *Uhrf1* cKO mice at P60. Upper panels indicate testicular histology of control and *Uhrf1* cKO mice. Large vacuoles were seen in most seminiferous tubules of the *Uhrf1* cKO testes. Lower panels showing cauda epididymis was completely lacking spermatozoa at P60 of *Uhrf1* cKO mice. Scale bar = 50 μm. **i** Transmission electron microscopy images of control and *Uhrf1* cKO testis ultra-sections at P60. *Uhrf1* cKO testis sections showing massive degenerated germ cells, and very few normal spermatocytes were seen, whereas control testis sections displaying full germ cells development. Sc, Sertoli cells; Sg, Spermatogonia; Spc, Spermatocytes; Rs, Round spermatids. Scale bar = 10 μm

To elucidate the physiological role of *Uhrf1* in spermatogenesis, we generated germline specific knockout mice by using *Stra8-Cre* transgenic mice in which Cre is expressed in differentiating spermatogonia^40^ to delete exon 4 of *Uhrf1* gene (*Stra8-Cre; Uhrf1*^*flox/Del*^, herein called *Uhrf1* cKO) (Fig. 2a, b). Both mRNA and protein levels of UHRF1 in *Uhrf1* cKO adult testes were significantly decreased compared with that of WT controls (Fig. 2c–e), indicating that *Uhrf1* was inactivated specifically in testes with high efficiency. Further, co-staining of UHRF1 with DDX4 (a germ cell marker) in *Uhrf1* cKO and littermate control (*Stra8-Cre*; *Uhrf1*^*+/flox*^, herein called “control”) testes at P7 and P10 confirmed high recombination efficiency (more than 90%) from P7 onward (Supplementary Fig. 3). While *Uhrf1* cKO mice were viable and appeared to be grossly normal, they displayed complete sterility after a 5-month-period fecundity test. Consistent with this infertile phenotype, testis size from *Uhrf1* cKO mice was significantly smaller than their controls by ~30% (Fig. 2f). The testis weight of *Uhrf1* cKO mice were much reduced at various ages starting from P14 to P56 compared with controls (Fig. 2g). Histological analyses showed that adult *Uhrf1* cKO mice had abnormal seminiferous tubules that were severely atrophic and contained very few germ cells but many vacuoles (Fig. 2h). Further immunostainings revealed that the majority of germ cells were depleted, and no haploid spermatids were observed in *Uhrf1* cKO testes (Supplementary Fig. 4a, b). Transmission electron microscopy (TEM) analysis also revealed that most germ cells in *Uhrf1* cKO testis sections were degenerated in *Uhrf1* cKO testis sections (Fig. 2i, Supplementary Fig. 4c). Taken together, these results indicate that *Uhrf1* is indispensable for spermatogenesis in mice, and that deletion of *Uhrf1* postnatally results in germ cell depletion and male sterility.

### UHRF1 deficiency in testes results in germ cell depletion

Since ablation of UHRF1 in postnatal germ cells caused spermatogenesis failure and male infertility, we next asked when and how germ cells are lost upon deletion of UHRF1 in postnatal testes. By examining the histology of *Uhrf1* cKO and control testes at various developmental time points (P7, 10, 14, 21, 28, 35, and 42), we found the *Uhrf1* cKO testes contained aberrant seminiferous tubules and atrophic tubules with many vacuoles at P14 afterward (Supplementary Fig. 5a). TUNEL assay further revealed that apoptotic cells in *Uhrf1* cKO testes were increased significantly compared with controls from P14 to P35 (Supplementary Fig. 5b, c). These results demonstrate that, upon deletion of *Uhrf1* in testes, germ cells were gradually lost due to apoptosis from P14.

**Fig. 3:**
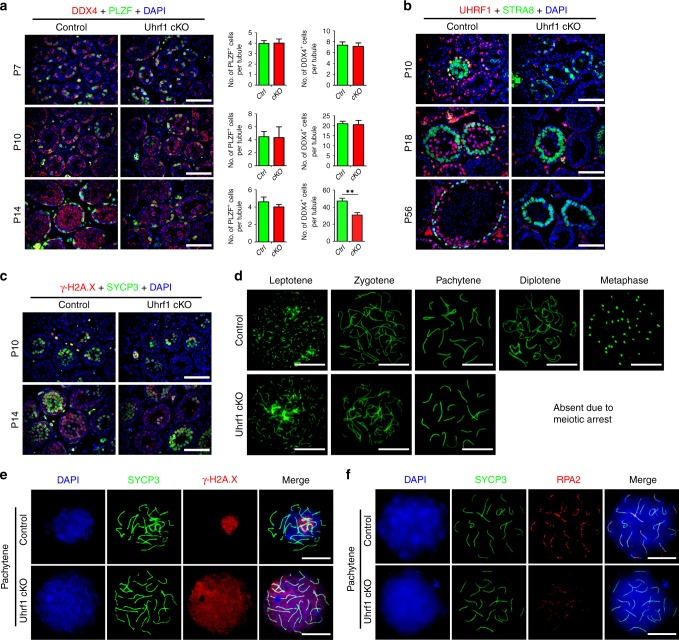
Meiotic arrest at Pachytene stage in *Uhrf1* cKO testes. **a** Co-immunofluorescent staining for the DDX4 (germ cell marker, red) and the PLZF (undifferentiated spermatogonial marker, green) on control and *Uhrf1* cKO mice at P7, 10, and 14 is shown. Nuclei were stained with DAPI. The right histograms analyzed the quantifications of PLZF^+^ cells per tubule and DDX4^+^ cells per tubule at P7, 10, and 14 in Ctrl (control) and *Uhrf1* cKO testes. Mean ± SEM, *n* = 3–7. *P < 0.05 by Student’s *t* test. Scale bar = 100 μm. Source data are provided as a source data file. **b** Co-immunofluorescent staining for the UHRF1 and STRA8 on control and *Uhrf1* cKO testis sections at P10, P18, and P56 are shown. Nuclei were stained with DAPI. Scale bar = 100 μm. **c** Co-immunofluorescent staining for the γ-H2A.X (red) and the SYCP3 (green) on control and *Uhrf1* cKO testis sections at P10 and P14 are shown. Nuclei were stained with DAPI (blue). Scale bar = 100 μm. **d** Labeling of spermatocyte spreads from control and *Uhrf1* cKO testes at P21 stained with SYCP3 are shown. Scale bar = 5 μm. **e** Immunofluorescent staining with anti-SYCP3 and anti-γ-H2A.X on surface-spread pachytene spermatocytes from control and *Uhrf1* cKO mice are shown. Scale bar = 5 μm. **f** Immunofluorescent staining with anti-SYCP3 and anti-RPA2 on surface-spread pachytene spermatocytes from control and *Uhrf1* cKO mice are shown. Scale bar = 5 μm

To determine the exact stage of spermatogenesis where *Uhrf1* cKO germ cells arrested, we analyzed the cell proliferation capacity at various ages of testes by immunofluorescence labeled with PCNA (a cell proliferation marker). However, the abundance of PCNA^+^ cells appeared comparable between controls and *Uhrf1* cKO testes at P7 to P14 (Supplementary Fig. 6a). In addition, the number of PLZF^+^ cells (undifferentiated spermatogonia) is comparable between control and *Uhrf1* cKO testes, but DDX4^+^ cells are significantly decreased at P14 *Uhrf1* cKO testes (Fig. 3a). The number of c-KIT^+^ cells (differentiating spermatogonia) was not affected as well in *Uhrf1* cKO testes at P10 and P14 (Supplementary Fig. 6b). These results suggest that spermatogonial differentiation appeared to be unaffected in *Uhrf1* cKO testes.

To assess whether the meiotic process was disrupted, we examined the meiotic initiation by immunostaining with STRA8 (a marker of differentiating spermatogonia and pre-leptotene spermatocytes) at different postnatal days. We found that the abundance of STRA8^+^ cells in *Uhrf1* cKO testes was comparable with that of controls at different ages, suggesting meiotic initiation is unaffected in *Uhrf1* cKO testes (Fig. 3b, Supplementary Fig. 6c). However, the meiotic spermatocytes, detected with meiotic markers SYCP3 and γ-H2A.X, were significantly decreased in *Uhrf1* cKO testes at P10 and P14 (Fig. 3c), which indicates meiotic arrest. To further determine the sub-stage of meiotic arrest, we stained spermatocyte spreads from *Uhrf1* cKO and control testes with SYCP3 at P21, a time point at which meiosis has been completed and spermatocytes have progressed to haploid spermatids. No diplotene spermatocytes were observed in *Uhrf1* cKO spreads (Fig. 3d). Thus, the *Uhrf1* cKO spermatocytes are arrested at the pachytene stage.

To determine whether the distribution of DNA damage signaling was affected during meiosis, we next examined nuclear distribution of γ-H2A.X using chromosome spreads. In control pachytene spermatocytes, the autosomal synapsis is completed and γ-H2A.X signals are removed from autosomes, while a γ-H2A.X domain is confined to the unsynapsed sex chromosomes. However, γ-H2A.X signals displayed an abnormal distribution in *Uhrf1* cKO pachytene spermatocytes, while autosomal synapsis was completed (Fig. 3e, Supplementary Fig. 7a). To determine whether meiotic recombination was hampered in *Uhrf1* cKO, we performed immunostaining of a replication protein A2 (RPA2) subunit of RPA, a late marker of recombination intermediates^41^, in *Uhrf1* cKO and control spermatocytes. We observed that RPA2 foci appeared to be decreased in mutant spermatocytes (Fig. 3f, Supplementary Fig. 7b), suggesting that meiotic recombination was hampered in *Uhrf1* cKO spermatocytes. In sum, these results indicate that germ cell depletion of *Uhrf1* cKO testes is due to meiotic defects.

### UHRF1 represses retrotransposons in male germ cells

**Fig. 4:**
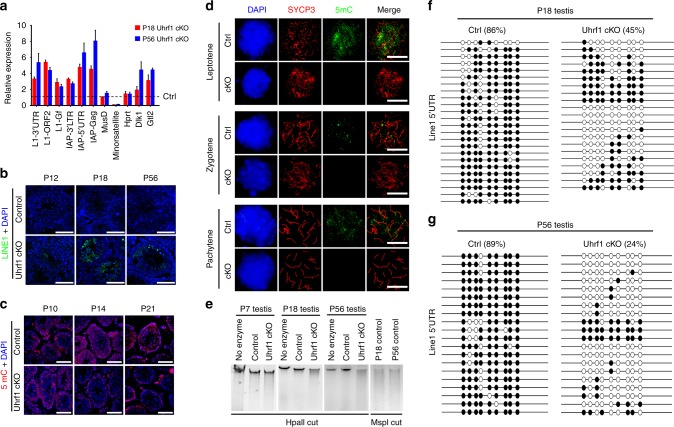
*Uhrf1* ablation in male germ cell leads to retrotransposon activation. **a** RT-qPCR analysis of the expression of retrotransposon (*Line1*, *IAP*), DNA transposon (MusD), peri-centromeric repeats (minor satellites), hypoxanthine-guanine phosphoribosyltransferase (*Hprt*) and parent-of-origin specific imprinted genes (*Dlk1/Gtl2*, *Mirg*) mRNA levels in *Uhrf1* cKO testes at P18 and P56 compared with controls. Data are presented as mean ± SEM, *n* = 3. Source data are provided as a source data file. **b** LINE1 Orf1 expression in control and *Uhrf1* cKO testes at P12, P18, and P56. Scale bar = 50 μm. **c** Immunofluorescent staining with the 5mC for control and *Uhrf1* cKO testes at P10, P14, and P21. Nuclei were stained with DAPI. Scale bar = 50 μm. **d** Immunofluorescent staining with SYCP3 and 5mC for surface-spread spermatocytes from control and *Uhrf1* cKO mice are shown. Scale bar = 5 μm. **e** Genomic DNA was digested with methylation-sensitive HpaII, or its methylation-insensitive isoschizomer (MspI) from control and *Uhrf1* cKO testes at P7, P18, and P56. **f**, **g** DNA from control and *Uhrf1* cKO testes at P18 (**f**) and P56 (**g**) were subjected to bisulfite sequencing of retrotransposon Line1–5′UTR. Methylated CpG sites are shown with black circles and unmethylated sites with open circles. The percentages of overall methylated CpGs are indicated

It is known that elevated transposon activity could result in DNA damage, and UHRF1 has been reported to repress retroviral elements in neural stem cells^42,43^. To investigate whether *Uhrf1* represses retrotransposons in germ cells, we examined retrotransposon transcripts in testes from *Uhrf1* cKO and control mice at P18 and P56 by RT-qPCR. As we expected, a 3–8-fold increase of *Line-1* and *IAP* retrotransposon transcripts were observed in the *Uhrf1* cKO testes compared with that of controls at P18 and P56 (Fig. 4a). Immunostaining revealed that the Line-1 ORF1 protein (ORF1p) was present in *Uhrf1* cKO testes at P18 and persisted at a high level in adulthood (P56: Fig. 4b), confirming the derepression of *Line-1* in *Uhrf1* cKO testes. These data demonstrate that deletion of *Uhrf1* in testes resulted in derepression of retrotransposons, thus highlighting the important role of UHRF1 in silencing retrotransposons in male germ cells. Interestingly, we also found that the mRNA levels of two imprinting genes (*Dlk1* and *Gtl2*) are increased in both P18 and P56 *Uhrf1* cKO testes (Fig. 4a), suggesting that *Uhrf1* is involved in the maintenance of genomic imprinting during spermatogenesis.

### Loss of UHRF1 leads to DNA hypomethylation in germ cells

Having observed the activation of retrotransposon elements, we sought to understand the underlying mechanisms. Given that *Uhrf1* plays a key role in maintaining global DNA methylation during DNA replication, we performed immunostaining to determine the 5-methylcytosine (5mC) level in *Uhrf1* cKO and control testes at P7, P10, P14, and P21 (Fig. 4c, Supplementary Fig. 7c, d). We found that the signals of 5mC are comparable between *Uhrf1* cKO and control testes at P7 and P10, whereas the 5mC levels appear to be significantly reduced in *Uhrf1* cKO testes compared with that of controls at P14 and P21 (Fig. 4c, Supplementary Fig. 7c). We further confirmed that the 5mC signals in differentiated spermatogonia (STRA8 positive cells) were comparable between control and *Uhrf1* cKO testes at P7 (Supplementary Fig. 7d). In contrast, chromosome spread analysis revealed that 5mC signals were significantly reduced in *Uhrf1* cKO spermatocytes (leptotene, zygotene and pachytene stages) compared with those in controls (Fig. 4d). We observed massive global losses of DNA methylation in *Uhrf1* cKO testes at P18 and P56, but not at P7, as evidenced by a decrease in the size of genomic DNA after digestion with HpaII (a methylation-sensitive restriction endonuclease that can cut CCGG sites) (Fig. 4e). These results indicate that global DNA methylation status (5mC) is comparable between *Uhrf1* cKO and control differentiating spermatogonia, but show decreased levels in *Uhrf1* cKO spermatocytes.

To gain further insight into the specific hypo-methylated genomic loci, we detected the methylation levels of retrotransposons by bisulfite DNA sequencing at P18 and P56 testes from *Uhrf1* cKO and control mice (Fig. 4f, g). We observed a decrease of *Line1* element CpG site methylation in testes from ~86% in controls to ~45% in *Uhrf1* cKO mice at P18, and from ~89% in controls to ~24% in *Uhrf1* cKO testes at P56, respectively. Meanwhile, the IAP element CpG site methylation was also decreased from ~90% in controls to ~70% in *Uhrf1* cKO testes at P18 and ~89% in controls to ~64% in *Uhrf1* cKO testes at P56, respectively (Supplementary Fig. 8a, b). Notably, CpG site methylation at the imprinted *Dlk1/Gtl2* domain was not changed in *Uhrf1* cKO testes compared with that of controls either at P18 or P56 (Supplementary Fig. 8c, d). However, we observed a decrease of IG-DMR (intergenic-differentially methylated region) methylation at the *Dlk1/Gtl2* domain in P56 testes from ~93% in controls to ~68% in *Uhrf1* cKO (Supplementary Fig. 8e). Altogether, our data suggest that *Uhrf1* maintains global DNA methylation in spermatocytes and suppresses retrotransposon elements via methylated CpG of *Line-1* and IAP sequences during spermatogenesis.

### UHRF1 is associated with histone modifications in testes

**Fig. 5:**
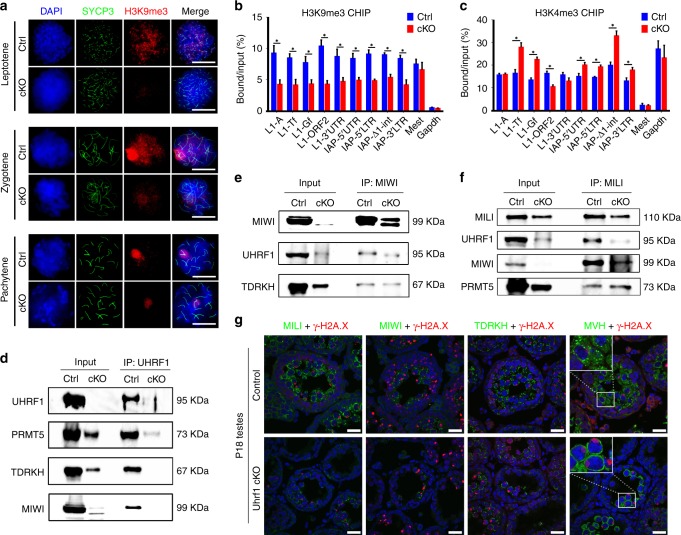
Loss of UHRF1 causes histone modification alteration and PIWI protein ectopic expressions. **a** Immunofluorescent staining with SYCP3 and H3K9me3 for surface-spread spermatocytes from Ctrl (control) and *Uhrf1* cKO mouse testes. Scale bar = 5 μm. **b**, **c** ChIP-qPCR showing the H3K9me3 (**b**) and H3K4me3 (**c**) enrichments at various retrotransposons in Ctrl and *Uhrf1* cKO mouse testes at P21. Quantitative data are expressed as the ratio of the ChIP (Bound) to the input DNA. *Mest* locus was used as a positive control for H3K9me3 and negative control for H3K4me3 enrichment. *Gapdh* promoter was used as a negative control for H3K9me3 and positive control for H3K4me3 enrichment. Error bars indicate the SEM of three biological replicates. *P < 0.05 by Student’s *t*-test. Source data are provided as a source data file. **d** Co-immunoprecipitation of UHRF1 followed by western blot detection of PRMT5, TDRKH, and MIWI from adult control and *Uhrf1* cKO testes. **e** Co-immunoprecipitation of MIWI followed by western blot detection of TDRKH and UHRF1 from adult control and *Uhrf1* cKO testes. **f** Co-immunoprecipitation of MILI followed by western blot detection of UHRF1, MIWI, and PRMT5 from adult control and *Uhrf1* cKO testis extractions. **g** Co-immunostaining of γ-H2A.X with MILI, MIWI, TDRKH, and MVH on P18 testis sections from control and *Uhrf1* cKO mice. DNA was stained with DAPI. Note: MVH (an intermitochondrial cement marker) displayed a diffused pattern in the cytoplasm of *Uhrf1* cKO spermatocytes instead of perinuclear granular mitochondrial localization in controls (see zoom in on images). Scale bar = 20 μm

Since repressive histone marks are associated with the silencing of retrotransposons, we examined H3K9me2 and H3K9me3 levels in control and *Uhrf1* cKO testes. Using chromosome spreads, we observed that both H3K9me2 and H3K9me3 were decreased in *Uhrf1* cKO spermatocytes compared with that of controls (Fig. 5a, Supplementary Fig. 9a). At the same time, we also checked H3K4me3, a permissive histone mark, in control and *Uhrf1* cKO testes, and found that H3K4me3 was increased in *Uhrf1* cKO spermatocytes (Supplementary Fig. 9b). Notably, in control spermatocytes, both H3K9me3 and H3K9me2 were observed in the chromosomes of leptotene and zygotene stages, whereas at the pachytene stage, the H3K9me2 mark was diminished and H3K9me3 was restricted in the XY body (Fig. 5a, Supplementary Fig. 9a), suggesting the repressive H3K9 modification is developmentally regulated by UHRF1 during meiosis. We next detected H3K9me3 and H3K4me3 at retrotransposons in testes by chromatin immunoprecipitation (ChIP)-qPCR assay. In agreement with our immunofluorescence results, the enrichment of H3K9me3 at regulatory sequences of retrotransposons was significantly reduced in *Uhrf1* cKO testes compared with those of controls (Fig. 5b). In contrast, H3K4me3 enrichment was mostly increased at retrotransposons (Fig. 5c). Taken together, H3K9me3 is enriched at retrotransposons and the loss of UHRF1 leads to the decrease of H3K9me3 at retrotransposons.

### UHRF1 interacts with PIWI proteins

Retrotransposon repression and DNA methylation are regulated by the PIWI pathway in the germline^19^. Given the observed phenotype of retrotransposon activation in spermatocytes (Fig. 4a, b), we hypothesized that UHRF1 interacts with PIWI proteins to regulate piRNA biogenesis. To test this hypothesis, we first asked whether UHRF1 interacts with PIWI family proteins in testes. By reciprocal IP examination, we found that UHRF1 interacts with MIWI and MILI in adult testes (Fig. 5d–f, Supplementary Fig. 9c–e for silver staining). Of note, we also determined that UHRF1 is associated with TDRKH (Fig. 5d), another key factor responsible for piRNA biogenesis, which interacts with PIWI proteins^44^. We then assessed PIWI protein expression in *Uhrf1* cKO and control testes by immunostaining. Immunofluorescence revealed that both MIWI and MILI proteins are barely detectable in γ-H2A.X positive spermatocytes of *Uhrf1* cKO, whereas MIWI and MILI are highly expressed in the cytoplasm of control spermatocytes (Fig. 5g). The signal of TDRKH also appeared to be reduced in *Uhrf1* cKO spermatocytes compared with those of controls (Fig. 5g). Interestingly, MVH (an intermitochondrial cement marker) displayed a diffused pattern in the cytoplasm of *Uhrf1* cKO spermatocytes at P18 testes compared with perinuclear granular mitochondrial localization in controls (Fig. 5g), suggesting intermitochondrial cement (IMC) proteins were mislocalized upon *Uhrf1* mutation. Together, these data suggest that UHRF1 interacts with PIWI family proteins and regulates their localization in testes.

We next analyzed piRNA populations by deep-sequenced total small RNAs obtained from P9 and P15 testes, when pre-pachytene piRNA and pachytene piRNAs are enriched, respectively. Testes at these two time points consist of almost identical cell populations between control and *Uhrf1* cKO testes. Size distribution of small RNA analyses revealed that conditional deletion of *Uhrf1* in testes does not alter pre-pachytene piRNA populations at P9, but causes a decrease of pachytene piRNA populations at P15 (Supplemenatary Fig. 9f, g). In all, 25~32 bp reads were reduced by ~80%, and the peak at ~30 bp read (characteristic of pachytene piRNA) was decreased in the *Uhrf1* cKO library at P15 testes, indicating severe defects in pachytene piRNAs production (Supplemenatary Fig. 9g). To further determine the underlying causes of defective piRNA production, we tested whether piRNA transcription is abolished in *Uhrf1* cKO testes. We performed ChIP-seq analysis to profile Pol-II occupancy on the reported 214 piRNA clusters^45,46^ in control and *Uhrf1* cKO testes. Surprisingly, we found that only 22 piRNA precursors displayed a dysregulated RNA Pol-II occupancy in *Uhrf1* cKO testes compared with those of WT testes. 17 piRNA precursors showed upregulated enrichment and 5 piRNA precursors showed downregulated enrichment (Supplementary Fig. 10a–d, Supplementary Data 1). This Pol-II ChIP-Seq analysis indicates that ~10% (22/214) piRNA cluster transcriptional levels are compromised in *Uhrf1* cKO testes. Taken together, these results suggest that UHRF1 cooperates with PIWI proteins and might be involved in piRNA biogenesis but not in piRNA transcription.

### UHRF1 cooperates with PRMT5 in testes

**Fig. 6:**
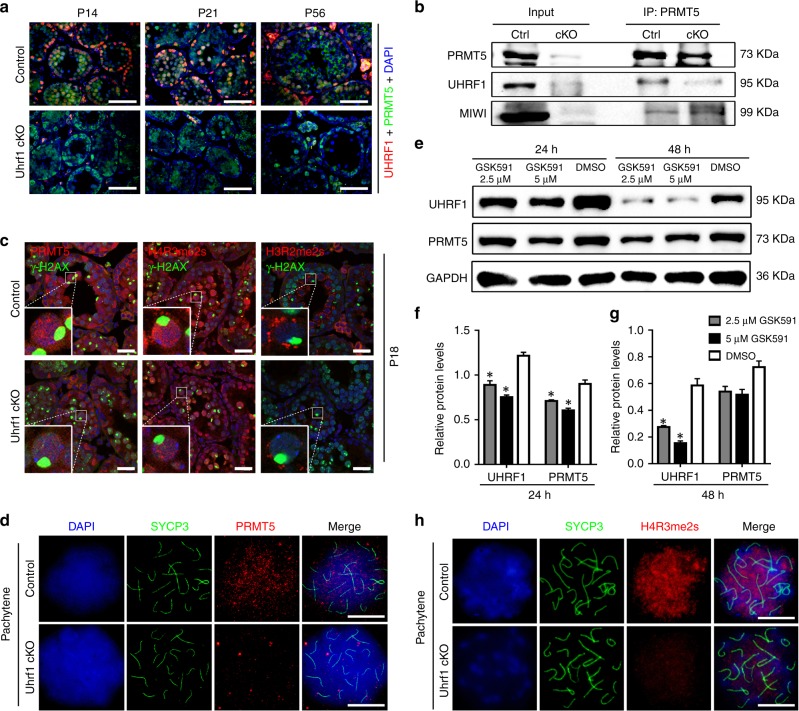
UHRF1 co-localizes with PRMT5 and regulates histone arginine methylations in spermatocytes. **a** Co-immunofluorescent staining with UHRF1 (red) and the PRMT5 (green) on control and *Uhrf1* cKO testes at P14, P21, and P56. Nuclei were stained with DAPI (blue). Scale bar = 50 μm. **b** Co-immunoprecipitation of PRMT5 followed by western blot detection of UHRF1 and MIWI from adult control and *Uhrf1* cKO testes. **c** Co-immunofluorescent staining for the γ-H2A.X with PRMT5, H4R3me2s, H3R2me2s, respectively, in P18 control and *Uhrf1* cKO testes. Nuclei were stained with DAPI (blue). Scale bar = 25 μm. **d** Co-Immunofluorescent staining with PRMT5 and SYCP3 of surface-spread pachytene spermatocytes from control and *Uhrf1* cKO testes. Scale bar = 5 μm. **e** Western blot dection of UHRF1 and PRMT5 protein levels in NIH3T3 cells with treatment of GSK591 (an inhibitor of PRMT5) in different concentration for 24 and 48 h. **f**, **g** Histogram showing the quantification of UHRF1 and PRMT5 levels in (**e**). *P < 0.05 by Student’s *t*-test. Data are presented as mean ± SEM, *n* = 3. Source data are provided as a source data file. **h** Co-Immunofluorescent staining with H4R3me2s and SYCP3 of surface-spread pachytene spermatocytes from control and *Uhrf1* cKO testes. Scale bar = 5 μm

UHRF1 interacts with histone methyltransferase G9a^47^ and other chromatin modification enzymes^35,36^. To elucidate the molecular mechanism by which UHRF1 regulates histone modifications, we sought to determine other histone modifiers with which UHRF1 interacts. Since PRMT5, an arginine methyltransferase, is involved in gene regulation^48,49^ and also plays a role in TEs silencing^25^, we thus hypothesized that UHRF1 cooperates with PRMT5 to repress retrotransposons in the male germline. PRMT5 exhibited a dynamic translocation expression pattern between the nucleus and cytoplasm in the male germ cells as previously reported^50^. PRMT5 was mainly expressed in the cytoplasm of spermatogonia and pre-leptotene spermatocytes; thereafter, PRMT5 was translocated into the nucleus from leptotene to diplotene spermatocytes, expressed in the nucleus of late pathytene stage, then returned to cytoplasm in round spermatids (Supplemenatary Fig. 11a, b). We found that UHRF1 and PRMT5 are mainly co-localized in the nucleus of pachytene spermatocytes (Fig. 6a).

We then performed reciprocal immunoprecipitation (IP) assays in testes using antibodies specific for UHRF1 and PRMT5. In the UHRF1 antibody immunoprecipitants, PRMT5 was strongly detected in control testes (Fig. 5d), and in the PRMT5 antibody immunoprecipitants, UHRF1 was detected in control testes (Fig. 6b). These results confirmed that UHRF1 and PRMT5 are indeed bona fide interacting partners in the testes. In addition, we found both mRNA and protein levels of *Prmt5* are decreased in *Uhrf1* cKO testes at the age of P18 and P56 relative to that of controls by RT-qPCR and Western blot assays (Supplementary Fig. 12a–c), suggesting that *Prmt5* expression is affected upon lack of *Uhrf1* in testes. Furthermore, Co-IP also showed that PRMT5 interacts with MIWI and MILI (Figs. 5f, 6b), which is consistent with previous reports^51^. Interestingly, immunostaining of PRMT5 with γ-H2A.X at P18 seminiferous testis sections showed that the PRMT5 levels were reduced only at pachytene spermatocytes in *Uhrf1* cKOs compared with that of controls (Fig. 6c). This result was further confirmed with chromosome spreads of *Uhrf1* cKO spermatocytes at P18 (Fig. 6d, Supplementary Fig. 12d). UHRF1 protein level reduction was observed in PRMT5 inhibitor GSK591 treated NIH3T3 cells (Fig. 6e–g, Supplementary Fig. 13a), suggesting that PRMT5 could affect UHRF1 localization as well. As PRMT5 mediates symmetrical dimethylation of arginine-3 of histone H4 (H4R3me2s) and arginine-2 of histone H3 (H3R2me2s)^52,53^, we examined whether H4R3me2s and H3R2me2s are affected in *Uhrf1* cKO spermatocytes. In control testis sections at P18, H4R3me2s was detected in the nucleus of different stages of spermatocytes, whereas H3R2me2s was only localized at the cytoplasm of pachytene spermatocytes (Fig. 6c). However, in the *Uhrf1* cKO spermatocytes of P18 testes, both H4R3me2s and H3R2me2s expressions were significantly reduced (Fig. 6c, h, Supplementary Fig. 13b). These data suggest that UHRF1 cooperates with PRMT5 to regulate H4R3me2s/H3R2me2s in spermatocytes.

**Fig. 7:**
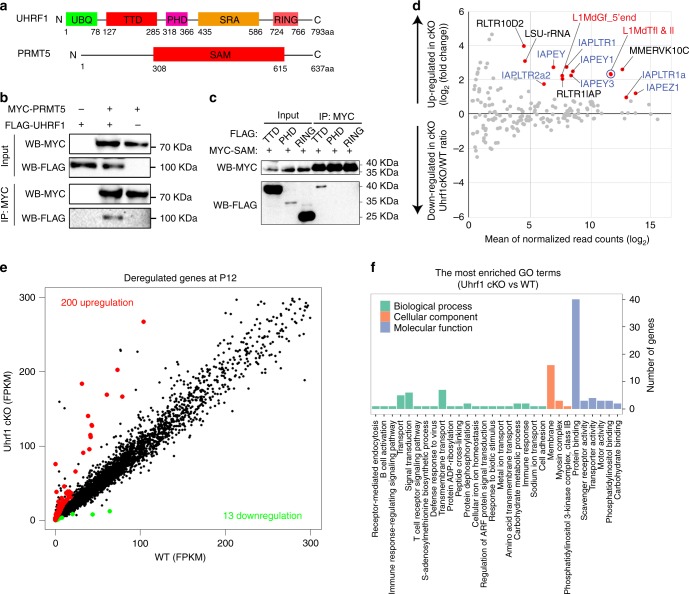
UHRF1 interacts with PRMT5 and regulates gene expression in testes. **a** The schematic illustration of the distinct domains of UHRF1 and PRMT5. **b** UHRF1 interacts with PRMT5. HEK293T cells were transfected with indicated plasmids. After 48 h transfection, immunoprecipitation was performed using anti-MYC antibody. FLAG-tagged UHRF1 and MYC-tagged PRMT5 were detected by western blotting using anti-FLAG and anti-MYC antibodies. **c** The TTD domain of UHRF1 interacts with the SAM domain of PRMT5. HEK293T cells were transfected with indicated plasmids. Forty-eight hours after transfection, immunoprecipitation was performed using anti-MYC antibody. FLAG-tagged UHRF1 fragments and MYC-tagged PRMT5 SAM domain were detected by western blotting using anti-FLAG and anti-MYC antibodies. **d** RNA-seq analysis of retrotransposon repeat elements using Repbase. Fourteen repeat elements were derepressed in P12 testes of *Uhrf1* cKO (shown as red dots; FDR < 0.05, Wald test and > 2 fold change). LINEs are shown in red font, and IAPs are shown in blue font. Datasets for L1Mdtf1 and L1MdtfII are overlapped (shown in a blue circle). **e** Graph plot showing deregulated genes in *Uhrf1* cKO testes at P12. Significantly regulated genes have a *P*-value of < 0.05 and fold change of > 2. Three biological replicates were indicated. **f** Gene ontology term analyses of the 213 deregulated genes from WT control and *Uhrf1* cKO testes at P12

To understand the potential mechanism by which UHRF1 interacts with PRMT5 in vitro, we examined its direct association between UHRF1 and PRMT5. We found that UHRF1 and PRMT5 are mainly co-localized in the nucleus of HEK293T cells (Supplementary Fig. 13c). We then ectopically co-expressed UHRF1 with PRMT5 in HEK293T cells, in which UHRF1 was detected in PRMT5 immunoprecipitates, indicating its ability to interact with PRMT5 proteins (Fig. 7a, b). We further found that the tandem tudor domain (TTD), which binds to H3K9me3 but not other regions of UHRF1, is mainly responsible for interaction with S-adenosylmethionine (SAM) binding domain of PRMT5 (PRMT5 has only one SAM binding domain) (Fig. 7c). These findings suggest that UHRF1 cooperates with PRMT5 through specific domains.

### Global gene expression changes in *Uhrf1* cKO testes

To understand the molecular changes occurring during spermatogenesis in the *Uhrf1* cKO, we next analyzed transcriptional profiling through RNA sequencing. Due to the histology and cell population similarity at P9 and P12 testes in *Uhrf1* cKO and control mice, we used both P9 and P12 testes to perform RNA-Seq. To confirm the derepression of *Line1* and *IAP* retrotransposons shown in Fig. 4a, we analyzed the expression of repeat elements in the P12 testes RNA-seq data. Consistent with the RT-qPCR data, 14 repeat elements containing *Line1* and *IAP* retrotransposon transcripts were significantly upregulated in *Uhrf1* cKO (fold change of >2, FDR *<* 0.05, Wald test: Fig. 7d, Supplementary Data 2).

With regard to the coding genes, a total of 18,358 genes were found to be expressed from WT control and *Uhrf1* cKO testes at P12 (Supplementary Data 3); however, we detected only a few significant changes of coding gene expression (213 genes with fold change of >2) in *Uhrf1* cKO testes compared with that of controls, with 200 upregulated and 13 downregulated genes (Fig. 7e, Supplementary Fig. 14a, Supplementary Data 4). Interestingly, only 20 genes were significantly deregulated at P9 *Uhrf1* cKO testes by RNA-seq analyses (18 genes upregulated and 2 genes downregulated), which suggests the effects caused by *Uhrf1* deletion before meiosis are very subtle (Supplementary Fig. 14b-c, Supplementary Data 5). To validate the RNA-Seq data, we performed RT-qPCR to verify the expression levels of 18 significantly changed genes (14 upregulated and 4 downregulated genes) between control and *Uhrf1* cKO testes at P12. The RT-qPCR fold change results showed that all selected gene expression levels are largely consistent with the RNA-Seq data (Supplementary Fig. 14d). ChIP-qPCR assay further revealed that the enrichment of H3K9me3 at promoter regions of those upregulated genes (*Tex40*, *Ak9*, and *Wdfy1* were selected as representative genes) are significantly reduced in *Uhrf1* cKO testes compared with that of WT controls (Supplementary Fig. 14e). Together, these results suggest that *Uhrf1* represses gene expression via H3K9me3 during spermatogenesis.

To determine whether any molecular functions and biological processes were significantly altered upon lack of *Uhrf1* in testes, we performed Gene Ontology (GO) term analysis for those deregulated genes at P12 testes. The most enriched GO term analysis demonstrated that specific ‘GO terms’, such as signal transduction, membrane, and protein binding are significantly over-presented in *Uhrf1* cKO testes (Fig. 7f, Supplementary Data 6). KEGG analysis revealed that most deregulated genes are related to human diseases and environmental information processing (Supplementary Fig. 15a, Supplementary Data 7). Further gene set enrichment analysis (GSEA) analysis revealed under-presentation of genes related to replication, recombination and repair in *Uhrf1* cKO testes and over-representation of genes related to defense mechanisms (Supplementary Fig. 15b, c), raising the possibility that dysregulation of these genes may compromise meiotic processes in *Uhrf1* cKO testes. Our analyses revealed altered gene expression in *Uhrf1* cKO testes, in which dysregulation of several factors may contribute to meiotic germ cell death in *Uhrf1* cKO testes.

## Discussion

The current study demonstrates that UHRF1 is indispensable for spermatogenesis and participates in silencing retrotransposons during postnatal germ cell development. We report that UHRF1 exhibits a dynamic nuclear-cytoplasmic translocated expression pattern during spermatogenesis. Likewise, PRMT5 has been shown to have a similar dynamic expression pattern between the cytoplasm and nuclei at PGCs and different subtypes of male germ cells^25,50^. However, PRMT5 represses retrotransposons in PGCs, but not in postnatal male germ cells^25,50^, suggesting that an alternative retrotransposon repression mechanism exists in postnatal germ cells beyond the function of PRMT5 in PGCs. In this study, we found that UHRF1 interacts with PRMT5 in testes and regulates symmetric dimethylation of arginine residues. Consistent with our data, a recent study demonstrated that UHRF1 could form a complex with arginine methyltransferase PRMT5 to regulate tumor suppressor genes in endometrial carcinoma^37^. Conditional knockout of PRMT5 mediated by *Blimp1-Cre* in PGCs results in male infertility due to decreased repressive H2A/H4R3me2s marks on TEs and subsequent TEs activation^25^. The current study identified an interplay of UHRF1 and PRMT5 in spermatogenesis, suggesting that histone arginine methylation functions with UHRF1 to silence retrotransposons.

UHRF1 is involved in various processes, including DNA methylation maintenance, heterochromatin formation and gene transcription^29,54^. In the germline, conditional inactivation of *Uhrf1* in growing oocytes leads to embryo preimplantation failure, suggesting that maternal UHRF1 plays a crucial role in early embryonic development^39^. Of note, in this study, we demonstrated that *Uhrf1* is essential for spermatogenesis, which adds another layer to *Uhrf1* function. Data from mouse ESCs have demonstrated that UHRF1 can repress retrotransposons mainly by its role of DNA methylation maintenance coupled with DNA replication^29,30^. Notably, we found a severe defect of *Uhrf1* cKO in meiotic prophase, at which premeotic DNA replication is areadly completed. Interestingly, a recent study showed a delay in establishing replication-dependent maintenance of DNA methylation in premeiotic S phase^55^. Therefore, it is tempting to speculate that UHRF1 has a delayed role in establishing DNA methylation in meiotic prophase at hemimethylated DNA sites derived from premeiotic S phase. Our results demonstrate that UHRF1 localizes on the nuclei of spermatocytes and binds to retrotransposons (Supplementary Fig. 2d, 16a), which enables UHRF1 to regulate retrotransposon repression during spermatogenesis.

Given that UHRF1 is required for the maintenance of DNA methylation during DNA replication, *Stra8-Cre* induced deletion of *Uhrf1* in differentiating spermarmatogonia, and subsequent proliferation could result in near-complete depletion of DNA methylation in meiotic spermatocytes. While our methylation analyses demonstrated depletion of DNA methylation, it was not complete, nor would it be accurate to describe it as near-complete; instead, we observed varied degrees of demethylation at genomic loci. On the other hand, the global DNA methylation status was comparable between *Uhrf1* cKO and control differentiated spermatogonia, at which premeiotic DNA replication has not occured. These results suggest that the loss-of-function of UHRF1 mediated by *Stra8*-Cre took place just prior to entry into meiosis and that loss of DNA methylation in the *Uhrf1* cKO cannot be explained solely by defects in DNA methylation maintenance. Conditional deletion of *Uhrf1* in differentiating spermatogonia leads to meiotic defects and sterility, presumably due to a combination of effects through the loss of DNA methylation, the decrease of histone arginine methylation, and the aberrance of piRNA pathways. In addition, these results further support the rationale that *Stra8-Cre* can be an appropriate Cre driver, as it enabled us to determine a meiotic function for UHRF1 rather than reveal the consequences of DNA methylation loss in progenitor cells. In support of this rationale, a previous study demonstrated that conditional deletion of *Mili*, also via *Stra8-Cre*, resulted in the depletion of MILI in meiotic spermatocytes, while localization of MILI did not change in the mutant spermatogonia^24^.

Both DNA methylation and H3K9 methylation have important roles in the repression of retrotransposons during spermatogenesis. While H3K9 methylation plays a major role in silencing retrotransposons in spermatogonia^23,56^, defective DNA methylation establishment in prospermatogonia of the *Miwi2* and *Dnmt3L* mutants leads to derepression of retrotransposon elements (IAP and LTR) in spermatogonia^57^. Another study using *Dnmt3L*^−*/−*^ male mice showed that DNA methylation has a key role in retrotransposon silencing during meiosis, and that H3K9me2 decreased gradually and disappeared at pachytene stage^58^, as we confirmed in Supplementary Fig. 9a. Interestingly, in WT spermatocytes, the expression level of UHRF1 was very low at the leptotene stage and gradually increased in zygotene and pachytene stages (Fig. 1). Notably, these cells are not at S phase, but UHRF1 seems to have a specific function during meiosis. In this study, we observed that LINE1 was activated in zygotene/pachytene but not in leptotene spermatocytes (Supplementary Fig. 16b), which could explain why meiotic arrest took place at the pachytene stage in *Uhrf1* deficient mice.

Importantly, in this study, we found that UHRF1 could interact with PIWI proteins and that pachytene piRNA profiles were compromised in *Uhrf1* deficient testes, which suggested that UHRF1 might participate in the piRNA pathway to regulate retrotransposons. Interestingly, constitutive knockout of MIWI or the conditional mutation of MILI (*Stra8-Cre* induced) in mice leads to failure to produce pachytene piRNAs and arrest of spermatogenesis at the round spermatid stage^24,59^, whereas our *Stra8-Cre* induced *Uhrf1* knockouts displayed a meiotic arrested phenotype, suggesting that UHRF1 may have a distinct function from MIWI or MILI during meiosis. In fact, in the current study, UHRF1 was observed to interact with several PIWI proteins (MILI and MIWI) and piRNA pathway related proteins (TDRKH and MVH) in testes. Those piRNA related protein signals were significantly reduced when UHRF1 was deleted in germ cells, which may explain the severe phenotype observed in *Uhrf1* cKO mice. Importantly, UHRF1’s TTD domain interacts with PRMT5’s SAM domain (Fig. 7a, b); PIWI proteins (MIWI and MILI) were found in complex with PRMT5/WDR77, an enzyme that dimethylates arginine residues, and arginine methylated through PRMT5^51^. Loss of PRMT5 activity led to a reduction of piRNA levels and mislocalization of PIWI in *Drosophila*^60,61^. Therefore, it is likely that, in the cytoplasm of spermatocytes, UHRF1 forms a complex with PRMT5 and PIWI proteins (MILI/MIWI) to facilitate the slicer activity of PIWI proteins^24,62^, which directly cleaves transposon mRNAs and contributes to the repression of retrotransposons at the posttranscriptional levels. It should be noted, however, in *Uhrf1* cKO testes, dysregulation of PIWI proteins could be the cause of the decrease of pachytene piRNA.

In sum, our study identifies an important role of UHRF1 in retrotransposon repression in relation to DNA methylation, histone modification and piRNA pathway during spermatogenesis. Further, our study suggests that UHRF1 could recruit histone arginine methyltransferase PRMT5 to repress retrotransposons in male spermatocytes, thereby ensuring genome integrity and normal spermatogenesis in mice.

## Methods

### Ethics statement

All the animal procedures were approved by the Institutional Animal Care and Use Committee of Tongji Medical College, Huazhong University of Science and Technology, and the mice were housed in the specific pathogen-free facility of Huazhong University of Science and Technology. All experiments with mice were conducted ethically according to the Guide for the Care and Use of Laboratory Animal guidelines.

### Mice

Floxed *Uhrf1* mice (*Uhrf1*^*flox/flox*^) were generated by embryonic stem cells (ESCs) targeting and blastocyst injection at Shanghai Research Center for Model Organisms. In brief, ESCs were targeted by carrying two loxP sites flanked in Exon 4 and a neomycin selection cassette flanked by FRT sites in intron 4–5 of *Uhrf1*. The *Uhrf1*^+/flox^ mice were obtained by chimera formation and germline transmission. Mice were then crossed with FLP transgenic mice to remove the neomycin cassette and maintained on a C57BL/6J background. *Stra8*-Cre in the C57BL/6J background was purchased from the Jackson Laboratory. S*tra8*-Cre males were first crossed with *Uhrf1*^*flox/flox*^ females to generate the *Stra8*-Cre; *Uhrf1*^+/flox^ males, then the *Stra8*-Cre; *Uhrf1*^+/flox^ male mice were bred with *Uhrf1*^*flox/flox*^ female mice to obtain the *Stra8-Cre; Uhrf1*^*flox/△*^ (designated as *Uhrf1* cKO) males.

### Antibodies

The details of all commercial antibodies used in this study are presented in the Supplementary Table 1. For LINE1 ORF1 antisera generation, the 6× His-LINE1 ORF1 (222-357aa, L1Md-A2, GenBank: M13002.1) fusion protein was expressed in *E. coli* using pQE-30 vector, affinity purified with Ni-NTA resin and used to immunize rabbits, yielding anti-LINE1 ORF1 polyclonal serum for immunofluorescence. We also used the anti-LINE1 ORF1 antibody as a positive control antibody, which was obtained from Prof. Ramesh Pillai (University of Geneva, Switzerland) as a generous gift.

### Histological analysis

Mouse testes and epididymides were collected and fixed in Bouin’s solution (Sigma, HT10132) at 4 °C overnight and then washed with 75% alcohol five times, 30 min each time. Samples were then embedded in paraffin, 5 μm sections were cut and stained with periodic acid-Schiff (PAS) after being dewaxed and rehydrated.

### Immunofluorescence

Testes were fixed in 4% PFA in PBS overnight at 4 °C and then were sequentially soaked in 5, 10, 12.5, 15, and 20% sucrose in PBS and embedded in Tissue-Tek O.C.T. compound (Sakura Finetek, 4583) on dry ice. Embedded samples were stored at −80 °C. Five-micrometer-thick cryosections were cut and washed with PBS three times. To perform antigen retrieval, cryosections were microwaved in 0.01 M sodium citrate buffer (pH 6.0) then cooled down to room temperature. After washing with PBS three times, the sections were blocked in blocking solution (containing 3% normal goat serum and 3% fetal bovine serum in 1% bovine serum albumin) for 1 h. Later, tissue sections were incubated with primary antibodies (Supplementary Table 1) in blocking solution overnight at 4 °C. Slides were then incubated with secondary antibody (Supplementary Table 1) for 1 h at room temperature after washing with PBS and mounted using Vectorshield mounting media with DAPI (H1200, Vector laboratories). Laser confocal scanning images were captured using FluoView 1000 microscope (Olympus, Japan) with digital camera (MSX2, Micro-shot Technology Limited, China).

### Western blotting

Testes tissues were collected and proteins were extracted by using RIPA buffer (CWBIO, Cat# 01408). In total, 18–20 μg of protein lysates were separated on a 10% SDS-PAGE gel, proteins were transferred to PVDF membranes (Bio-Rad) and the membranes were blocked in 5% non-fat milk (blocking solution) for 1 h. Primary antibodies (Supplementary Table 1) were incubated overnight at 4 °C after blocking. The membranes were washed with TBST three times and then incubated with a secondary antibody (Supplementary Table 1) for 1 h before using Luminol/enhancer solution and Peroxide solution (Clarity^TM^ Western ECL Substrate, Bio-Rad). Uncropped versions of all blots are included as Supplementary Figs. 17 and 18.

### Transmission electron microscopy (TEM)

Adult testes from control and *Uhrf1* cKO were fixed in 0.1 M cacodylate buffer (pH = 7.4) containing 3% paraformaldehyde and 3% glutaraldehyde plus 0.2% picric acid for 2 h in 4 °C, then for 1 h at RT. Following three washes with 0.1 M cacodylate buffer, the samples were post-fixed with 1% OsO_4_ for 1 h at RT. Then the samples were dehydrated in sequentially ethanol solutions (30, 50, 70, 90, and 100%) and embedded in Eponate mixture (Electron Microscopy Sciences, Hatfield, PA, USA) for polymerization about 24 h at 60 °C. Ultrathin sections (~70 nm) were cut with a diamond knife. The sections were re-stained with uranyl acetate and lead citrate, and then photographed using a transmission electron microscope (FEI Tecnai G2 12, Holland).

### TUNEL staining

Testes were fixed in Bouin’s solution, embedded in paraffin and sectioned (5 μm). TUNEL staining was performed using In Situ Cell Death Detection kit, Fluorescein (Roche). Briefly, paraffin sections were dewaxed and rehydrated, then immersed in Tris-HCl (0.1 M, pH 7.5) containing 3% BSA and 20% fetal bovine serum and incubated with the TUNEL reaction Mixture for 1 h at 37 °C, DNA was stained with DAPI. Images were obtained with a FluoView 1000 microscope (Olympus, Japan).

### Meiotic chromosome spreads assay

Testes were collected from mice at age P21 and placed in PBS. The tunica albuginea was removed, and seminiferous tubules gently teased apart in a hypotonic extraction buffer (30 mM sucrose, 17 mM trisodium citrate dehydrate, 5 mM EDTA, 0.5 mM DTT, and 0.5 mM PMSF, pH = 8.2) for 1 h at room temperature. Several seminiferous tubules were placed in 100 mM sucrose (pH = 8.2) droplets (about 40 μl) and pipetted gently to release germ cells. Tubular remnants were removed and the remaining suspension was added on the edge of a slide coated with fresh fixative solution (1% PFA, 0.15% Triton X-100, 10 mM sodium borate pH = 9.2). Slide direction was changed and the droplet allowed to drip along the length of the slide. The prepared slides were placed in a humidified chamber at 4 °C overnight. The slides were then washed with 0.4% Photo-Flo (Kodak, 1464510), dried well and stored at −20 °C until use. For immunofluorescence analysis, the procedure was the same as tissue immunofluorescence above.

### Immunoprecipitation

Fresh mouse adult testes from indicated genotypes were dissected and lysed in IP buffer (20 mM HEPES, 150 mM NaCl, 2 mM magnesium acetate, 0.2% NP-40, 1 mM DTT, pH = 7.3), clarified by centrifugation at 12,000 × *g*, and then pre-cleared with protein A beads (Bio-Rad, 161-4013). The lysate was incubated with primary antibodies overnight at 4 °C on a rotator and conjugated with Protein A beads. The beads were washed with IP buffer and then boiled in 2× SDS loading buffer for western blotting analysis. The primary and secondary antibodies are listed in Supplementary Table 1.

### Silver staining

After finished the electrophoresis of proteins in SDS gels, the prepared SDS gels were fixed in fixation solution (50% ethanol, 10% acetic acid, 40% water) for 20 min with gentle shaking. Then the protein was treated with solution mixtures (20% ethanol, 5% acetic acid, 75% water, 4 mg dithiothreitol) and 0.5% dichromate solution, followed by staining with 0.1% silver nitrate solution for 30 min. After washing in water for 30 min, the protein gels were fixed in 0.02% paraformaldehyde + 3% Na_2_CO_3_ (pH = 12) for 10 min. All steps were performed at room temperature and the gels were gently shaken in a glass container.

### In vitro co-immunoprecipitation

Partial *Uhrf1* cDNA fragments were cloned into the pcMV vector encoding a FLAG-tag. *Prmt5* cDNA fragments were cloned into the pcMV vector encoding a MYC-tag. HEK293T cells (obtained from Stem cell Bank of Chinese Academic Science, Cat# GNHu43) were transfected with indicated plasmids using Lipofectamine 2000 (Life Technologies). After 48 h, immunoprecipitation was performed. FLAG- tagged or MYC-tagged proteins were detected by western blotting using anti-FLAG antibody (1:1000; 20543-I-AP, Protein Tech) and anti-MYC antibody (1:10,000; 60003-2-Ig, Protein Tech).

### Inhibitor treatment assay

NIH3T3 cells (obtained from Stem cell bank of Chinese Academic Science, Cat# GNM6) were incubated with 2.5 μM, 5 μM GSK591 (Sigma, SML1751) or DMSO for 24 and 48 h prior to harvest. After incubation, cell proteins were extracted using RIPA buffer (CWBIO, Cat# 01408), and western blot was performed using standard protocols.

### RNA isolation and quantitative RT-PCR

Total RNAs were extracted from testes using TRIzol reagent (Invitrogen) following the manufacturer’s procedure. The purity and concentration of RNA samples were determined with the use of a Nanodrop ND-2000 spectrophotometer (Thermo Scientific). Reverse transcriptional reactions contained 500 ng of purified total RNA using a PrimeScript RT reagent kit with gDNA Eraser (TaKaRa) to remove the DNA contamination. RT-qPCR was performed with SYBR green master mix (TaKaRa) on LightCycler@96 Real-Time PCR system (Roche) according to manufactures’ instructions. The primers are listed in Supplementary Table 2. The relative gene expression was quantified using the comparative cycle threshold method, with the *Gapdh* expression used for normalization.

### Genomic DNA isolation and bisulfite sequencing

The control and *Uhrf1* cKO testes at P18 and P56 were isolated and directly subjected to proteinase K digestion. Genomic DNAs were isolated using the Trelief Animal Genomic DNA Kit (TSINGKE). The EpiTect Bisulfite Kit (QIAGEN) was used for bisulfite treatment of genomic DNA (200 ng) according to the manufacturers’ instructions. The PCR amplification of different loci was performed with an EpiTaq HS (TaKaRa) under the following conditions: denaturation at 98 °C for 60 s and 35 cycles each of 98 °C for 10 s, 55 °C for 30 s, and 72 °C for 45 s. The primers used are listed in Supplementary Table 2. The PCR products were subcloned into the pMD 19-T Vector Cloning Kit (TaKaRa), and individual clones were sequenced. The methylation status of the region (higher than 95% sequence identity) was determined and analyzed with QUMA (http://quma.cdb.riken.jp/top/quma_main_j.html).

### RNA-Seq analysis

Total RNA was isolated from P9 and P12 mouse testes (three biological repeats for control and *Uhrf1* cKO, respectively) using TRIzol reagents (Invitrogen). The RNA concentration was verified using a NanoDrop 2000 Spectrophotometer (Thermo Fisher Scientific). One microgram of total RNA was used from each sample to prepare the mRNA libraries using TruSeq Stranded mRNA Library Preparation Kit Set A (Cat. No. RS-122-2101, Illumina) according to the manufacturers’ instructions. All libraries were sequenced using the Illumina HiSeq 4000 platform. The FASTX-Toolkit was used to remove adaptor sequences and low quality reads from the sequencing data. To identify all the transcripts, we used Tophat2 and Cufflinks to assemble the sequencing reads based on the UCSC MM10 mouse genome. The differential expression analysis was performed by Cuffdiff. The differential expressed genes were set with the threshold of *P* < 0.05 and fold change ≥ 2.

### Small RNA libraries and bioinformatics

To prepare small RNA libraries, three biological testis samples at P9 and P15 from control and *Uhrf1* cKO mice, respectively, were collected and total RNAs were extracted using TRIzol reagent (Invitrogen), then the RNA quality was verified with the use of Agilent 2100 Bioanalyzer Eukaryote Total RNA Pico assay (Agilent Technologies). Small RNAs ranging in size from 15 to 40 nt were gel-purified on a TBE-urea gel and used for library construction with NEBNext DNA Library Prep Reagent Set for Illumina (NEB). The clones were supplied for the cluster generation and sequenced using BGISEQ platform (BGI Genomics Co., Ltd.). Linker sequences and sequencing adapters were removed using *fastx_clipper*. Degradation products were removed by mapping with Bowtie, allowing two mismatches to a library consisting of tRNA, rRNA, scRNA, scpRNA, and snoRNA with 25 bp extensions on both 5′ and 3′ ends. Clipped reads were filtered by length (24–32 nt, unless otherwise indicated). Sequences were annotated using UCSC mm10 (extracted from http://genome.ucsc.edu).

### RNA-seq analysis of repetitive elements

All mouse repetitive element sequences, including ancestral shared repeats (1194 loci), were downloaded from Repbase (http://www.girinst.org/repbase/). A HiSAT2 index was created using the function “hisat2-build.” Raw RNA-seq reads were aligned to repetitive sequences using the default parameters of HiSAT2. A table of aligned read counts per repetitive element was extracted from sorted bam files using the SAMtools function “idxstats.” A read counts output file was input to the DESeq2 package; comparison of expression levels of each repetitive element between WT control and *Uhrf1* cKO was performed using the functions “DESeqDataSetFromMatrix” and “DESeq.” Differentially expressed repetitive elements between WT control and *Uhrf1* cKO were identified by two criteria: >2 fold change and Wald test (FDR < 0.05).

### ChIP (chromatin immunoprecipitation)

Testes were dissected out of three 21–23 dpp mice per genotype. After removing the tunica albuginea from the testes, seminiferous tubules were untangled with fine tissue forceps and transferred into KREB buffer containing 0.5 mg/mL collagenase (Sigma) and incubated, shaking, at 33 °C for 10–15 min (At the end, the tubules should have a “spaghetti-like” appearance). The second digestion was performed with 1 μg/mL DNase (Sigma) to prevent cells from clumping and 0.5 mg/ml trypsin (Sigma) for 20 min, using a wide bore pipette to disperse the tubules into a single cell suspension. The single cell suspension was filtered through a 40 μm filter mesh cell strainer, and centrifuged at 800 × *g* for 15 min. For each ChIP experiments, 1 × 10^5^–3 × 10^5^ testicular cells were used. To crosslink proteins to DNA, cells were fixed in 1% formaldehyde and incubated for 10 min at room temperature, then quenched in glycine for 5 min. Suspension cells were centrifuged at 800 × *g* for 5 min and pellets washed two times with ice-cold PBS, then resuspended in buffer A plus PIC (protease inhibitor cocktail) and DTT (Dithiothreitol) from SimpleChIP® Plus Enzymatic Chromatin IP Kit (Magnetic Beads) #9005. Nuclei were incubated on ice for 10 min, pelleted by centrifugation at 800 × *g* for 5 min. The supernatant was removed and pellets resuspended in buffer B plus DTT. Micrococcal Nuclease was added and incubated for 20 min at 37 °C with frequent mixing to digest DNA to length of approximately 150–900 bp. Nuclei were pelleted by centrifugation at 15,000 × *g* for 1 min, and the nuclear pellet resuspended in ChIP Buffer plus PIC and lysate sonicated with UH-100B Ultrasonic processor (Chincan) for 5 min (15 s “on” and 15 s “off”) on ice to break nuclear membrane. Immunoprecipitation was performed with H3K9me3, H3K4me3, UHRF1, Pol-II, and IgG using the kit as described above. Immunoprecipitated and input DNAs were analyzed by real-time SYBR Green qPCR (primers listed in Supplementary Table 2) or deep sequencing (for Pol-II CHIP).

### CHIP-Seq analysis

Raw reads were processed to high-quality using Trimmomatic (http://www.usadellab.org/cms/index.php?page = trimmomatic) to remove adapters and performed trimming. Trimmed reads were mapped to the Mouse Genome Overview GRCm38 assembly (USCS mm10) (http://ftp.ensembl.org/pub/release-87/fasta/mus_musculus/dna/) using STAR (https://github.com/alexdobin/STAR/releases) with default parameters and only uniquely aligned sequences were retained. DeepTools (http://deeptools.ie-freiburg.mpg.de/) was used for normalization to generate read density plot from BAM or bigwig files. For Pol-II occupancy, peak calling was performed by MACS2 (https://github.com/taoliu/MACS), with input used as the control. For MACS2, default parameters with broad peak option and a broad-cutoff of 0.05 (*P* value) were used.

### Methylation-specific restriction enzyme aasays

Genomic DNA was digested with HpaII (Thermo Fisher Scientific, #FD0514) or MspI (NEB, #R0106L) restriction enzymes overnight at 37 °C and analyzed in a 1.2% agarose gel.

### Statistical analysis

All data are presented as mean ± SEM unless otherwise noted in the figure legends. Statistical differences between datasets were assessed by one-way ANOVA or Student’s *t*-test using the SPSS16.0 software. *P*-values are denoted in figures by *P < 0.05; **P < 0.01; and ***P < 0.001.

### Reporting summary

Further information on research design is available in the Nature Research Reporting Summary linked to this article.

## Supplementary information

Supplementary Information

Peer Review

Reporting Summary

Description of Additional Supplementary Files

Supplementary Data 1

Supplementary Data 2

Supplementary Data 3

Supplementary Data 4

Supplementary Data 5

Supplementary Data 6

Supplementary Data 7

Source Data

## Data Availability

A reporting summary for this Article is available as a Supplementary Information file. All RNA sequencing data are deposited in the NCBI SRA (Sequence Read Achieve) database with the accession number of SRP151332. The source data are provided in Supplementary Figs. 17, 18 and as a source data file. All data are available from the corresponding author upon reasonable request.

## References

[1] Burns KH, Boeke JD (2012). Human transposon tectonics. Cell.

[2] Cordaux R, Batzer MA (2009). The impact of retrotransposons on human genome evolution. Nat. Rev. Genet.

[3] Ostertag EM, Kazazian HH (2001). Biology of mammalian L1 retrotransposons. Annu Rev. Genet.

[4] Bannert N, Kurth R (2004). Retroelements and the human genome: new perspectives on an old relation. Proc. Natl Acad. Sci. USA.

[5] Zamudio N, Bourc’his D (2010). Transposable elements in the mammalian germline: a comfortable niche or a deadly trap?. Heredity.

[6] Rowe HM, Trono D (2011). Dynamic control of endogenous retroviruses during development. Virology.

[7] Henderson IR, Jacobsen SE (2007). Epigenetic inheritance in plants. Nature.

[8] Schaefer CB, Ooi SK, Bestor TH, Bourc’his D (2007). Epigenetic decisions in mammalian germ cells. Science.

[9] Vaissiere T, Sawan C, Herceg Z (2008). Epigenetic interplay between histone modifications and DNA methylation in gene silencing. Mutat. Res..

[10] Stancheva I (2005). Caught in conspiracy: cooperation between DNA methylation and histone H3K9 methylation in the establishment and maintenance of heterochromatin. Biochem. Cell Biol..

[11] Ikegami K (2007). Genome-wide and locus-specific DNA hypomethylation in G9a deficient mouse embryonic stem cells. Genes Cells.

[12] Epsztejn-Litman S (2008). De novo DNA methylation promoted by G9a prevents reprogramming of embryonically silenced genes. Nat. Struct. Mol. Biol..

[13] Kato Y (2007). Role of the Dnmt3 family in de novo methylation of imprinted and repetitive sequences during male germ cell development in the mouse. Hum. Mol. Genet.

[14] Bourc’his D, Bestor TH (2004). Meiotic catastrophe and retrotransposon reactivation in male germ cells lacking Dnmt3L. Nature.

[15] Okano M, Bell DW, Haber DA, Li E (1999). DNA methyltransferases Dnmt3a and Dnmt3b are essential for de novo methylation and mammalian development. Cell.

[16] Matsui T (2010). Proviral silencing in embryonic stem cells requires the histone methyltransferase ESET. Nature.

[17] Bulut-Karslioglu A (2014). Suv39h-dependent H3K9me3 marks intact retrotransposons and silences LINE elements in mouse embryonic stem cells. Mol. Cell.

[18] Karimi MM (2011). DNA methylation and SETDB1/H3K9me3 regulate predominantly distinct sets of genes, retroelements, and chimeric transcripts in mESCs. Cell Stem Cell.

[19] Iwasaki YW, Siomi MC, Siomi H (2015). PIWI-Interacting RNA: Its Biogenesis and Functions. Annu. Rev. Biochem..

[20] Grivna ST, Pyhtila B, Lin H (2006). MIWI associates with translational machinery and PIWI-interacting RNAs (piRNAs) in regulating spermatogenesis. Proc. Natl Acad. Sci. USA.

[21] Carmell MA (2007). MIWI2 is essential for spermatogenesis and repression of transposons in the mouse male germline. Dev. Cell.

[22] Kuramochi-Miyagawa S (2004). Mili, a mammalian member of piwi family gene, is essential for spermatogenesis. Development.

[23] Pezic D, Manakov SA, Sachidanandam R, Aravin AA (2014). piRNA pathway targets active LINE1 elements to establish the repressive H3K9me3 mark in germ cells. Genes Dev..

[24] Di Giacomo M (2013). Multiple epigenetic mechanisms and the piRNA pathway enforce LINE1 silencing during adult spermatogenesis. Mol. Cell.

[25] Kim S (2014). PRMT5 protects genomic integrity during global DNA demethylation in primordial germ cells and preimplantation embryos. Mol. Cell.

[26] Arita K, Ariyoshi M, Tochio H, Nakamura Y, Shirakawa M (2008). Recognition of hemi-methylated DNA by the SRA protein UHRF1 by a base-flipping mechanism. Nature.

[27] Avvakumov GV (2008). Structural basis for recognition of hemi-methylated DNA by the SRA domain of human UHRF1. Nature.

[28] Berkyurek AC (2014). The DNA methyltransferase Dnmt1 directly interacts with the SET and RING finger-associated (SRA) domain of the multifunctional protein Uhrf1 to facilitate accession of the catalytic center to hemi-methylated DNA. J. Biol. Chem..

[29] Bostick M (2007). UHRF1 plays a role in maintaining DNA methylation in mammalian cells. Science.

[30] Sharif J (2007). The SRA protein Np95 mediates epigenetic inheritance by recruiting Dnmt1 to methylated DNA. Nature.

[31] Rothbart SB (2012). Association of UHRF1 with methylated H3K9 directs the maintenance of DNA methylation. Nat. Struct. Mol. Biol..

[32] Liu X (2013). UHRF1 targets DNMT1 for DNA methylation through cooperative binding of hemi-methylated DNA and methylated H3K9. Nat. Commun..

[33] Zhao Q (2016). Dissecting the precise role of H3K9 methylation in crosstalk with DNA maintenance methylation in mammals. Nat. Commun..

[34] Meilinger D (2009). Np95 interacts with de novo DNA methyltransferases, Dnmt3a and Dnmt3b, and mediates epigenetic silencing of the viral CMV promoter in embryonic stem cells. EMBO Rep..

[35] Unoki M, Nishidate T, Nakamura Y (2004). ICBP90, an E2F-1 target, recruits HDAC1 and binds to methyl-CpG through its SRA domain. Oncogene.

[36] Achour M (2009). UHRF1 recruits the histone acetyltransferase Tip60 and controls its expression and activity. Biochem. Biophys. Res. Commun..

[37] Sheng Y (2016). Methylation of tumor suppressor gene CDH13 and SHP1 promoters and their epigenetic regulation by the UHRF1/PRMT5 complex in endometrial carcinoma. Gynecol. Oncol..

[38] Branscombe TL (2001). PRMT5 (Janus kinase-binding protein 1) catalyzes the formation of symmetric dimethylarginine residues in proteins. J. Biol. Chem..

[39] Maenohara S (2017). Role of UHRF1 in de novo DNA methylation in oocytes and maintenance methylation in preimplantation embryos. PLoS Genet..

[40] Sadate-Ngatchou PI, Payne CJ, Dearth AT, Braun RE (2008). Cre recombinase activity specific to postnatal, premeiotic male germ cells in transgenic mice. Genesis.

[41] Moens PB (2002). The time course and chromosomal localization of recombination-related proteins at meiosis in the mouse are compatible with models that can resolve the early DNA-DNA interactions without reciprocal recombination. J. Cell Sci..

[42] Siomi MC, Sato K, Pezic D, Aravin AA (2011). PIWI-interacting small RNAs: the vanguard of genome defence. Nat. Rev. Mol. Cell Biol..

[43] Ramesh V (2016). Loss of Uhrf1 in neural stem cells leads to activation of retroviral elements and delayed neurodegeneration. Genes Dev..

[44] Saxe JP, Chen M, Zhao H, Lin H (2013). Tdrkh is essential for spermatogenesis and participates in primary piRNA biogenesis in the germline. EMBO J..

[45] Zhou L (2017). BTBD18 regulates a subset of piRNA-generating loci through transcription elongation in mice. Dev. Cell.

[46] Li XZ (2013). An ancient transcription factor initiates the burst of piRNA production during early meiosis in mouse testes. Mol. Cell.

[47] Kim JK, Esteve PO, Jacobsen SE, Pradhan S (2009). UHRF1 binds G9a and participates in p21 transcriptional regulation in mammalian cells. Nucleic Acids Res..

[48] Di Lorenzo A, Bedford MT (2011). Histone arginine methylation. FEBS Lett..

[49] Zhao Q (2009). PRMT5-mediated methylation of histone H4R3 recruits DNMT3A, coupling histone and DNA methylation in gene silencing. Nat. Struct. Mol. Biol..

[50] Wang, Y. B. et al. Prmt5 is required for germ cell survival during spermatogenesis in mice. *Sci Rep-Uk* **5**, 11031 (2015). 10.1038/srep11031 PMC4466585 26072710

[51] Vagin VV (2009). Proteomic analysis of murine Piwi proteins reveals a role for arginine methylation in specifying interaction with Tudor family members. Genes Dev..

[52] Ancelin K (2006). Blimp1 associates with Prmt5 and directs histone arginine methylation in mouse germ cells. Nat. Cell Biol..

[53] Migliori V (2012). Symmetric dimethylation of H3R2 is a newly identified histone mark that supports euchromatin maintenance. Nat. Struct. Mol. Biol..

[54] Xie S, Jakoncic J, Qian C (2012). UHRF1 double tudor domain and the adjacent PHD finger act together to recognize K9me3-containing histone H3 tail. J. Mol. Biol..

[55] Gaysinskaya V (2018). Transient reduction of DNA methylation at the onset of meiosis in male mice. Epigenetics Chromatin.

[56] Di Giacomo M, Comazzetto S, Sampath SC, Sampath SC, O’Carroll D (2014). G9a co-suppresses LINE1 elements in spermatogonia. Epigenetics Chromatin.

[57] Vasiliauskaite L (2018). Defective germline reprogramming rewires the spermatogonial transcriptome. Nat. Struct. Mol. Biol..

[58] Zamudio N (2015). DNA methylation restrains transposons from adopting a chromatin signature permissive for meiotic recombination. Genes Dev..

[59] Deng W, Lin H (2002). *miwi*, a murine homolog of *piwi*, encodes a cytoplasmic protein essential for spermatogenesis. Dev. Cell.

[60] Kirino Y (2009). Arginine methylation of Piwi proteins catalysed by dPRMT5 is required for Ago3 and Aub stability. Nat. Cell Biol..

[61] Nishida KM (2009). Functional involvement of Tudor and dPRMT5 in the piRNA processing pathway in *Drosophila* germlines. EMBO J..

[62] Reuter M (2011). Miwi catalysis is required for piRNA amplification-independent LINE1 transposon silencing. Nature.

