# Supplementary material for: UHRF1 suppresses retrotransposons and cooperates with PRMT5 and PIWI proteins in male germ cells
Source: Nat Commun. 2019 Oct 17;10:4705. doi: 10.1038/s41467-019-12455-4 (PMC6797737; doi:10.1038/s41467-019-12455-4)
Supplement: Supplementary file 1 — Supplementary Information [file 41467_2019_12455_MOESM1_ESM.pdf]

UHRF1 suppresses retrotransposons and cooperates with PRMT5 and PIWI proteins in male germ cells

Juan Dong, et.al.

SUPPLEMENTARY INFORMATION

Supplementary Figure 1

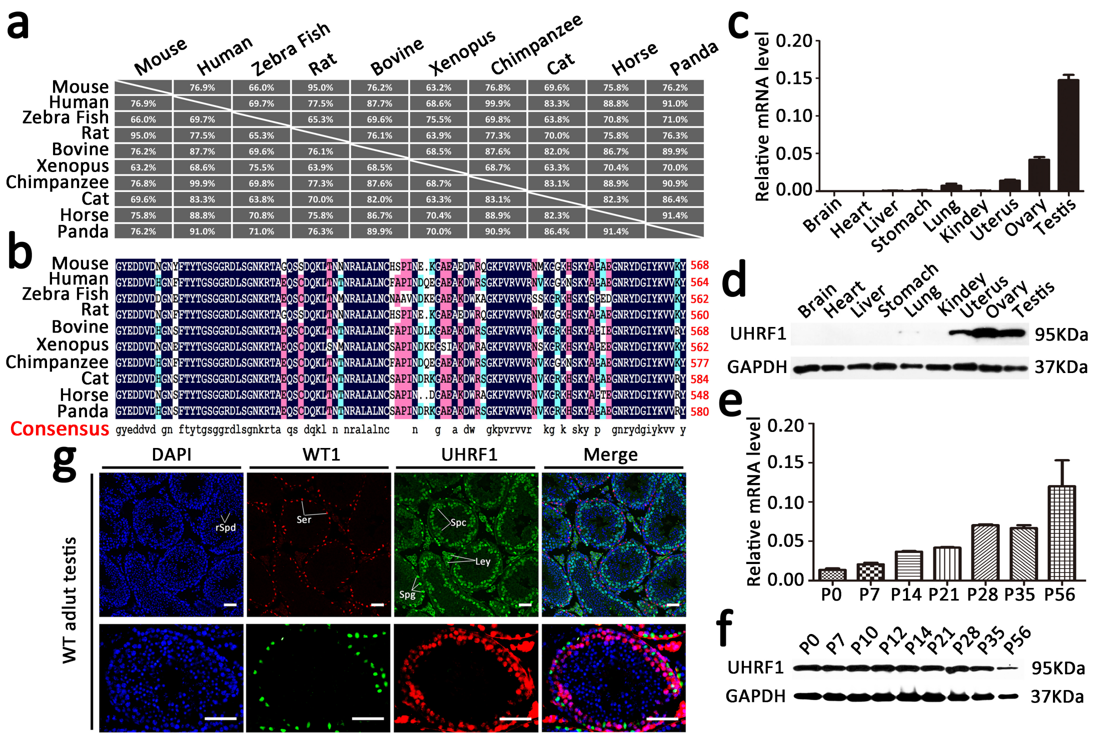

Supplementary Fig.1. Expression profiles of UHRF1 during testicular development and spermatogenesis in mice. (**a-b**) A high degree of conservation of UHRF1 in amino acid sequences among 10 species. (**c**) RT-qPCR analyses of *Uhrf1* mRNA levels in multiple organs in mice. Data are presented as mean ± SEM, n = 3. (**d**) Expression of UHRF1 protein in multiple organs. Levels of UHRF1 in multiple organs were determined using western blot analyses. GAPDH was used as a loading control. (**e**) RT-qPCR analyses of *Uhrf1* mRNA levels in developing testes at postnatal day 0 (P0), P7, P14, P21, P28, P35, and in P56. Data are presented as mean ± SEM, n = 3. (**f**) Expression of UHRF1 protein in developing testes by western blot analyses. GAPDH served as a loading control. (**g**) Double immunofluorescence detection showing UHRF1 was mainly localized in the nucleus of spermatogonia (Spg), Spermatocytes (Spc) and Leyding cells (Ley) in adult WT testes, but not presented in Sertoli cells (Ser). Scale bar=50μm.

Supplementary Figure 2

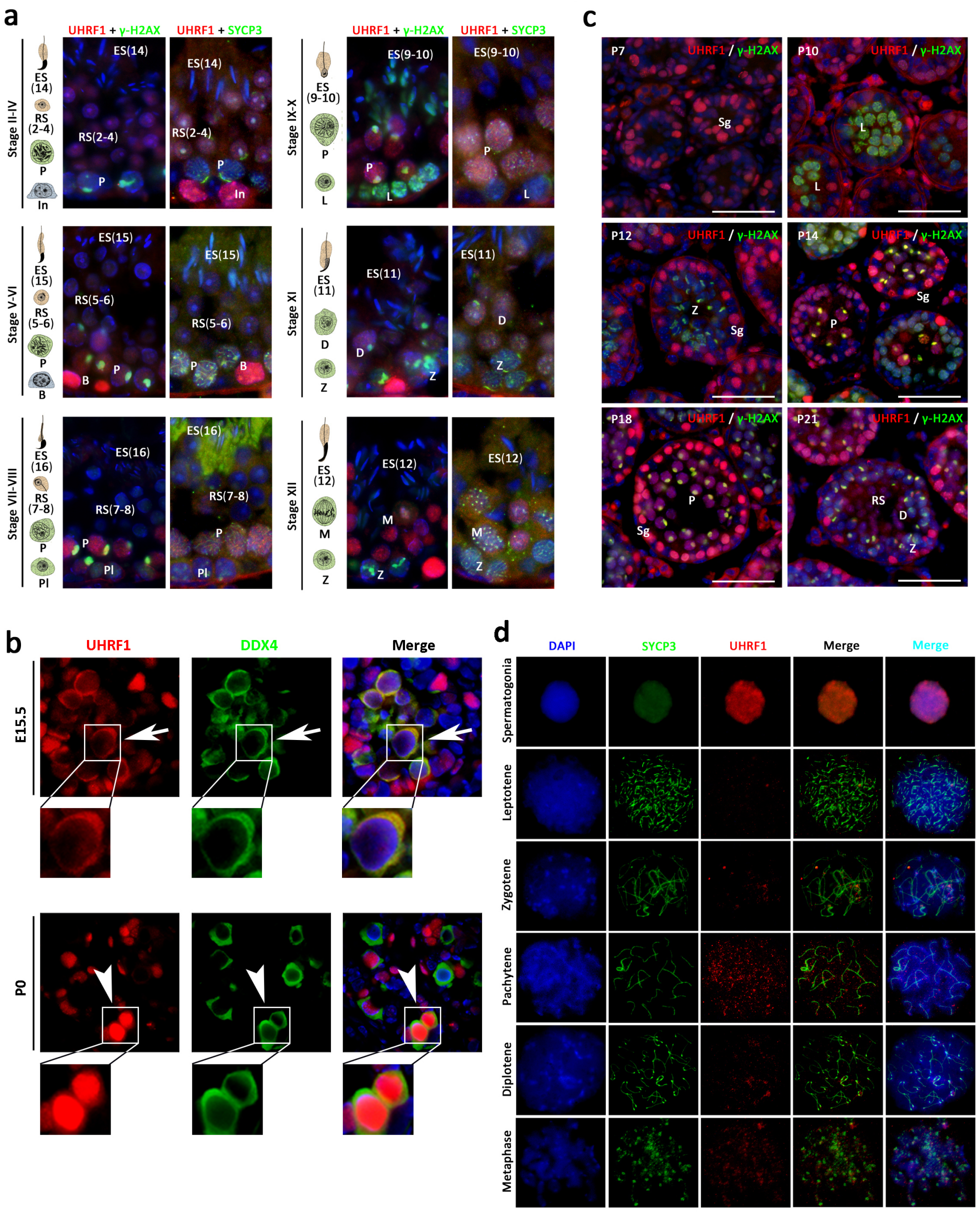

Supplementary Fig.2. Expression of UHRF1 in fetal testes, adult testes and the first wave of spermatogenesis related to Fig.1. (**a**) Co-immunofluorescence staining with anti-UHRF1, anti-γ-H2A.X and anti-SYCP3 antibodies on staged WT testis sections is shown. The left accompanying diagram indicates the spermatogenic content of the seminiferous tubules for the indicated stages. Nuclei were stained with DAPI. (**b**) Co-immunofluorescence using anti-UHRF1 and anti-DDX4 antibodies on WT sections from E15.5 (embryonic day 15.5) and P0 (postnatal day 0) testes. White arrow indicates fetal pro-spermatogononia with shown in zoom in panel; Arrow head indicates neonatal pro-spermatogonia with shown in zoom in panel. (**c**) Co-immunofluorescence using anti-UHRF1 and anti-γ-H2A.X antibodies on WT germ cells from testis sections of the first wave of spermatogenesis are shown. The postnatal day (P) of the testis section is indicated in each panel. Nuclei were stained with DAPI. Abbreviations: B, type B spermatogonia; In, Intermediate spermatogonia; Pl, pre-leptotene; L, leptotene; Z, zygotene; P, pachytene; D, diplotene; M, meiotic metaphase; RS, round spermatid; ES, elongating spermatid and Sg, Spermatogonia. (**d**) Immunofluorescent staining with anti-UHRF1 and anti-SYCP3 on surface-spread spermatogonia and spermatocytes from control mice are shown.

Supplementary Figure 3

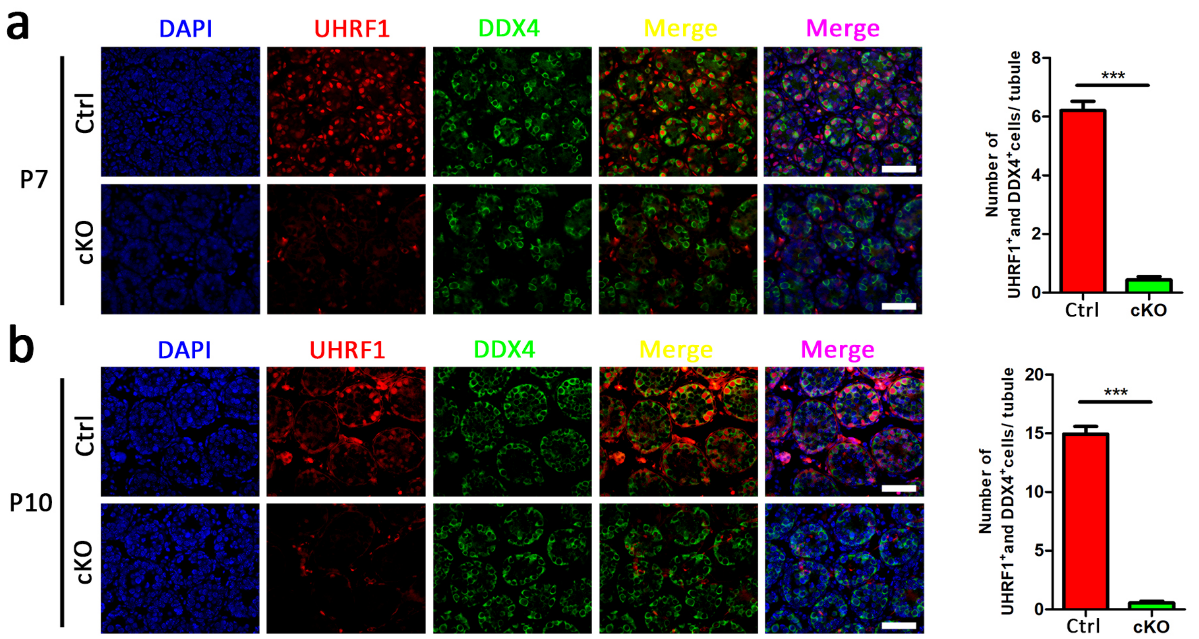

Supplementary Fig.3. *Stra8*-Cre mediated germ cell depletion efficiency was examined by immunofluorescent staining. Co-immunofluorescent staining using anit-UHRF1 and anti-DDX4 (a germ cell marker) on control and *Uhrf1* cKO testis sections at P7 (**a**) and P10 (**b**) are shown. Nuclei were stained with DAPI. The right histograms showed the quantifications of UHRF1$^+$ and DDX4$^+$ cells per tubule at P7 and P10 testes in control and *Uhrf1* cKO mice. Mean ± SEM, n = 5. ****P*<0.001 by Student's *t*-test. Scale bar=50μm.

Supplementary Figure 4

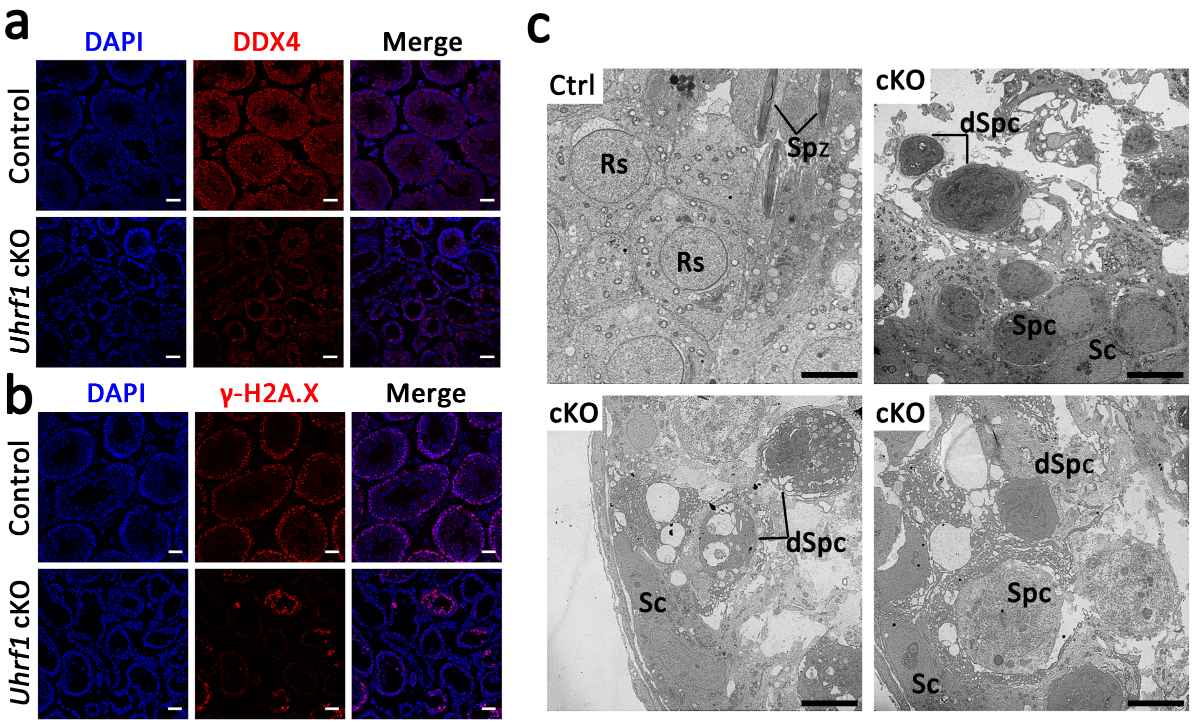

Supplementary Fig.4. Conditional inactivation of *Uhrf1* causes germ cells depletion in adult testes. (**a**) Immunofluorescence staining with DDX4 (a germ cell marker) in control and *Uhrf1* cKO adult testes are shown. Scale bar=50μm**.** (**b**) Immunofluorescence staining with γ-H2A.X in control and *Uhrf1* cKO adult testes is shown. Scale bar=50μm. (**c**) Transmission electron microscopy (TEM) revealing massive degenerated germ cells displayed in *Uhrf1* cKO adult testes. Abbreviations: Sc, Sertoli cell; Spc, Spermatocytes; dSpc, degenerated spermatocytes; Rs, Round spermatids; Spz, Spermatozoa. Scale bar=10μm.

Supplementary Figure 5

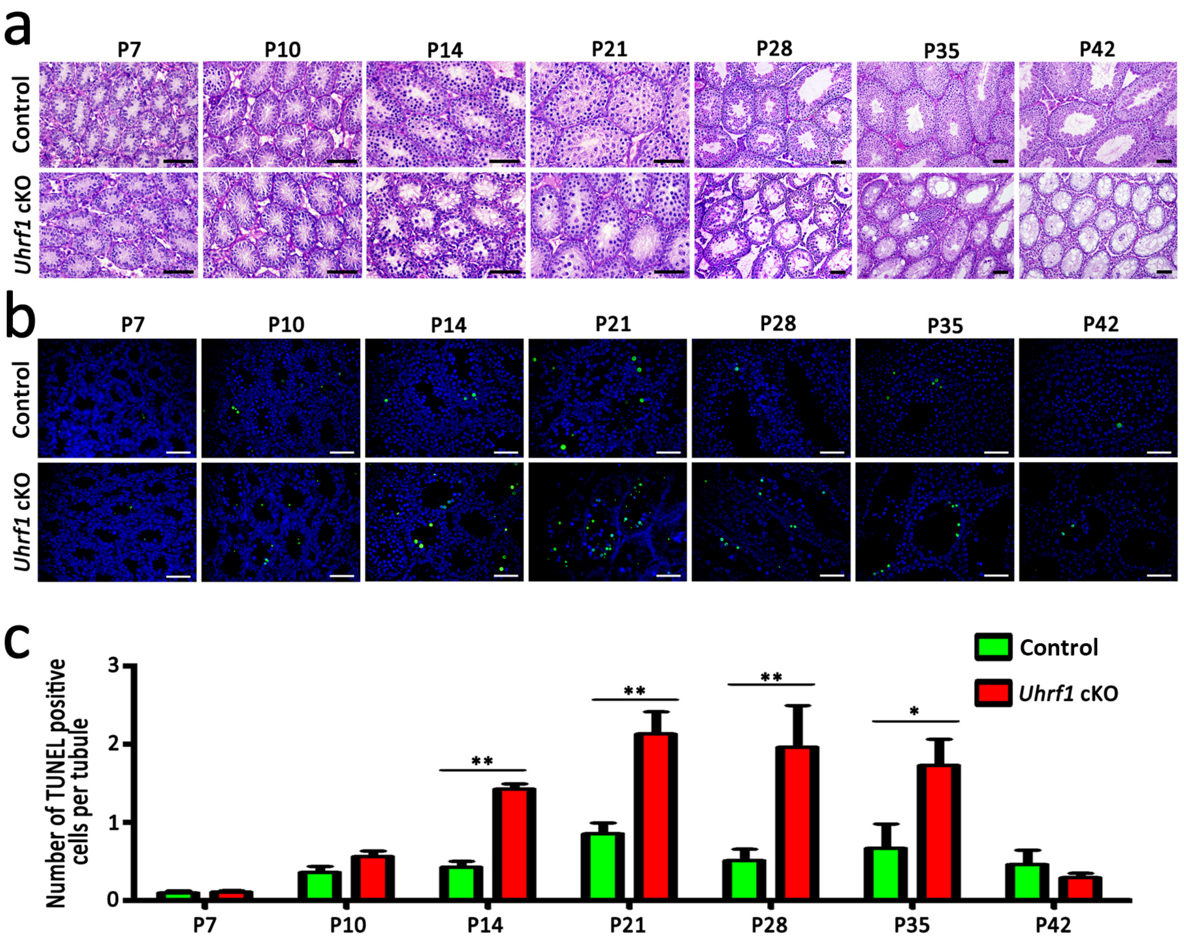

Supplementary Fig.5. Histological and TUNEL analyses on developing testes of control and *Uhrf1* cKO mice. (**a**) Representative PAS-stained paraffin sections of developing testes at postnatal 7 (P7), P10, P14, P21, P28, P35 and P42 from control and *Uhrf1* cKO mice. Note that the morphology and total number of germ cells are distinguishable between control and *Uhrf1* cKO testes started from P14. Scale bar=100μm. (**b**) Representative results of TUNEL assays on paraffin sections of control and *Uhrf1* cKO testes at P7, P10, P14, P21, P28, P35 and P42. Scale bars=100μm. (**c**) Quantification of apoptotic cells in control and *Uhrf1* cKO developing testes at P7, P10, P14, P21, P28, P35 and P42. Data are presented as mean ± SEM, n = 5. *P<0.05 and **P<0.01 (Student's t-test).

Supplementary Figure 6

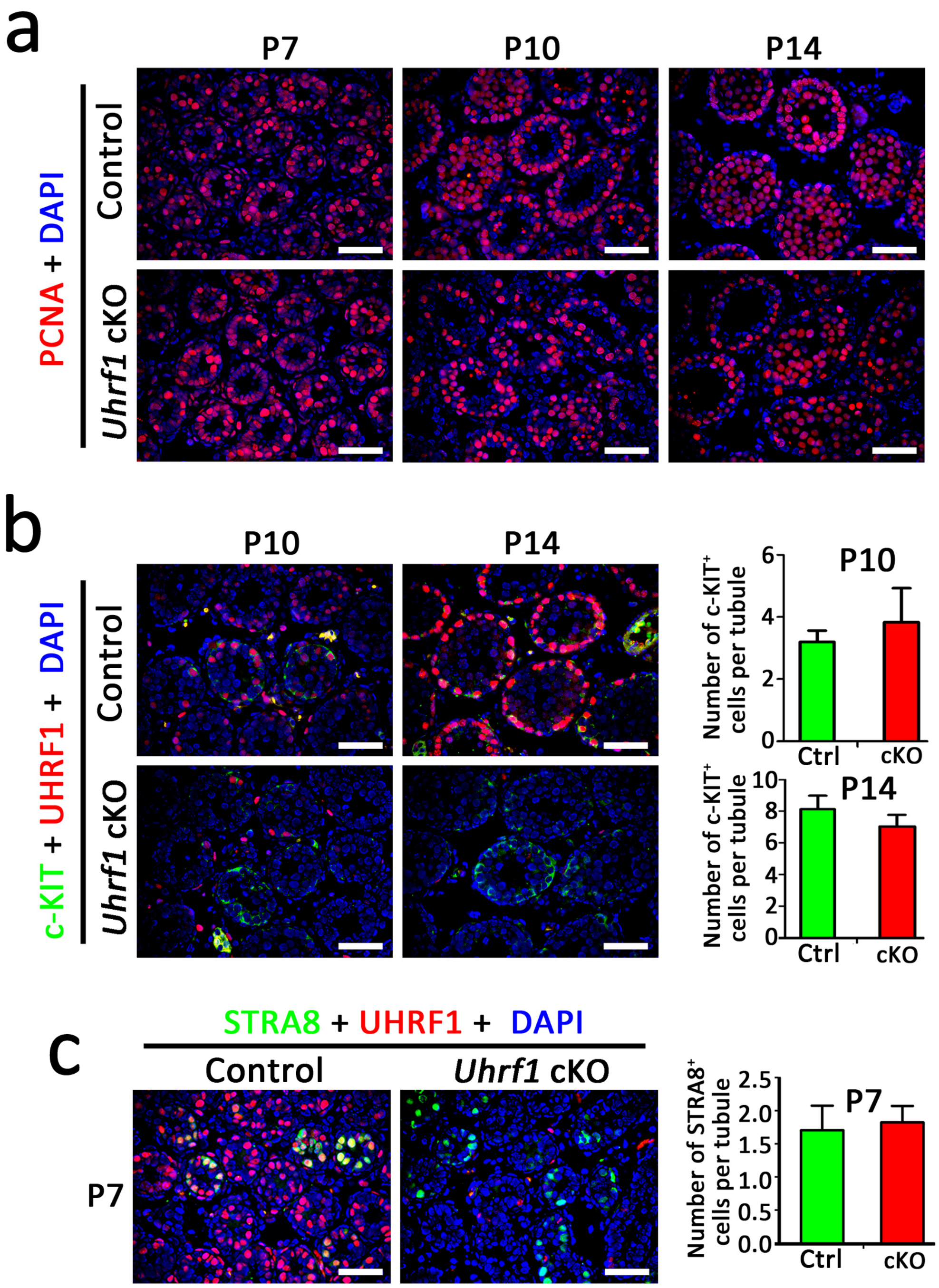

Supplementary Fig.6. Loss of *Uhrf1* did not affect spermatogonial differentiation and meiotic initiation. (**a**) Immunofluorescent staining with PCNA on control and *Uhrf1* cKO mouse testes at P7, P10 and P14 are shown. Nuclei were stained with DAPI. Scale bar=100μm. (**b**) Co-immunofluorescent staining with c-KIT and UHRF1 in control and *Uhrf1* cKO mouse testes at P10, and P14 are shown. Nuclei were stained with DAPI. The right histograms show that the quantification of c-KIT$^+$ cells per tubule at P10, and P14 in control and *Uhrf1* cKO testes. Data are presented as mean ± SEM, n = 5. Scale bar=100μm. (**c**) Co-immunofluorescent staining for STRA8 and UHRF1 antibodies on control and *Uhrf1* cKO testis sections at P7 is shown. Nuclei were stained with DAPI. The right histograms show that the quantifications of STRA8$^+$ cells per tubule at P7 in control and *Uhrf1* cKO testes. Data are presented as mean ± SEM, n = 5. Scale bar bar=100μm.

Supplementary Figure 7

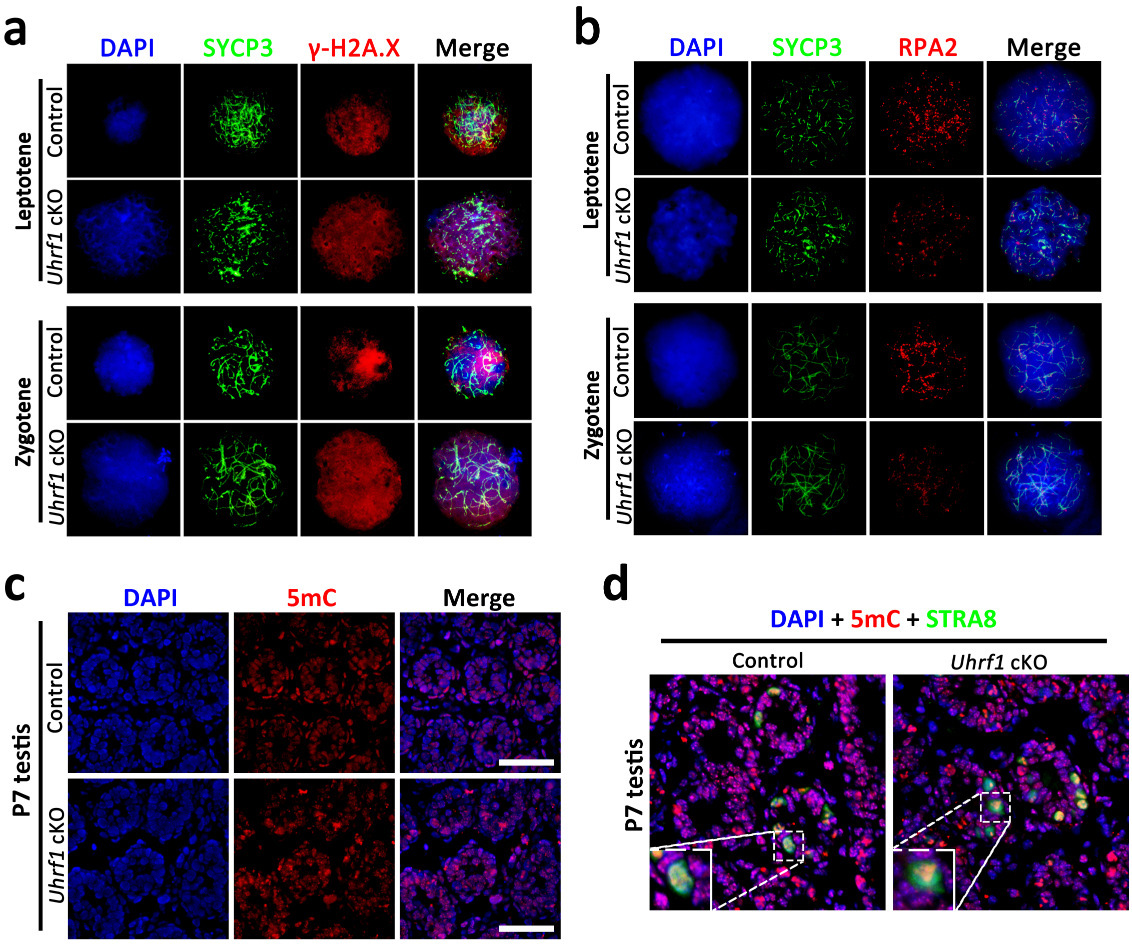

Supplementary Fig.7. Conditional deletion of *Uhrf1* in testes causes meiotic defects, but not affect differentiated spermatogonia methylation status. (**a**) Immunofluorescent staining with anti-SYCP3 and anti-γ-H2A.X on surface-spread leptotene and zygotene spermatocytes from control and *Uhrf1* cKO mice are shown. (**b**) Immunofluorescent staining with anti-SYCP3 and anti-RPA2 on surface-spread leptotene and zygotene spermatocytes from control and *Uhrf1* cKO mice are shown. (**c**) Immunofluorescent staining with 5mC on control and *Uhrf1* cKO mouse testes at P7. Nuclei were stained with DAPI. Scale bar bar=100μm. (**d**) Co-immunofluorescent staining for STRA8 and 5mC antibodies on control and *Uhrf1* cKO testis sections at P7 is shown. Zoom in panel shown differentiated spermatogonia with STRA8 positive cell. Nuclei were stained with DAPI.

Supplementary Figure 8

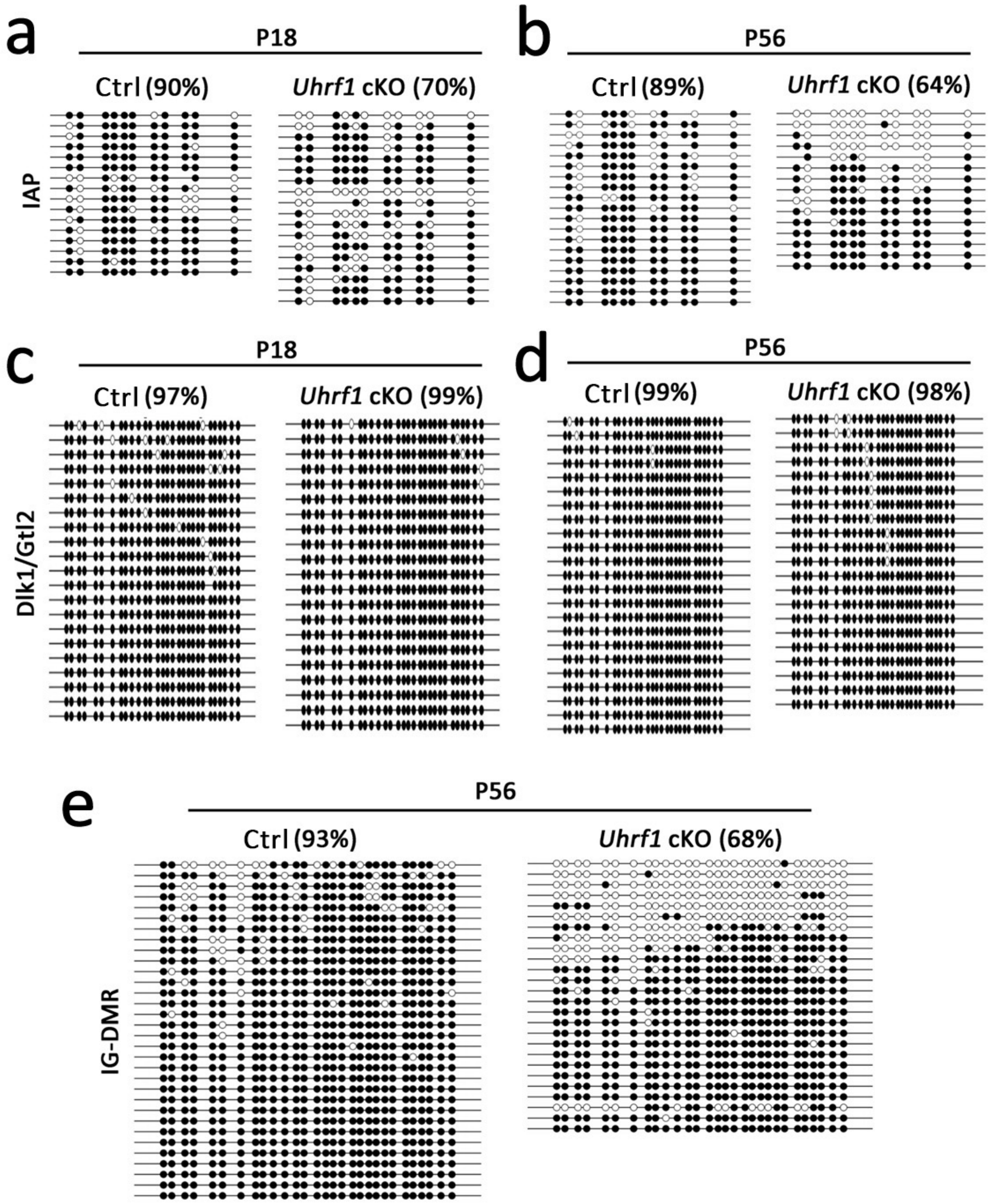

Supplementary Fig.8. Bisulfate sequencing of genomic DNA from control and *Uhrf1* cKO testes. (**a-e**) Genomic DNA from control and *Uhrf1* cKO testes at P18 and P56 were subjected to bisulfate sequencing of retrotransposons IAP (**a-b**), parent-of origin specific imprinted gene Dlk1/Gtl2 (**c-d**) and IG-DMR (**e**). Methylated CpG sites are shown with black circles and unmethylated sites with open circles. The percentages of overall methylated CpGs are indicated.

Supplementary Figure 9

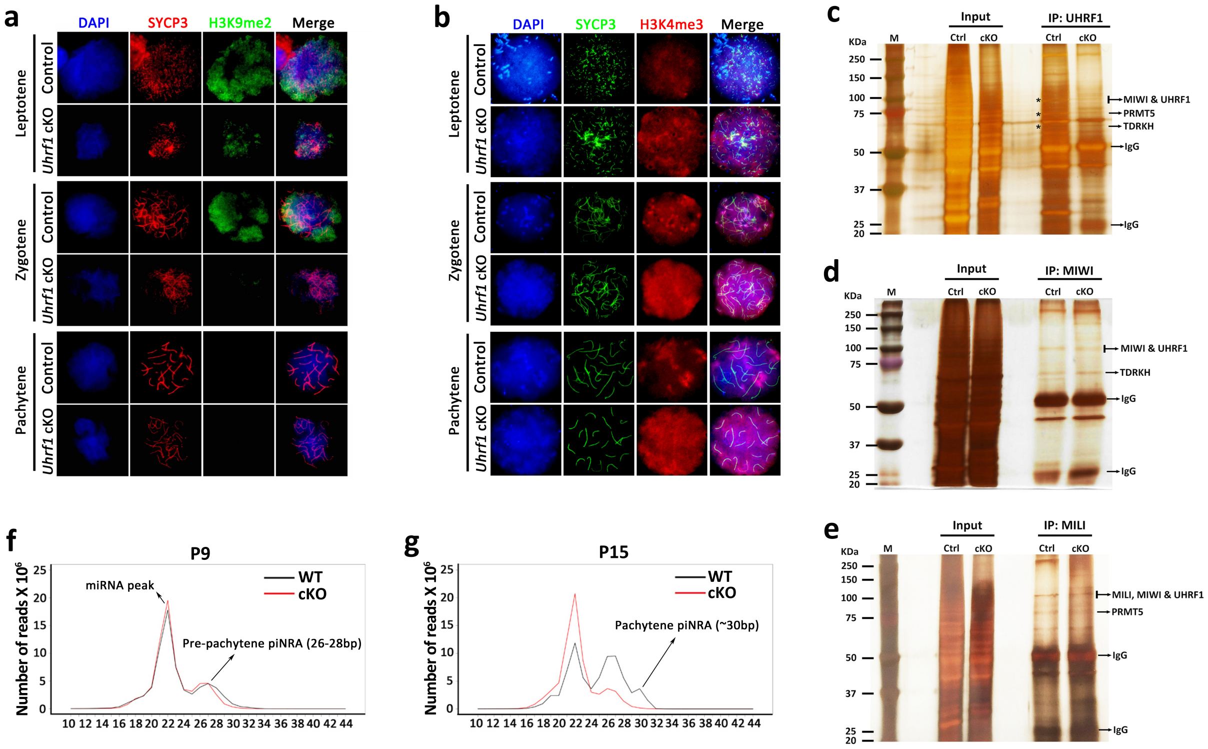

Supplementary Fig.9. Histone modification and pachytene piRNA biogenesis were compromised in *Uhrf1* cKO mice. (**a**) Co-immunofluorescent staining with H3K9me2 and SYCP3 on surface-spread spermatocytes from control and *Uhrf1* cKO mouse testes. (**b**) Co-immunofluorescent staining with H3K4me3 and SYCP3 on surface-spread spermatocytes from control and *Uhrf1* cKO mouse testes. (**c-e**) Silver staining showing the protein distribution in gels responding to Fig.5d-f, respectively. (**f-g**) Size distribution of small RNA libraries from control and *Uhrf1* cKO testes at P9 (**c**) and P15 (**d**) without normalization.

Supplementary Figure 10

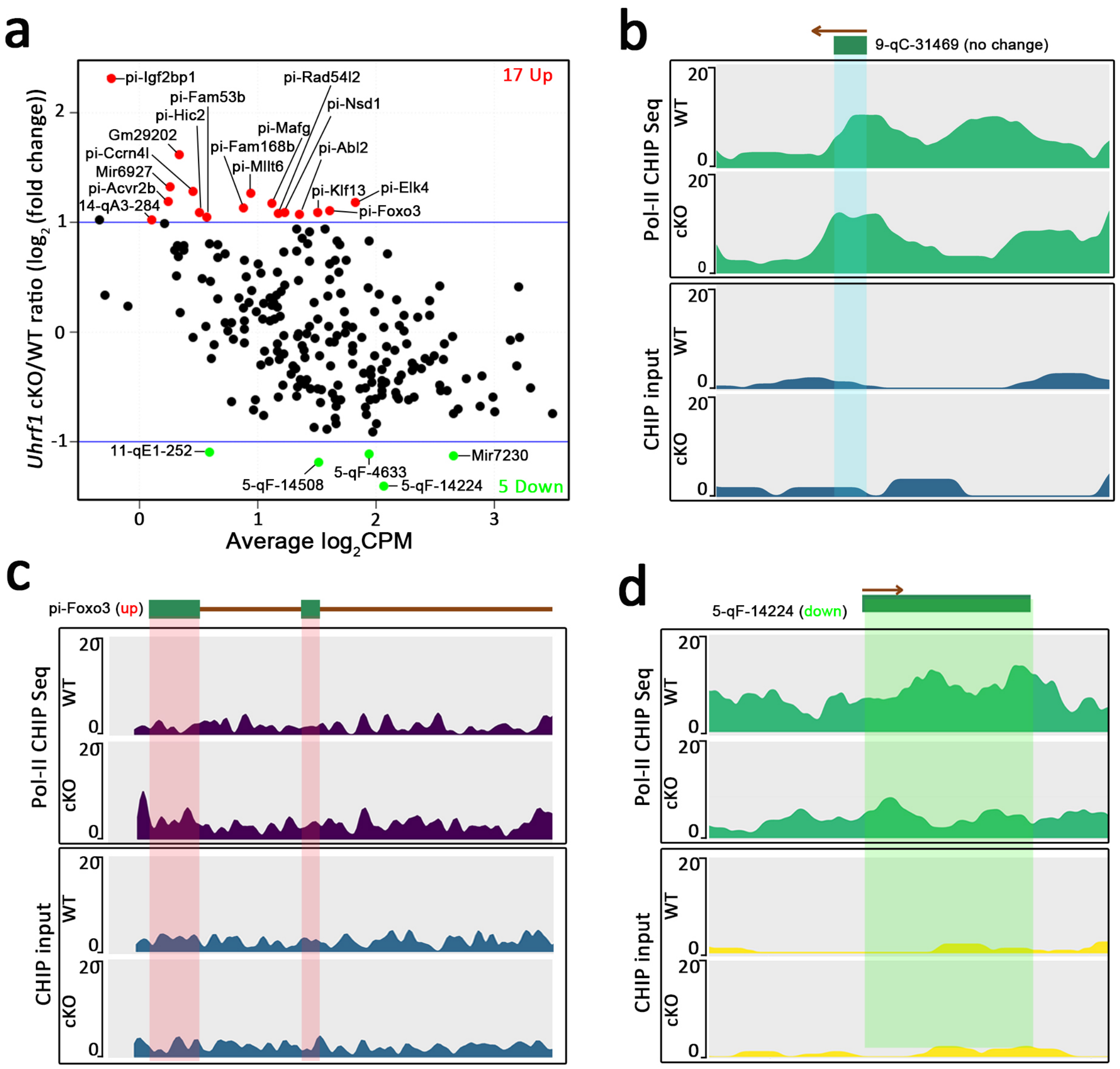

Supplementary Fig.10. RNA Pol-II CHIP-Seq revealed the piRNA transcription changes between *Uhrf1* cKO and WT testes. (**a**) MA plot comparing occupancy in RNA pol-II of 214 piRNA clusters between *Uhrf1* cKO and WT testes at P18. The Y-axis represents the $\log_2$ (fold change, FC), whereas the X-axis represents the average $\log_2$ (counts per million reads, CPM). Values highlighted in color showing the up- (red) and down-regulated (green) piRNA clusters with an adjusted p-value of less than 0.05. The number of up- and down-regulated piRNA clusters enriched in *Uhrf1* cKO is indicated. (**b-d**) Genome browser panels showing read coverage at representative piRNA clusters including 9-qC-31469 (no change) (**b**), pi-Foxo3 (up-regulated) (**c**) and 5-qF-14224 (down-regulated) (**d**) from Pol-II CHIP-seq and CHIP-seq input samples of the indicated genotypes. The red arrow indicates the transcriptional direction.

Supplementary Figure 11

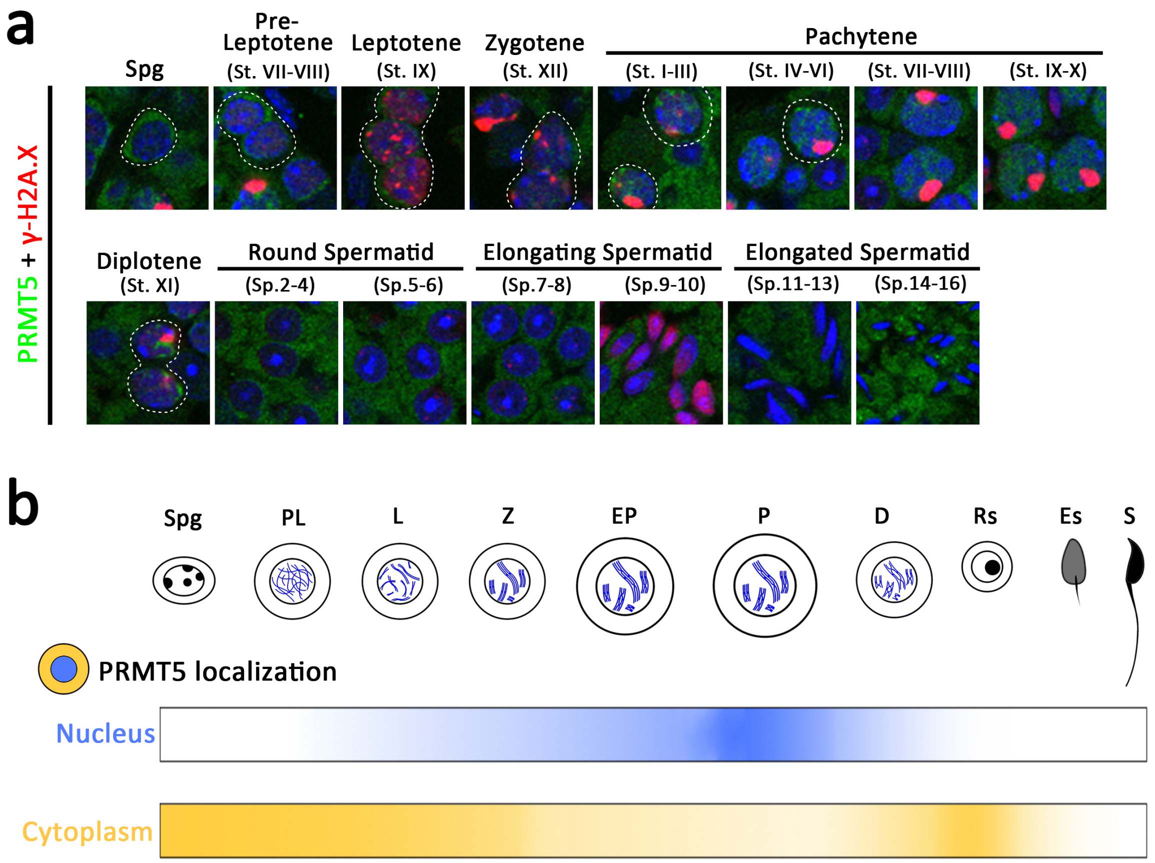

Supplementary Fig.11. PRMT5 display a dynamic expression profile during adult spermatogenesis. (**a**) Double immunostaining with PRMT5 and γ-H2A.X on WT germ cells from adult testis sections are shown. Dashed lines outline the indicated cell types. (**b**) A schematic summary of the dynamic localizations of PRMT5 in adult testis during spermatogenesis. Spg, Spermatogonia; PL, Pre-leptotene; L, Leptotene; Z, Zygotene; EP, early pachytene; P, Pachytene; D, Diplotene; Rs, Round spermatids; Es, Elongating spermatids; S, Spermatozoa.

Supplementary Figure 12

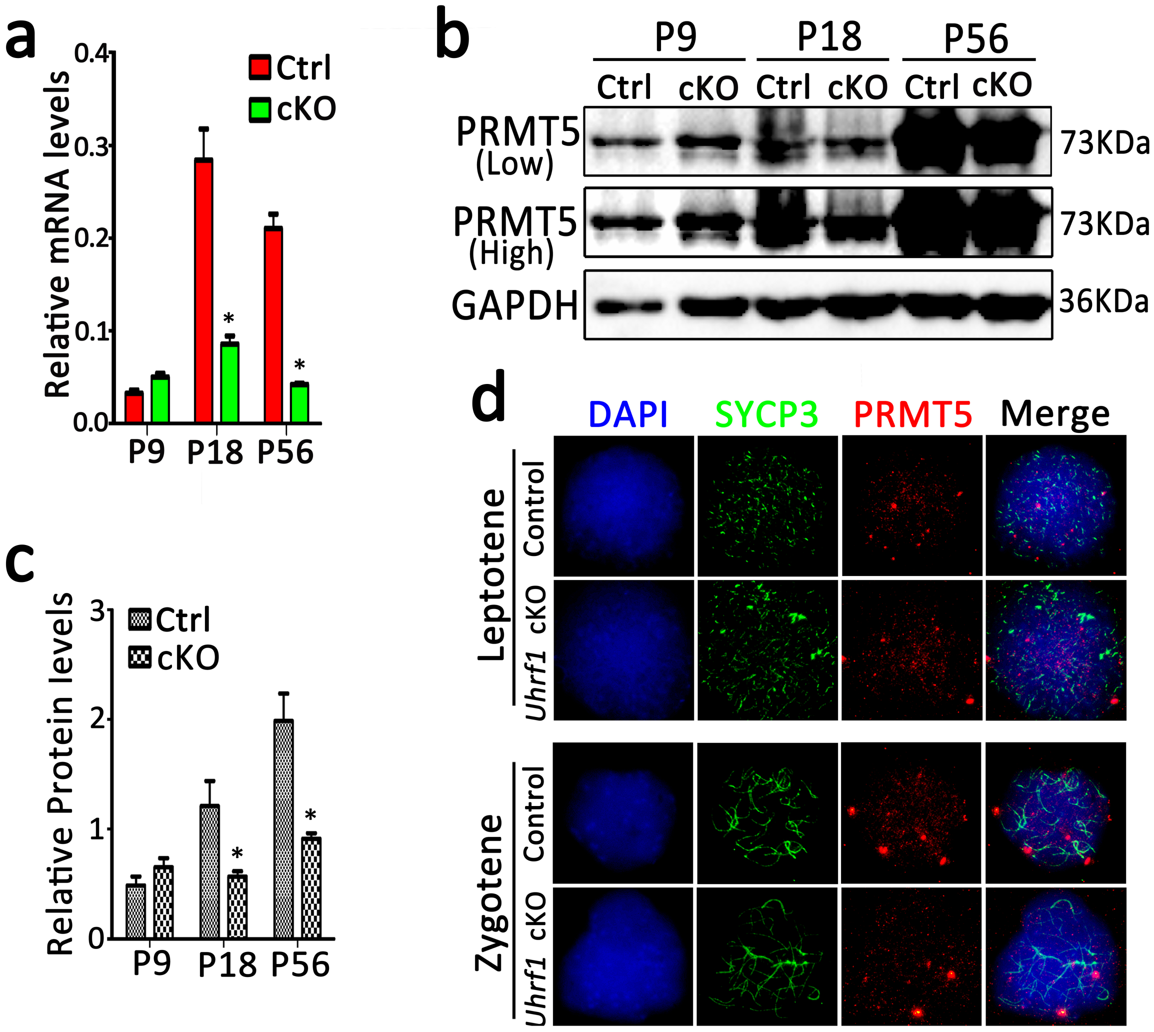

Supplementary Fig.12. UHRF1 colocalize with PRMT5 and could affect PRMT5 expression levels. (**a**) RT-qPCR analysis showing *Prmt5* mRNA levels in control and *Uhrf1* cKO testes at P9, P18 and P56. **P*<0.05 by Student's *t*-test. Data are presented as mean ± SEM, n = 5. (**b**) Western blot detecting PRMT5 protein levels in control and *Uhrf1* cKO testes at P9, P18 and P56. GAPDH served as a loading control. (**c**) Histogram showing the quantification of PRMT5 protein levels based on the Western blot data. **P*<0.05 by Student's *t*-test. Data are presented as mean ± SEM, n = 3. (**d**) Co-Immunofluorescent staining with PRMT5 and SYCP3 of surface-spread leptotene and zygotene spermatocytes from control and *Uhrf1* cKO testes.

Supplementary Figure 13

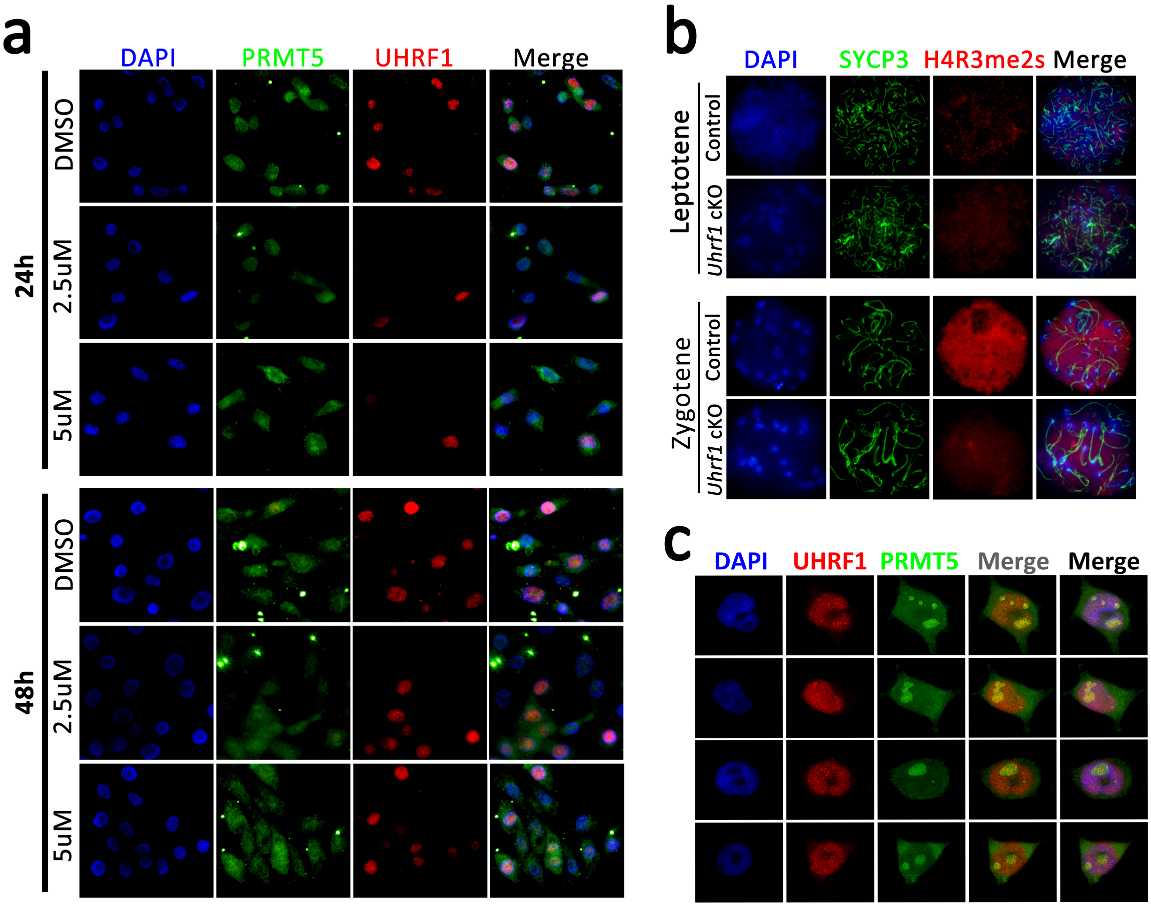

Supplementary Fig.13. Loss of UHRF1 affects PRMT5 expression and histone H4R3me2s modification. (**a**) Co-immunostaining of PRMT5 and UHRF1 in NIH3T3 cells with treatment of GSK591 (an inhibitor of PRMT5) in different concentration for 24 and 48 hours. DNA was stained with DAPI. (**b**) Co-Immunofluorescent staining with H4R3me2s and SYCP3 of surface-spread leptotene and zygotene spermatocytes from control and *Uhrf1* cKO testes. (**c**) Co-Immunofluorescent staining with UHRF1 and PRMT5 in HEK293T cells.

Supplementary Figure 14

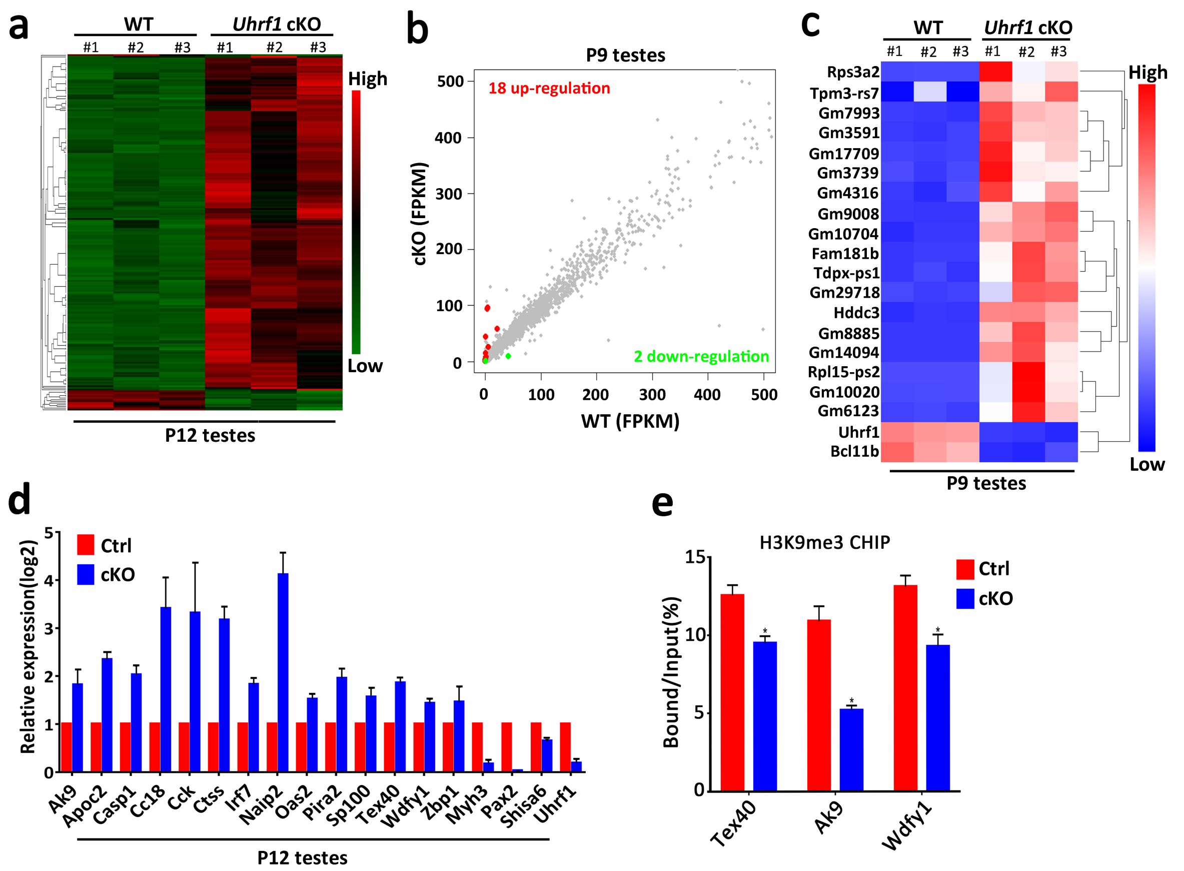

Supplementary Fig.14. Global transcriptional dys-regulation occurs in *Uhrf1* cKO testes at P12, but not at P9. (**a**) Heat map showing 213 deregulated genes at P12 in the *Uhrf1* cKO testes, which including 200 genes upregulated and 13 genes downregulated. Significantly regulated genes have a *P*-value of <0.05 and fold change of > 2.0. Three biological replicates were indicated in the heat-map. (**b**) Graph plot showing only 20 genes were dys-regulated in *Uhrf1* cKO testes at P9. Significantly regulated genes have a *P*-value of <0.05 and fold change of > 2. Three biological replicates were indicated. (**c**) Heat map showing 20 deregulated genes at P9 in the *Uhrf1* cKO testes, which including 18 genes upregulated and 2 genes downregulated. (**d**) RT-qPCR analyses of 18 significantly genes to validate RNA-Seq from P12 testes. (**e**) ChIP-qPCR showing the H3K9me3 enrichments at three selected gene promoter regions in control and *Uhrf1* cKO mouse testes from RNA-Seq data of P12 testes. Quantitative data are expressed as the ratio of the ChIP (Bound) to the input DNA. Error bars indicate the SEM of three biological replicates. *$P < 0.05$ by Student's *t*-test.

Supplementary Figure 15

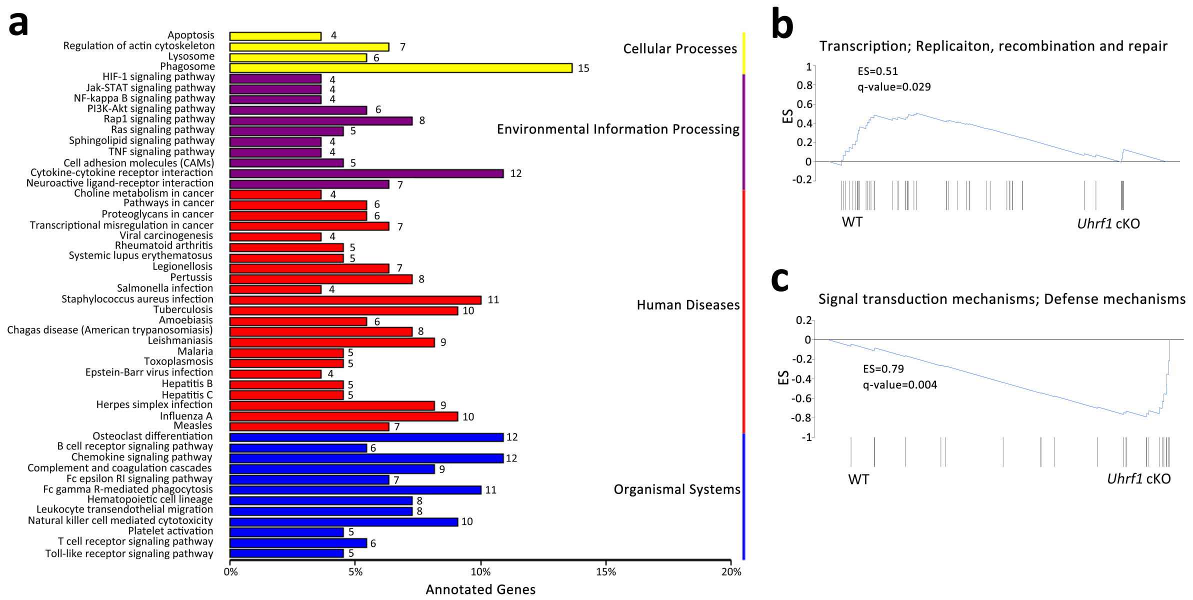

Supplementary Fig.15. (**a**) KEGG analysis of 213 deregulated genes in the *Uhrf1* cKO testes at P12 showing most of deregulated genes are related to human diseases and environmental information processing. (**b-c**) Graph showing gene set enrichment analysis (GSEA) of RNA-Seq at P12 testes data. *Uhrf1* cKO testes show underrepresentation of genes involved in DNA replication, recombination and repair (**b**), whereas overrepresentation of genes related to defense mechanisms (**c**). False discovery rate (FDR), <25%.

Supplementary Figure 16

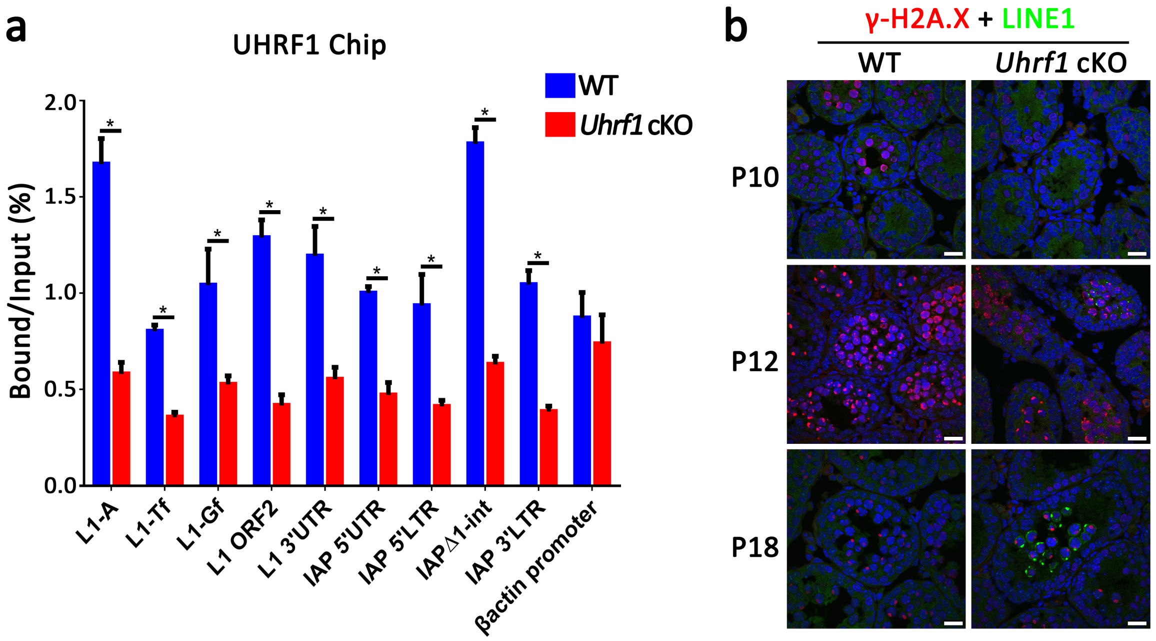

Supplementary Fig.16. UHRF1 binding to retrotransposon elements in testes. (**a**) ChIP-qPCR showing the UHRF1 enrichments at various retrotransposons in control and *Uhrf1* cKO mouse testes at P21. Quantitative data are expressed as the ratio of the ChIP (Bound) to the input DNA. The β-actin promoter was used as a negative control for UHRF1 enrichment. Error bars indicate the SEM of three biological replicates. *$P < 0.05$ by Student's *t*-test. (**b**) Co-immunofluorescent staining with anti-LINE1 ORF1 and anti-γ-H2A.X in P10, P12 and P18 testis sections from control and *Uhrf1* cKO mice are shown. Scale bar=20μm.

Supplementary Figure 17

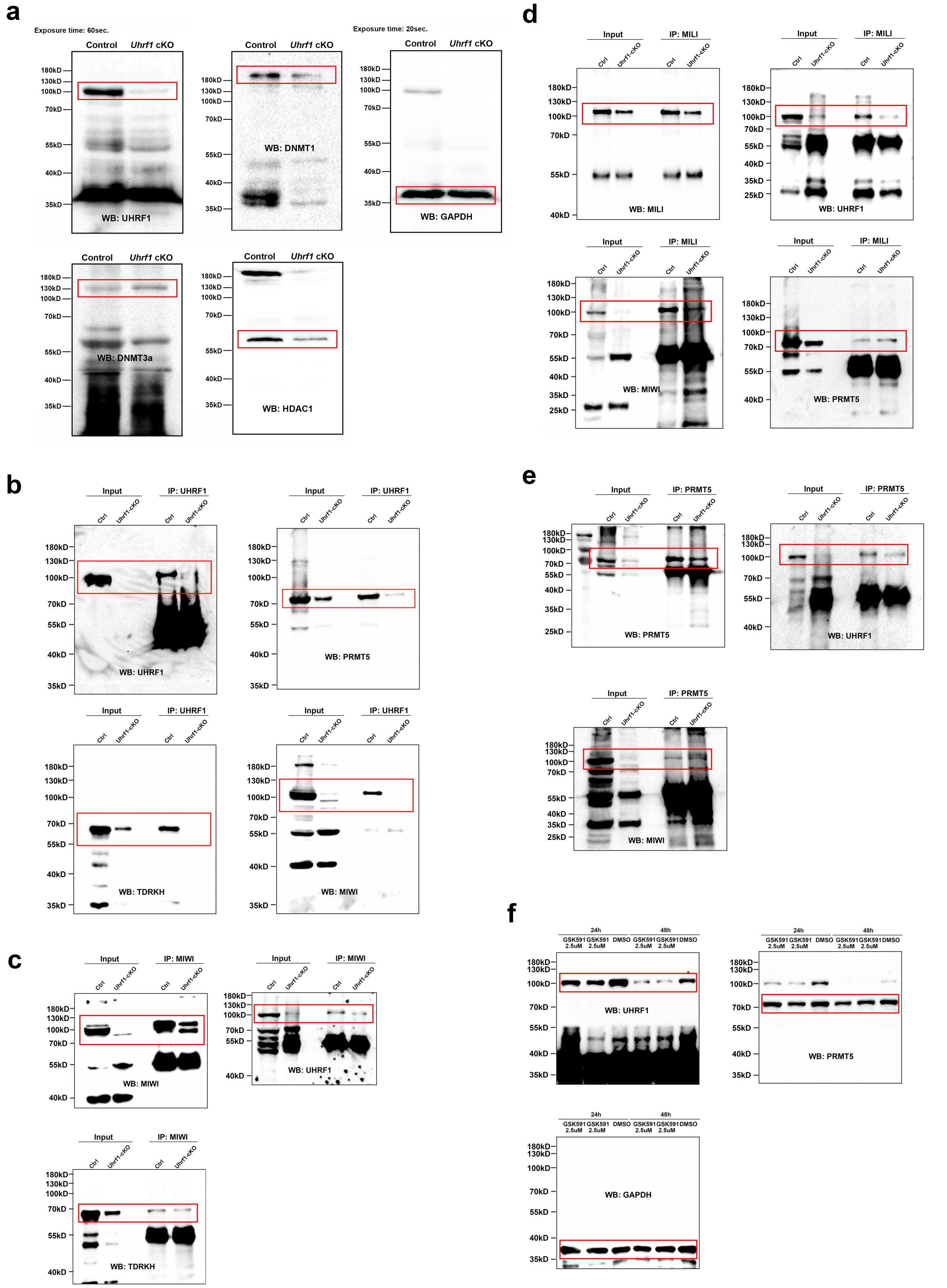

Supplementary Fig.17. Original un-cropped Western blots shown in the manuscript. (**a**) Western blots correspond to Figure 2d. (**b**) Western blots correspond to Figure 5d. (**c**) Western blots correspond to Figure 5e. (**d**) Western blots correspond to Figure 5f. (**e**) Western blots correspond to Figure 6b. (**f**) Western blots correspond to Figure 6e.

Supplementary Figure 18

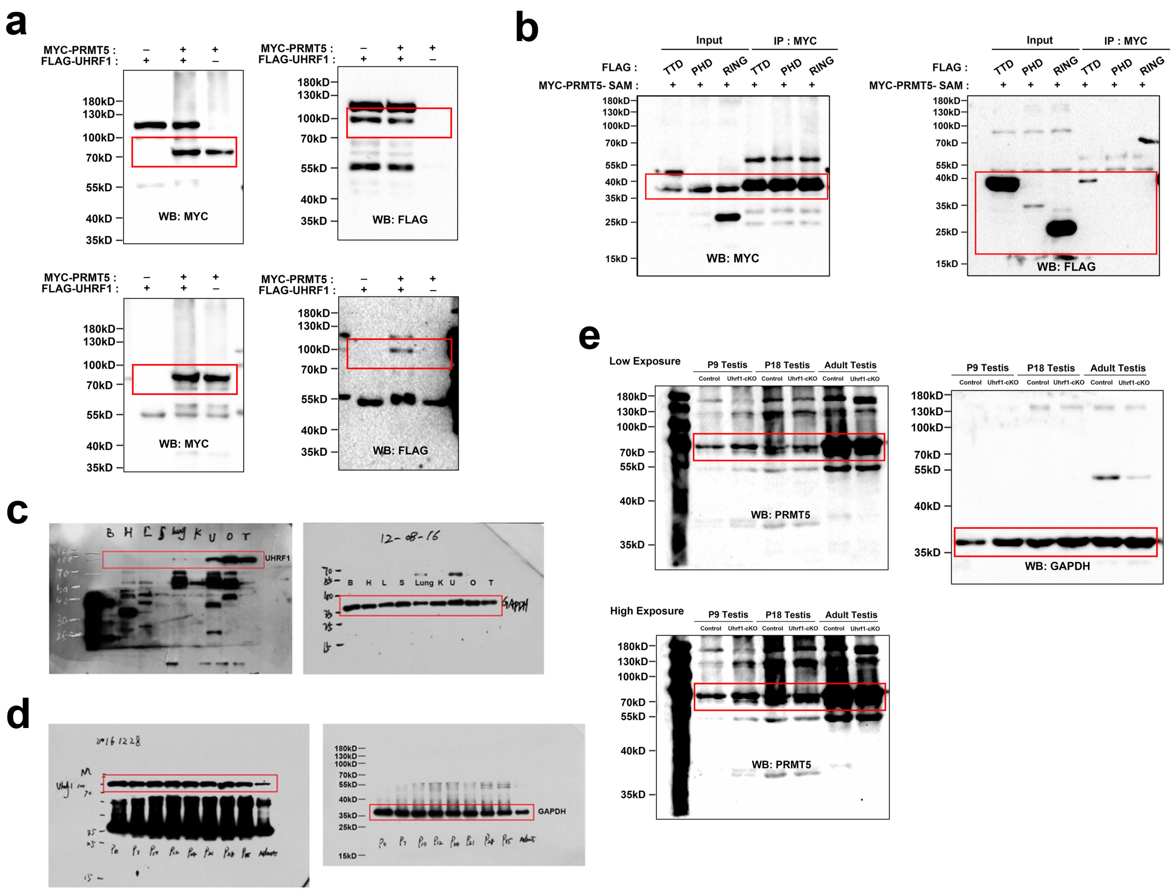

Supplementary Fig.18. Original un-cropped Western blots shown in the manuscript. (**a**) Western blots correspond to Figure 7b. (**b**) Western blots correspond to Figure 7c. (**c**) Western blots correspond to Supplementary Figure 1d. (**d**) Western blots correspond to Supplementary Figure 1f. (**e**) Western blots correspond to Supplementary Figure 12b.

Supplementary Table 1. Antibodies used in this study.

Antibodies	Species	Concentration	Company source	Cat #
UHRF1	Mouse	1:100	santa	sc-373750
DDX4/MVH	Rabbit	1:400	abcam	ab13840
WT1	Rabbit	1:200	abcam	ab89901
SYCP3	Rabbit	1:200	abcam	ab15093
SYCP3	Mouse	1:100	santa	sc-74569
rH2AX	Mouse	1:200	abcam	ab26350
rH2AX	Rabbit	1:200	abcam	ab11174
c-KIT	Rabbit	1:100	Cell signaling technology	3074
PLZF	Mouse	1:100	santa	SC-28391
Pol-II	Rabbit	1:500	abcam	ab817
H3K9me2	Mouse	1:500	abcam	ab1220
H3K9me3	Rabbit	1:500	abcam	ab8898
H3K4me3	Rabbit	1:500	abcam	ab8580
H4R3me2s	Rabbit	1:200	Epigentek	A-3718
H3R2me2s	Rabbit	1:200	Epigentek	A-3705
5mc	Mouse	1:100	Epigentek	A-1014
PIWIL1/MIWI	Rabbit	1:100	Proteintech	15659-1-AP
PIWIL2/MILI	Rabbit	1:100	MBL	PM044
STRA8	Rabbit	1:200	Milipore	ABN1656
TDRKH	Rabbit	1:100	Proteintech	13528-1-AP
PCNA	Mouse	1:100	santa	sc-56
DNMT1	Mouse	1:1000	santa	sc-271729
DNMT3A	Mouse	1:1000	santa	sc-271729
HDAC1	Mouse	1:1000	santa	sc-81598
GAPDH	Rabbit	1:4000	Proteintech	10494-1-AP
NP95	Rat	1:1000	MBL	D289-3
MYC tag	Mouse	1:1000	Proteintech	60003-2-Ig
HA tag	Mouse	1:1000	Abbkine	A02040
RPA2	Rabbit	1:500	Gift	
Line1 ORF1	Rabbit	1:500	Home made	
Line1 ORF1	Rabbit	1:1000	Gift from Prof. Ramesh Pillai (University of Geneva, Switzerland)	
Goat anti-rat IgG	Goat	1:10000	Biosharp	BL002A
Goat anti-rat IgG	Goat	1:10000	Thermo Fisher	62-9520
HRP Goat anti-mouse IgG	Goat	1:10000	Abbkine	A21010-1
HRP Goat anti-rabbit IgG	Goat	1:10000	Abbkine	A21020-1
Dylight 488 Goat anti-mouse IgG	Goat	1:500	Abbkine	A23210
Dylight 594 Goat anti-rabbit IgG	Goat	1:500	Abbkine	A23420
Alexa FluorTM 488 Goat anti-mouse	Goat	1:500	Invitrogen	A11029

IgG (H+L)				
Alexa FluorTM 488 Goat anti-rabbit IgG (H+L)	Goat	1:500	Invitrogen	A32731
Alexa FluorTM 594 Goat anti-rabbit IgG (H+L)	Goat	1:500	Invitrogen	A11032
Cy3-conjugated Affinipure Goat anti-mouse IgG (H+L)	Goat	1:250	Proteintech	SA00009-1

Supplementary Table 2. Primer sequences are used in this study.

Target	Sequence (5’ to 3’)	Application
Stra8-cre	F: GTGCAAGCTGAACAACAGGA	Genotyping
	R: AGGGACACAGCATTGGAGTC	
Uhrf1-loxp	F: TTGCTGCCAGGTAGGACAT	Genotyping
	R: GAAGAGGCGGCTCAGGAGA	
Gapdh	F: AGGTCGGTGTGAACGGATTTG	RT-qPCR
	R: GGGGTCGTTGATGGCAACA	
Uhrf1	F:CTAGCAGCTGGAAGGAACCC	RT-qPCR
	R:GATGTACTCTCTCACGGCGG	
Dnmt1	F:TCTGTCAGCAGCCTGAGTGT	RT-qPCR
	R:CTCCTTCACCGCCAAGTTAG	
Dnmt3a	F:GAGGGAACTGAGACCCCAC	RT-qPCR
	R:CTGGAAGGTGAGTCTTGGCA	
Dnmt3b	F:CTCACACCTGAGCTGTACTGCAGAG	RT-qPCR
	R:CTCCACCTTCTGAGACTCTCCAGAG	
Hdac1	F:TGTGTCCTTTCATAAATACGG	RT-qPCR
	R:TCTCGCAGTGGGTAGTTC	
Minorsatellite	F:CATGGAAAATGATAAAAACC	RT-qPCR
	R:CATCTAATATGTTCTACAGTGTGG	
MusD	F:GATTGGTGGAAGTTTAGCTAGCAT	RT-qPCR
	R:TAGCATTCTCATAAGCCAATTGCA T	
Dlk1	F:ACTTGCGTGGACCTGGAGAA	RT-qPCR
	R:CTGTTGGTTGCGGCTACGAT	
Mirg	F:TTGACTCCAGAAGATGCTCC	RT-qPCR
	R:CCTCAGGTTCCTAAGCAAGG	
Gtl2	F:TTGCACATTTCCTGTGGGAC	RT-qPCR
	R:AAGCACCATGAGCCACTAGG	
Dnmt1	F:TCTGTCAGCAGCCTGAGTGT	RT-qPCR
	R:CTCCTTCACCGCCAAGTTAG	
Dnmt3a	F:GAGGGAACTGAGACCCCAC	RT-qPCR
	R:CTGGAAGGTGAGTCTTGGCA	
Dnmt3b	F:CTCACACCTGAGCTGTACTGCAGAG	RT-qPCR
	R:CTCCACCTTCTGAGACTCTCCAGAG	
Prmt5	F: CTGAATTGCGTCCCCGAAATA	RT-qPCR
	R:AGGTTCCTGAATGAACTCCCT	
Line1 5'UTR	F:GGCGAAAGGCAAACGTAAGA	RT-qPCR
	R:GGAGTGCTGCGTTCTGATGA	
IAP Gag	F:AACCAATGCTAATTTCACCTTGGT	RT-qPCR
	R:GCCAATCAGCAGGCGTTAGT	
L1 ORF2	F : GGAGGGACACTTCATTCTCATCA	ChIP-qPCR/RT-qPCR
	R : GCTGCTCTTGTATTTGGAGCATAGA	
L1 3’ UTR	F : ATGGACCATGTAGAGACTGCCA	ChIP-qPCR
	R : CAATGGTGTCAGCGTTTGGA	
L1 Tf	F : CAGCGGTCGCCATCTTG	ChIP-qPCR/RT-qPCR
	R : CACCCTCTCACCTGTTCAGACTAA	

L1 A	F : GGATTCCACACGTGATCCTAA	ChIP-qPCR/RT-qPCR
	R : TCCTCTATGAGCAGACCTGGA	
L1 Gf	F : CTCCTTGGCTCCGGGACT	ChIP-qPCR
	R : CAGGAAGGTGGCCGGTTGT	
IAP 5’ UTR	F : CGGGTCGCGGTAATAAAGGT	ChIP-qPCR
	R : ACTCTCGTTCCCCAGCTGAA	
IAP Δ1-int	F : AACGCTGCTGCTTTAACTCC	ChIP-qPCR/RT-qPCR
	R :TGCACATAAAGCTGGCACA	
IAP LTR	F : CTCCATGTGCTCTGCCTTCC	ChIP-qPCR/RT-qPCR
	R : CCCCGTCCCTTTTTTAGGAGA	
IAP 3’ LTR	F : GCACATGCGCAGATTATTTGTT	ChIP-qPCR/RT-qPCR
	R : CCACATTCGCCGTTACAAGAT	
Mest	F: CAGCAGCTTCTGGCATGTGG	ChIP-qPCR
	R: AACCCCAGATTCTAGTGAAG	
Gapdh promoter	F: TTGCTTAGGCCTTCCTTCTTC	ChIP-qPCR
	R: CATCACCTGGCCTACAGGATA	
β-actin promoter	F : CACCCATCGCCAAAACTCTTCATCCT	ChIP-qPCR
	R : CGCACAGTGCAGCATTTTTTTACC	
Line1 5'	F:CACCGTTAGAGAATTTGATAGTTTTTGGAATAGG	BSP
	R:TTACCAAAACAAAACCTTTCTCAAACACTATAT	
IAP LTR	F:CACCTTGTGTTTTAAGTGGTAAATAAATAATTTG	BSP
	R:TTACAAAAAAAACACACAAACCAAAAT	
Dlk1/Gtl2	F:GGTTTGGTATATATGGATGTATTGTAATATAGG	BSP
	R:ATAAAACACCAAATCTATACCAAAATATACC	
IG-DMR	F:TAAGTGTTGTGGTTTGTTATGGGTA	BSP
	R:CCATCCCCTATACTCAAAACATTCT	
	R:CTGGCTTCTCTGTGGCTCTC	
Ak9	F: AACCTGTCTCCTGGATGCCT	RNA-seq/RT-qPCR
	R: TCAGACCGTTAGCCTCACAC	
Apoc2	F: AGGCAAAGTCTTAGAGGCCG	RNA-seq/RT-qPCR
	R: CCAGTACTCCAGTGGGTTGG	
Casp1	F: ACTGACTGGGACCCTCAAGT	RNA-seq/RT-qPCR
	R: GCAAGACGTGTACGAGTGGT	
Ccl8	F: GGCCAGATAAGGCTCCAGTC	RNA-seq/RT-qPCR
	R: TGTGTGGGGTCTACACAGAGA	
Cck	F: ACCCCTCGCCTCTAATGTCT	RNA-seq/RT-qPCR
	R: CTTCCGACCACACAGCTAGG	
Ctss	F: ACATCTTTGGAGTGAGCACCA	RNA-seq/RT-qPCR
	R: CCATCTTCTGATGGCAGCGTG	
Irf7	F: TGGAGCCATGGGTATGCAAG	RNA-seq/RT-qPCR
	R: TGCTGAAGTCGAAGATGGGG	
Naip2	F: ACTCACAGATGCGCAGTGAA	RNA-seq/RT-qPCR
	R: CAGCAAAAGCACTGAACCCC	
Oas2	F: TTCCCTGGGGCTGCATAAAG	RNA-seq/RT-qPCR
	R: GCTGGGAAGGTTATGGGACC	

Pira2	F: AGAAGCCAGCAAACAAGGCT	RNA-seq/RT-qPCR
	R: GGGTCACTGTAGCCTGATGA	
Sp100	F: AAGAACTGGCGGCAAACGAT	RNA-seq/RT-qPCR
	R: TTGGCCTCAGACACCATAGC	
Tex40	F: CCCCTGGAACTCCCTAGTCT	RNA-seq/RT-qPCR
	R: GCCTAGCCATCAGGAGAGTG	
Wdfy1	F: GCAAGATAGAGGGCCACCAG	RNA-seq/RT-qPCR
	R: AAATATCCGCCTGCTGTCGT	
Zbp1	F: TGGAGTCACACAAGAGTCCC	RNA-seq/RT-qPCR
	F: TGGAGTCACACAAGAGTCCC	
Myh3	F: CTCTCTGTCACAGTCAGAGGTGT	RNA-seq/RT-qPCR
	R: TTGGCATCAAAGGGCTGGTT	
Pax2	F: GGGAAGCTACCCTACCTCCA	RNA-seq/RT-qPCR
	R: TGCTGAATCTCCAAGCCTCA	
Shisa6	F: TTATACCCCTGCCCCCAGTT	RNA-seq/RT-qPCR
	R: CTGGCTTCTCTGTGGCTCTC	
