# Supplementary material for: UHRF1 suppresses retrotransposons and cooperates with PRMT5 and PIWI proteins in male germ cells
Source: Nat Commun. 2019 Oct 17;10:4705. doi: 10.1038/s41467-019-12455-4 (PMC6797737; doi:10.1038/s41467-019-12455-4)
Supplement: Supplementary file 3 — Reporting Summary [file 41467_2019_12455_MOESM3_ESM.pdf]

nature research

Corresponding author(s): Shuiqiao Yuan

Last updated by author(s): Aug 15, 2019

Reporting Summary

Nature Research wishes to improve the reproducibility of the work that we publish. This form provides structure for consistency and transparency in reporting. For further information on Nature Research policies, see Authors & Referees and the Editorial Policy Checklist .

Statistics

For all statistical analyses, confirm that the following items are present in the figure legend, table legend, main text, or Methods section.

n/a	Confirmed	
☐	☒	The exact sample size (*n*) for each experimental group/condition, given as a discrete number and unit of measurement
☐	☒	A statement on whether measurements were taken from distinct samples or whether the same sample was measured repeatedly
☐	☒	The statistical test(s) used AND whether they are one- or two-sided<br>*Only common tests should be described solely by name; describe more complex techniques in the Methods section.*
☐	☒	A description of all covariates tested
☐	☒	A description of any assumptions or corrections, such as tests of normality and adjustment for multiple comparisons
☐	☒	A full description of the statistical parameters including central tendency (e.g. means) or other basic estimates (e.g. regression coefficient) AND variation (e.g. standard deviation) or associated estimates of uncertainty (e.g. confidence intervals)
☐	☒	For null hypothesis testing, the test statistic (e.g. *F*, *t*, *r*) with confidence intervals, effect sizes, degrees of freedom and *P* value noted<br>*Give P values as exact values whenever suitable.*
☒	☐	For Bayesian analysis, information on the choice of priors and Markov chain Monte Carlo settings
☐	☒	For hierarchical and complex designs, identification of the appropriate level for tests and full reporting of outcomes
☐	☒	Estimates of effect sizes (e.g. Cohen's *d*, Pearson's *r*), indicating how they were calculated

Our web collection on statistics for biologists contains articles on many of the points above.

Software and code

Policy information about availability of computer code

Data collection	The detailed data collection in this study were found in Figure legend and Method sections.
Data analysis	SPSS16.0 software, UCSC mm10 , Repbase (http://www.girinst.org/repbase/) were used for data analysis.

For manuscripts utilizing custom algorithms or software that are central to the research but not yet described in published literature, software must be made available to editors/reviewers. We strongly encourage code deposition in a community repository (e.g. GitHub). See the Nature Research guidelines for submitting code & software for further information.

Data

Policy information about availability of data

All manuscripts must include a data availability statement. This statement should provide the following information, where applicable:

- Accession codes, unique identifiers, or web links for publicly available datasets
- A list of figures that have associated raw data
- A description of any restrictions on data availability

All RNA sequencing data and ChIP-seq data are deposited in the NCBI SRA (Sequence Read Achieve) database with the accession number of SRP151332. All other supported data of this study are available from the corresponding author upon reasonable request.

Field-specific reporting

Please select the one below that is the best fit for your research. If you are not sure, read the appropriate sections before making your selection.

☒ Life sciences ☐ Behavioural & social sciences ☐ Ecological, evolutionary & environmental sciences

Life sciences study design

All studies must disclose on these points even when the disclosure is negative.

Sample size	The sample size of each group is more than 3, because all experiments should be done at least 3 times to confirm the result. Sample size was determined to be adequate based on the consistency of measurable differences between each group.
Data exclusions	No data were excluded.
Replication	The replicate data were reliable.
Randomization	No randomization of mice. Mice analyzed were litter mates and sex-matched whenever possible.
Blinding	Investigators were not blinded to mouse genotypes during experiments.

Reporting for specific materials, systems and methods

We require information from authors about some types of materials, experimental systems and methods used in many studies. Here, indicate whether each material, system or method listed is relevant to your study. If you are not sure if a list item applies to your research, read the appropriate section before selecting a response.

Materials & experimental systems

n/a	Involved in the study	
☐	☒	Antibodies
☐	☒	Eukaryotic cell lines
☒	☐	Palaeontology
☐	☒	Animals and other organisms
☒	☐	Human research participants
☒	☐	Clinical data

Methods

n/a	Involved in the study	
☐	☒	ChIP-seq
☒	☐	Flow cytometry
☒	☐	MRI-based neuroimaging

Antibodies

Antibodies used	Supplementary Table8 provided with manuscript contains information on all antibodies used in the study.
Validation	All antibodies were validated by the manufacturer and the detailed dilution ratio was provided in the supplementary Table 8. Antibody specificity was evaluated using the proper negative controls.

Eukaryotic cell lines

Policy information about cell lines

Cell line source(s)	HEK293T and NIH3T3 cells (Fig.6e-f, Fig.7b and Supplementary Fig.13) used in this study were obtained from Stem Cell Bank at Chinese Academy of Sciences. More information in Methods section.
Authentication	None of the lines have been authenticated.
Mycoplasma contamination	HEK293T and NIH3T3 cells used in this study were tested negative for mycoplasma.
Commonly misidentified lines (See ICLAC register)	No cell lines used are listed in the database of commonly misidentified cell lines.

Animals and other organisms

Policy information about studies involving animals; ARRIVE guidelines recommended for reporting animal research

Laboratory animals	Description of research mice used for experiments can be found in the relevant figure legends and Methods. All of mice were back crossed to C57BL/6 background, and only male mice were used for experiment.
Wild animals	This study did not involve wild animals.
Field-collected samples	This study did not involve samples collected from the field.

Ethics oversight | All animal procedures were approved by the Institutional Animal Care and Use Committee of Tongji Medical College, Huazhong University of Science and Technology in China, and the mice were housed in the specific pathogen free facility of Huazhong University of Science and Technology.

Note that full information on the approval of the study protocol must also be provided in the manuscript.

ChIP-seq

Data deposition

- [x] Confirm that both raw and final processed data have been deposited in a public database such as GEO.
- [ ] Confirm that you have deposited or provided access to graph files (e.g. BED files) for the called peaks.

Data access links<br>*May remain private before publication.*	https://www.ncbi.nlm.nih.gov/search/all/?term=SRP151332
Files in database submission	Supplementary Fig.10 and Table 1
Genome browser session<br>(e.g. UCSC)	https://www.ncbi.nlm.nih.gov/search/all/?term=SRP151332

Methodology

Replicates	Two replicates for WT and cKO samples were used.
Sequencing depth	Sequencing depth for each experiment is 2G~3G, the total number of clean reads is 26137000~33498456, uniquely mapped reads is about 10%, length of reads is 150bp and they were paired end.
Antibodies	Anti-RNA polymerase II CTD repeat YSPTSPS antibody from abCam, catalog number: ab817
Peak calling parameters	peak calling was performed by MACS2 (https://github.com/taoliu/MACS), with input used as the control. For MACS2, default parameters with broad peak option and a broad-cutoff of 0.05 (P value) were used.
Data quality	FastQC was used to detect the data quality for the raw data and clean data, including quality distribution, balancing analysis of base. 7620 peaks are at FDR 5% and above 5-fold enrichment for cKO sample, 10631 peaks are at FDR 5% and above 5-fold enrichment for WT sample.
Software	The softwares of FastQC (version v0.11.5), Trimmomatic (version 0.36), STAR (version v2.5.3a), RSeQC (version 2.6), MACS2 (version 2.1.1), Homer (version v4.10), deepTools (version 2.4.1) and ChIPseeker (version 1.5.1) were used to analyze the ChiP-Seq data in this study.
