# Supplementary material for: UHRF1 suppresses retrotransposons and cooperates with PRMT5 and PIWI proteins in male germ cells
Source: Nat Commun. 2019 Oct 17;10:4705. doi: 10.1038/s41467-019-12455-4 (PMC6797737; doi:10.1038/s41467-019-12455-4)
Supplement: Supplementary file 4 — Description of Additional Supplementary Files [file 41467_2019_12455_MOESM4_ESM.doc]

**Description of Additional Supplementary Files**

File name: Supplementary data 1

Description: CHIP-Seq revealed the occupancy alteration in RNA Pol-II from 214 piRNA clusters between *Uhrf1* cKO and WT control testes at P21.

File name: Supplementary data 2

Description: Analysis of repeat elements of retrotransposons from RNA-Seq at P12 WT control and *Uhrf1* cKO testes.

File name: Supplementary data 3

Description: Total identified genes in *Uhrf1* cKO and WT control testes at P12 by RNA-Seq.

File name: Supplementary data 4

Description: Total 213 deregulated genes in *Uhrf1* cKO testes at P12 determined by RNA-Seq.

File name: Supplementary data 5

Description: The identified gene expression in *Uhrf1* cKO compared to WT control testes at P9 by RNA-Seq.

File name: Supplementary data 6

Description: GO analyses for total identified genes and deregulated genes in *Uhrf1* cKO testes at P12.

File name: Supplementary data 7

Description: KEGG analysis of deregulated genes from RNA-Seq data.
