# Supplementary material for: UHRF1 suppresses retrotransposons and cooperates with PRMT5 and PIWI proteins in male germ cells
Source: Nat Commun. 2019 Oct 17;10:4705. doi: 10.1038/s41467-019-12455-4 (PMC6797737; doi:10.1038/s41467-019-12455-4)
Supplement: Supplementary file 2 — Peer Review [file 41467_2019_12455_MOESM2_ESM.pdf]

Reviewers' comments:

Reviewer #1 (Remarks to the Author):

Dong and colleagues report in this manuscript the consequences of the conditional knockout of UHRF1 in differentiating spermatogonia of mice.

Technically the work appears well conducted, with good evaluation of the spermatogenic development and some simple biochemistry to characterize protein-protein interactions. However, conceptually this work has major problems. The authors seem to completely ignore two extremely important pieces of information:

1) UHRF1 is essential for a maintenance of cytosine DNA methylation during DNA replication upon cell divisions. This has been known as far back as 2007 (Sharif et al., Nature 2007; Bostick et al., Science 2007), and the knowledge involving its mechanisms and effects of UHRF1 loss-of-function has been refined since then. Without UHRF1, genome-wide DNA methylation is rapidly and irreversibly lost across cell divisions.

2) The driver used for the conditional knockout, Stra8-Cre, is expressed on early undifferentiated spermatogonia, knocking out UHRF1 in differentiating spermatogonia. Differentiating spermatogonia undergo dozens of cell divisions: each undifferentiated spermatogonia upon which the Stra8-Cre-mediated deletion of UHRF1 occurs will divide to give rise to >1000 differentiated spermatogonia about to enter the leptotene stage of meiosis.

If you combine the two, you will expect a complete and irreversible loss of DNA methylation in germ cells by the time they enter meiosis.

De novo DNA methylation in the male germline happens mostly in fetal gonads until shortly after birth (E13.5 to P2). When undifferentiated spermatogonia appear at P3, DNA methylation is maintained throughout all these cell divisions by DNMT1 and UHRF1 (among others). The null hypothesis for their experimental approach of conditionally knocking out UHRF1 in undifferentiated spermatogonia should have been 1) loss of DNA methylation genome-wide due to lack of maintenance across spermatogonia cell divisions, and 2) meiotic arrest due to having a hypomethylated genome.

Failure to establish DNA methylation leading to problems at meiosis and retrotransposon reactivation has been known at least since 2004 (Bourchis & Bestor, Nature 2004), and its links with the fetal (pre-pachytene) piRNA pathway that is active during de novo methylation have also been long elucidated (data from likely more than 50 papers now support this model).

Instead the authors went to extents to build up a confusing model upon which somehow UHRF1 cooperates with PRMT5 and PIWI proteins to repress retrotransposons during meiosis. This utterly disregards more than a decade of work in the field.

I agree the interactions with PRMT5, MILI and MIWI in pachytene spermatocytes are interesting, but they are absolutely not related to any DNA methylation defects nor with the phenotype they observe: constitutive knock out of MIWI or the conditional knockout of MILI (Stra8-Cre) leads to failure to produce pachytene piRNAs and arrest of spermatogenesis at the round spermatid stage. This is not what the authors see on their Uhrf1 cKO males. Instead, what they have in hands is -with a very high degree of probability- a phenotype due to DNA methylation defects at retrotransposons just as in DNMT3L, MIWI2, and constitutive MILI knockouts (among other mutants of the fetal piRNA pathway). This does not in any conceivable way originates from any cooperation of UHRF1-PRMT5-MILI-MIWI, but rather reflects –very much expectedly—the loss of the ability to maintain DNA methylation that was established in fetal germ cells. To be scientifically accurate they must acknowledge the literature and analyze their results in light of what is known due to decades of previous work.

I suggest the authors stand by their novel and potentially interesting results of the interactions between UHRF1 and PRMT5, MILI and MIWI. They should use a Cre driver line to conditionally knocking out UHRF1 in meiosis, ideally upon the leptotene-zygotene, when DNA replication is finished and any confounding effects of DNA methylation defects due to lack of maintenance can be avoided. This will allow the authors to effectively test if UHRF1 plays a role in these cytoplasmic interactions with PRMT5/MILI/MIWI and pachytene piRNA biogenesis, and provide a starting point to test what this role might be.

Reviewer #2 (Remarks to the Author):

Dong et al examined the functional requirements of UHRF1 protein in male germ cells of mice. They describe an essential role of the protein in DNA methylation, retrotransposon repression and progression through meiotic prophase I. In addition, for the first time, the authors reports an association of UHRF1 with Piwi proteins and PRMT5. Overall, it is a novel, well-designed study that can be published as is.

Reviewer #3 (Remarks to the Author):

UHRF1 suppresses retrotransposons and cooperates with PRMT5 and PIWI proteins in male germ cells.

Dong et al.
The above work explores the role of UHRF1 in spermatogenesis and retrotransposon silencing through targeted gene knockout of UHRF1 during mouse development. The authors disrupt UHRF1 in the postnatal male germline using Stra8-Cre transgenic mice and this results in grossly abnormal testis and complete sterility of the mice. Loss of UHRF1 leads to derepression of LINE1 and IAP-type retrotransposons, including expression of LINE1 ORF1 protein. Derepression of these elements is accompanied by a decrease in H3K9me3 and DNA methylation and decreased H4R3me2 and H3R2me2. The function of UHRF1 in spermatogenesis involves its interaction with PIWI proteins and PRMT5. I think that this work represents a still largely unexplored and important area but it is unclear how UHRF1 potentially links the piRNA pathway to histone and DNA methylation. I support the publication of this work provided that the text and the model are improved in their clarity, regarding the proposed interplay of piRNAs, PRMT5 and UHRF1. I also have some minor comments.

Minor comments

1.It is unclear how piRNAs, PRMT5, UHRF1, H3K9me3 and DNA methylation are linked from reading the manuscript text or looking through the Figures. Also, the model at the end of the Figures does not help. The model could be improved by drawing on the literature as well with relevant papers cited. Is UHRF1 recruited to hemi-methylated DNA and then acts as a scaffold to link piRNAs/PIWI proteins to histone and DNA methylation through its binding to MILI/MIWI, PRMT5, SETDB1(?) And DNMTs? In the model, it is stated that UHRF1 regulates piRNA biogenesis but this is not shown.

2.Fig 1: How many experiments is this representative of to support the summary diagram of UHRF1 localization in Figure 1?

3.Fig 5b: Not clear why the actin promoter is enriched for H3K9me3 and to a higher extent than repetitive elements. Need different negative and positive controls that show low vs. high H3K9me3 in this assay.

4.Fig 5c: Again, not clear why the actin promoter is revealing lower H3K4me3 levels than repetitive elements in control cells. Since this result for control primers is not expected, other positive / negative control primers are required here.

5.Bisulfate should be “bisulfite” in figure 4 legend.

RESPONSE TO REVIEWERS

GENERAL COMMENTS FOR ALL REVIEWERS

We thank the Reviewers for their careful consideration of our manuscript (NCOMMS-18-29481) and for their helpful comments, which have allowed us to significantly improve the study. All Reviewers found our work to be interesting and novel. Reviewer #2 was very positive about the work and found that "it is a novel, well-designed study that can be published as is". Reviewer #3 was largely convinced by our functional studies of UHRF1 in spermatogenesis. However, Reviewer #1 raised an important criticism regarding the interpretation of data in light of previous functional studies of UHRF1. To address the critical point raised by Reviewer #1, we have extensively revised the Abstract, Introduction, Results and Discussion sections to accurately interpret our data in light of previous functional studies of UHRF1. This comment enabled us to significantly improve the manuscript and highlight the significance of our study. We also revised several specific points in response to Reviewer #3, which further helped us to clarify the model and important take-home messages of this study.

Our specific comments to each reviewer follow. Please note: *Reviewer comments are in italics.* **Our responses are in bold. The comments from the Reviewers have not been edited. We thank you again for your feedback and consideration.**

Reviewer #1 (Remarks to the Author):

Dong and colleagues report in this manuscript the consequences of the conditional knockout of UHRF1 in differentiating spermatogonia of mice. Technically the work appears well conducted, with good evaluation of the spermatogenic development and some simple biochemistry to characterize protein-protein interactions. However, conceptually this work has major problems. The authors seem to completely ignore two extremely important pieces of information:

1) UHRF1 is essential for a maintenance of cytosine DNA methylation during DNA replication upon cell divisions. This has been known as far back as 2007 (Sharif et al., Nature 2007; Bostick et al., Science 2007), and the knowledge involving its mechanisms and effects of UHRF1 loss-of-function has been refined since then. Without UHRF1, genome-wide DNA methylation is rapidly and irreversibly lost across cell divisions.
2) The driver used for the conditional knockout, Stra8-Cre, is expressed on early undifferentiated spermatogonia, knocking out UHRF1 in differentiating spermatogonia. Differentiating spermatogonia undergo dozens of cell divisions: each undifferentiated spermatogonia upon which the Stra8-Cre-mediated deletion of UHRF1 occurs will divide to give rise to >1000 differentiated spermatogonia about to enter the leptotene stage of meiosis.
If you combine the two, you will expect a complete and irreversible loss of DNA methylation in germ cells by the time they enter meiosis.

De novo DNA methylation in the male germline happens mostly in fetal gonads until shortly after birth (E13.5 to P2). When undifferentiated spermatogonia appear at P3, DNA methylation is maintained throughout all these cell divisions by DNMT1 and UHRF1 (among others). The null hypothesis for their experimental approach of conditionally knocking out UHRF1 in undifferentiated spermatogonia should have been 1) loss of DNA methylation genome-wide due to lack of maintenance across spermatogonia cell divisions, and 2) meiotic arrest due to having a hypomethylated genome.

Failure to establish DNA methylation leading to problems at meiosis and retrotransposon reactivation has been known at least since 2004 (Bourchis & Bestor, Nature 2004), and its links with the fetal (pre-pachytene) piRNA pathway that is active during de novo methylation have also been long elucidated (data from likely more than 50 papers now support this model). Instead the authors went to extents to build up a confusing model upon which somehow UHRF1 cooperates with PRMT5 and PIWI proteins to repress retrotransposons during meiosis. This utterly disregards more than a decade of work in the field.

I agree the interactions with PRMT5, MILI and MIWI in pachytene spermatocytes are interesting, but they are absolutely not related to any DNA methylation defects nor with the phenotype they observe: constitutive knock out of MIWI or the conditional knockout of MILI (Stra8-Cre) leads to failure to produce pachytene piRNAs and arrest of spermatogenesis at the round spermatid stage. This is not what the authors see on their Uhrf1 cKO males. Instead, what they have in hands is -with a very high degree of probability- a phenotype due to DNA methylation defects at retrotransposons just as in DNMT3L, MIWI2, and constitutive MILI knockouts (among other mutants of the fetal piRNA pathway). This does not in any conceivable way originates from any cooperation of UHRF1-PRMT5-MILI-MIWI, but rather reflects –very much expectedly—the loss of the ability to maintain DNA methylation that was established in fetal germ cells. To be scientifically accurate they must acknowledge the literature and analyze their results in light of what is known due to decades of previous work.

I suggest the authors stand by their novel and potentially interesting results of the interactions between UHRF1 and PRMT5, MILI and MIWI. They should use a Cre driver line to conditionally knocking out UHRF1 in meiosis, ideally upon the leptotene-zygotene, when DNA replication is finished and any confounding effects of DNA methylation defects due to lack of maintenance can be avoided. This will allow the authors to effectively test if UHRF1 plays a role in these cytoplasmic interactions with PRMT5/MILI/MIWI and pachytene piRNA biogenesis, and provide a starting point to test what this role might be.

We thank the Reviewer for their careful scrutiny of our work, and we appreciate that the Reviewer has raised several important points. At the same time, we apologize that several important aspects were not clear in our previous version of this study. As we summarize below, we have extensively revised the Abstract, Introduction, Results and Discussion sections for (1) accurate citation of the literature and (2) to interpret our data in light of the previous functional studies of UHRF1. All told, the Reviewer's comment enabled us to improve the manuscript and highlight the siginificance of our study.

Given that UHRF1 is required for the maintenance of DNA methylation during DNA replication, the *Stra8-Cre*-induced deletion of *Uhrf1* in differentiating spermarmatogonia and subsequent proliferation could result in a near-complete depletion of DNA methylation in meiotic spermatocytes. While our methylation analyses demonstrated a depletion in DNA methylation, it was neither complete nor would it be accurate to describe it as near-complete; instead, we observed varied degrees of demethylation at genomic loci. This suggests that the loss-of-function of UHRF1 took place just prior to the entry into meiosis (~1 or 2 divisions prior to the entry) and that loss of DNA methylation in the *Uhrf1* conditional knockout (cKO) cannot be explained solely by defects in DNA methylation maintenance (for additional supporting rationale and evidence, please review the following sections). In line with these resutls, the glocal levels of global 5-methylcytosine (5mC) are comparable between the differentiated spermatogonia of the *Uhrf1* cKO and control (See the Fig. 4e and Supplementary Fig. 7c-d); however, the levels of 5mC were significantly reduced in the *Uhrf1* cKO upon entry into meiotic prophase I (Fig. 4d). Additionally, the new RNA-seq data at P9 testes, a time point when first-wave germ cells transition into meiosis, revealed that the global transcriptome was not changed in P9 *Uhrf1* cKO testes (See the new Supplemental Fig. 13b-c). These results suggest that our *Stra8-Cre* mouse model enabled us to determine a meiotic function for UHRF1 rather than reveal the consequences of loss of DNA methylation in progenitor cells. In support of this possibility, a previous study demonstrated that the conditional deletion of *Mili*, also via *Stra8-Cre*, resulted in the depletion of MILI in meiotic spermatocytes, while localization of MILI did not change in the mutant spermatogonia (Di Giacomo et al., *Mol Cell* 2013). That study unveiled a function for MILI in the post-transcriptinal silencing of LINE1 in meiosis. Together, these results suggest that *Stra8-Cre* is an appropriate option to the test functions of targeted proteins in meiosis.

We appreciate the Revierer's suggestion to use an anlternative Cre line with expression specific to meiotic prophase I. However, we decided not to pursue this option for this revision because of the current technical challenges that make it difficult to achieve meiosis-specific loss-of-function. There is no well-established Cre line for this purpose. Although there are some Cre lines that can induce expression specific to meiosis, mRNA tends to be very stable during meiotic prophase; that, in turn, tends to make phenotypes arising from meiosis-specific Cre expression appear in the later postmeiotic spermatid stage of spermatogenesis. All

together and at present, *Stra8*-Cre is the best-suited Cre line to dissect the roles of UHRF1 in meiosis.

Of note, the meiotic phenotype of *Uhrf1* cKO differs from those of mice with defects in *de novo* DNA methylation. DNMT3L, MILI, and MIWI2 are all essential in the *de novo* establishment of DNA methylation during fetal germ cell development (Bourc'his et al., *Nature* 2004; Carmell et al., *Dev Cell* 2007; Kuramochi-Miyagawa et al., *Genes Dev* 2008), and the constitutive knockout of *Dnmt3L, Mili*, or *Miwi2* resutls in the abolishment of DNA methylation in male germ cells, leading to meitotic arrest in the leptotene/zygotene stages, prior to completion of autosomal synapsis (Bourc'his et al., *Nature* 2004; Carmell et al., *Dev Cell* 2007; Kuramochi-Miyagawa, et al., *Development* 2004). However, autosomal synapsis completed in the *Uhrf1* cKO spermatocytes, and mutant spermatocytes were arrested at the pachytene stage (Fig. 3). With this mind, the phenotype of *Uhrf1* cKO spermatocytes cannot be explained by solely the defective maintenance of DNA methylation. Since meiotic spermatocytes already completed premeiotic DNA replication, we suspect that UHRF1 is involved in mechanisms above and beyond the maintenance of DNA methylation during meiotic prophase I.

So, to elucidate additional mechanisms regulated by UHRF1 during meiosis, we investigated H3K9 methylation, the piRNA pathway, and histone arginine methylation. We added this rationale and description to the Results section of the manuscript. We believe these new details inform a compelling rationale to study H3K9 methylation, the piRNA pathway, and histone arginine methylation in our model system.

We have unpublished data that further support the rationale. We have already generated *Vasa*-Cre (expressed on the pro-spermatogonia at E15.5)-induced *Uhrf1* cKO mouse model, and found that spermatogenesis is arrested in the transition from the leptotene-to-zygotene stages of meiosis in the *Vasa*-Cre cKO testes. The developmental phenotype of *Vasa*-Cre:*Uhrf1* cKO mice is similar to that observed in *Miwi2* and *Dnmt3L* global knockout males from previous reports (Bourc'his et al., *Nature* 2004; Carmell et al., *Dev Cell* 2007), confirming that *Stra8*-Cre:*Uhrf1* cKO mice (current study) possess a distinct phenotype from the mice in which global DNA methylation was abolished. However, these results are outside the scope of the present study; we are planning to publish them later in a separate study.

Reviewer #2 (Remarks to the Author):

Dong et al examined the functional requirements of UHRF1 protein in male germ cells of mice. They describe an essential role of the protein in DNA methylation, retrotransposon repression and progression through meiotic prophase I. In addition, for the first time, the authors reports an association of UHRF1 with Piwi proteins and PRMT5. Overall, it is a novel, well-designed study that can be published as is.

Thank you so much for your appreciation of the significance of our work. It took 3.5 years for us to get here. We cannot wait to let people know this novel finding.

Reviewer #3 (Remarks to the Author):

UHRF1 suppresses retrotransposons and cooperates with PRMT5 and PIWI proteins in male germ cells. Dong et al.
The above work explores the role of UHRF1 in spermatogenesis and retrotransposon silencing through targeted gene knockout of UHRF1 during mouse development. The authors disrupt UHRF1 in the postnatal male germline using Stra8-Cre transgenic mice and this results in grossly abnormal testis and complete sterility of the mice. Loss of UHRF1 leads to derepression of LINE1 and IAP-type retrotransposons, including expression of LINE1 ORF1 protein. Derepression of these elements is accompanied by a decrease in H3K9me3 and DNA methylation and decreased H4R3me2 and H3R2me2. The function of UHRF1 in spermatogenesis involves its interaction with PIWI proteins and PRMT5. I think that this work represents a still largely unexplored and important area but it is unclear how UHRF1 potentially links the piRNA pathway to histone and DNA methylation. I support the publication of this work provided that the text and the model are improved in their clarity, regarding the proposed interplay of piRNAs, PRMT5 and UHRF1. I also have some minor comments.

Thank you very much for this thoughtful evaluation of our work. We appreciate this reviewer for suggesting to improve the text and model of our manuscript. Following the comments, we revised the text and re-drew the working model to demonstrate the potential functions of UHRF1 linking repressive epigenetic pathways (DNA methylation, histone modification and PIWI proteins) crosstalk to repress retrotransposons in spermatocytes. We propose that UHRF1 serves as a mediator to coordinate with PRMT5-DNMT1-H3K9MTs complex in the nucleus and PRMT5/PIWI proteins in the cytoplasm to repress TEs transcriptionally and post-transcriptionally (see the updated Fig.8).

Minor comments
1.It is unclear how piRNAs, PRMT5, UHRF1, H3K9me3 and DNA methylation are linked from reading the manuscript text or looking through the Figures. Also, the model at the end of the Figures does not help. The model could be improved by drawing on the literature as well with relevant papers cited. Is UHRF1 recruited to hemi-methylated DNA and then acts as a scaffold to link piRNAs/PIWI proteins to histone and DNA methylation through its binding to MILI/MIWI, PRMT5, SETDB1(?) And DNMTs? In the model, it is stated that UHRF1 regulates piRNA biogenesis but this is not shown.

Thank you for pointing out this important aspect. We re-drew the working model based on our findings and the literature following the suggestions. Since both UHRF1 and PRMT5 are expressed in the nucleus and cytoplasm of spermatocytes (dynamic expression in different types of spermatocytes), we depicted the model of the nucleus and cytoplasm separately. Combined with the literature reported, we speculate that UHRF1-DNMT1-PRMT5 coordinates with H3K9MTs (H3K9 methyltransferases) multimeric complex to repress TEs transcriptionally in the nucleus of spermatocytes. The SRA domain of UHRF1 binds to hemi-methylated DNA, and then UHRF1 recruits DNMT1 to un-methylated DNA sites to maintain the methylation of TEs promoters (Arita et al. Nature 2008; Avvakumov et al. Nature 2008; Liu et al., Nat Commun 2013; Berkyurek et al. JBC 2014). The TTD domain of UHRF1 binds to methylated H3K9, which may stimulate H3K9MT (SUV39H1, G9a, SETDB1) to methylate H3K9 and then reinforce the REs silencing (Fritsch et al. Mol Cell 2010; Rothbart et al. Nat Struct Mol Biol 2012; Bulut-Karslioglu et al. Mol Cell 2014; Karimi et al. Cell Stem Cell. 2011). UHRF1 can interact with PRMT5 to modify the histone H4R3 (H3K4me2s, repressive histone mark) and further repress the TEs. This nuclear macromolecular complex is proposed to be involved in TEs silencing. In the cytoplasm, UHRF1 forms a complex with PRMT5 and PIWI proteins (MILI/MIWI) to facilitate the slicer activity of PIWI protein (Di Giacomo et al. Mol Cell 2013; Reuter et al. Nature 2011; Nishida et al. EMBO J 2009; Vagin et al. Genes Dev 2009), which may directly cleave transposon messenger RNAs and contribute to the repression of TEs at the post-transcriptional level.

Disruption of piRNA biogenesis might be the secondary effect caused by PIWI proteins dysfunction. We thus decided to remove piRNA biogenesis statements in the updated model (new Fig.8) and replaced them with a discussion of UHRF1-PRMT5-PIWI, wherein complexes directly cleave transposon messenger RNAs to regulate REs silencing.

We added the detailed description in the Figure legend of new Fig.8 to clarify our main finding. We hope that the new schematic model will clarify this novel finding.

2.Fig 1: How many experiments is this representative of to support the summary diagram of UHRF1 localization in Figure 1?

We performed five experiments of immuno-staining using UHRF1 and γ-H2A.X antibodies in five independent wild-type testis sections. We now added the experiment times in the Legend of Fig.1.

3.Fig 5b: Not clear why the actin promoter is enriched for H3K9me3 and to a higher extent than repetitive elements. Need different negative and positive controls that show low vs. high H3K9me3 in this assay.

We repeated the CHIP-qPCR assays following the literature reports and protocols. At this time, we used the H19 ICR (an imprinting gene control region) as a negative control, which has been

reported to not bind to the H3K9me3 (Delaval et al. EMBO J 2007). Following this reviewer's suggestion, we also added RPL-30 as a positive control, because the RPL-30 gene is actively transcribed in all cell types and its promoter is highly enriched for histone modifications. New Fig.5b was updated in the revised manuscript.

4.Fig 5c: Again, not clear why the actin promoter is revealing lower H3K4me3 levels than repetitive elements in control cells. Since this result for control primers is not expected, other positive / negative control primers are required here.

We added both positive control (RPL-30) and negative control (H19 ICR) in new CHIP-qPCR assays. All primers are listed in Supplementary Table 8.

5.Bisulfate should be "bisulfite" in figure 4 legend.

Changed as suggested.

References:

Bourc'his D, Bestor TH. Meiotic catastrophe and retrotransposon reactivation in male germ cells lacking
Dnmt3L. Nature, 2004, 431: 96-99.

Carmell MA, Girard A, van de Kant HJ, Bourc'his D, Bestor TH, de Rooij DG, Hannon GJ. MIWI2 is essential for spermatogenesis and repression of transposons in the mouse male germline. Dev Cell, 2007, 12: 503-514.

Kuramochi-Miyagawa S, Kimura T, Ijiri TW, Isobe T, Asada N, Fujita Y, Ikawa M, Iwai N, Okabe M, Deng W, Lin H, Matsuda Y, Nakano T. Mili, a mammalian member of piwi family gene, is essential for spermatogenesis. Development, 2004, 131: 839-849.

Di Giacomo M, Comazzetto S, Saini H, De Fazio S, Carrieri C, Morgan M, Vasiliauskaite L, Benes V, Enright AJ, O'Carroll D. Multiple epigenetic mechanisms and the piRNA pathway enforce LINE1 silencing during adult spermatogenesis. Mol Cell. 2013, 50(4):601-8.

Arita K, Ariyoshi M, Tochio H, Nakamura Y, Shirakawa M. Recognition of hemi-methylated DNA by the SRA protein UHRF1 by a base-flipping mechanism. Nature, 2008, 455: 818-821.

Avvakumov GV, et al. Structural basis for recognition of hemi-methylated DNA by the SRA domain of human UHRF1. Nature, 2008, 455: 822-825.

Liu, X., Gao, Q., Li, P., Zhao, Q., Zhang, J., Li, J., Koseki H, Wong, J. UHRF1 targets DNMT1 for DNA methylation through cooperative binding of hemi-methylated DNA and methylated H3K9. Nat Commun, 2013, 4: 1563.

Berkyurek AC, et al. The DNA methyltransferase Dnmt1 directly interacts with the SET and RING finger associated (SRA) domain of the multifunctional protein Uhrf1 to facilitate accession of the catalytic center to hemi-methylated DNA. J Biol Chem, 2014, 289: 379-386.

Fritsch L, Robin P, Mathieu JR, Souidi M, Hinaux H, Rougeulle C, Harel-Bellan A, Ameyar-Zazoua M, Ait-Si-Ali S. A subset of the histone H3 lysine 9 methyltransferases Suv39h1, G9a, GLP, and SETDB1 participate in a multimeric complex. Mol Cell. 2010, 37(1):46-56.

Rothbart SB, et al. Association of UHRF1 with methylated H3K9 directs the maintenance of DNA methylation. Nat Struct Mol Biol, 2012, 19:1155-1160.

Bulut-Karslioglu A, De La Rosa-Velázquez IA, Ramirez F, Barenboim M, Onishi-Seebacher M, Arand J, Galán C, Winter GE, Engist B, Gerle B, O'Sullivan RJ, Martens JH, Walter J, Manke T, Lachner M, Jenuwein T.
Suv39hdependent H3K9me3 marks intact retrotransposons and silences LINE elements in mouse em bryonic stem cells. Mol Cell. 2014, 55(2):277-90.

Karimi MM, Goyal P, Maksakova IA, Bilenky M, Leung D, Tang JX, Shinkai Y, Mager DL, Jones S, Hirst M, Lorincz MC.
DNA methylation and SETDB1/H3K9me3 regulate predominantly distinct sets of genes, retroelements, and chimeric transcripts in mESCs. Cell Stem Cell. 2011, 8(6):676-87.
Reuter M., Berninger P., Chuma S., Shah H., Hosokawa M., Funaya C., Antony C, Sachidanandam R, Pillai, R. S. Miwi catalysis is required for piRNA amplification-independent LINE1 transposon silencing. Nature, 2011, 480(7376), 264-267.

Nishida, K. M., Okada, T. N., Kawamura, T., Mituyama, T., Kawamura, Y., Inagaki, Siomi, M. C. Functional involvement of Tudor and dPRMT5 in the piRNA processing pathway in Drosophila germlines. EMBO J, 2009, 28(24), 3820-3831.

Vagin, V. V., Wohlschlegel, J., Qu, J., Jonsson, Z., Huang, X., Chuma, S., Aravin, A. A. Proteomic analysis of murine Piwi proteins reveals a role for arginine methylation in specifying interaction with Tudor family members. Genes Dev, 2009, 23(15), 1749-1762.

Delaval K, Govin J, Cerqueira F, Rousseaux S, Khochbin S, Feil R. Differential histone modifications mark mouse imprinting control regions during spermatogenesis. EMBO J, 2007, 26(3):720-9.

Reviewers' comments:

Reviewer #3 (Remarks to the Author):

The authors have endeavoured to answer all my concerns and this manuscript is now greatly improved and of broad interest. I still have a few minor comments that need addressing:

1. The authors still need to check their qPCR controls performed in the ChIP experiments (Figure 5). It sounds like RPL-30 is an actively transcribed gene so should be enriched for active promoter histone marks like H3K4me3 but depleted of silent histone marks like H3K9me3 compared to transposons– this is not what we appear to see on the plots? Positive control PCRs for the H3K9me3 ChIP could be any locus known to be enriched in H3K9me3. Examples are Mest or the imprinted Nnat locus. A negative control for the H3K9me3 ChIP could be any housekeeping gene promoter like Gapdh. Likewise, for the H3K4me3 ChIP, a positive control could be any active housekeeping gene promoter and a negative control could be any inactive gene, possibly Mest or Nnat.

2. Line 309: "These results indicate that global DNA methylation status was comparable between Uhrf1 cKO and control differentiating spermatogonia, but was significantly affected in Uhrf1 cKO spermatocytes". I think this is a bit misleading as it is not clear exactly when Uhfr1 knockout takes place and DNA methylation maintenance is affected as presumably the latter is gradually more affected with increasing time. Related to this, in the discussion, it needs to be clear that the meiotic defects and resulting sterility due to retrotransposon reactivation could be due to a combination of affects ie partly due to loss of DNA methylation, partly due to a decrease in arginine methylation and potentially also due to effects on the piRNA pathway.

3. Line 332: This reviewer thinks that it would be worth making this paragraph clearer and shorter.

4. Abstract: the sentence "Here, we show that UHRF1 is responsible for retrotransposon silencing and cooperates with the repressive epigenetic pathways in postnatal male germ cells" should be ""Here, we show that UHRF1 is responsible for retrotransposon silencing and cooperates with repressive epigenetic pathways in postnatal male germ cells". Other minor English corrections are needed throughout.

Reviewer #4 (Remarks to the Author):

In this paper, the authors demonstrate that conditional loss of UHRF1 in postnatal germ cells causes DNA hypomethylation, upregulation of retrotransposon, and the activation of DNA damage response, leading to complete male sterility. However, it is difficult to distinguish between the possibility that UHRF1 regulates retrotransposons at the piRNA biogenesis and the maintenance abnormality of DNA methylation, using Stra8-Cre. Although the authors substantially improved the manuscript according to the reviewer's comments, the experimental results are not enough to draw firm conclusions. While the data showing the interaction among UHRF1, PRMT5, TDRKH and MILI is potentially interesting, there is not enough data for the importance of this interaction.

Specific comments;
Fig. 5d-f; The quality of the western blotting is very poor. In Fig. 5d, PRMT5 was detected without UHRF1. Why?
Fig. 5d-f; The authors should indicate the specificity of the immunoprecipitation experiment. Silver staining data should be shown.
Fig. 5g and 7d; What is the transcriptional level of piRNAs in UHRF1-cKO? It is likely that a diffused pattern in the cytoplasm of uhrf1-cKO spermatocytes is affected by loss of piRNA transcription rather

than that of piRNA biogenesis. The authors should examine the level of piRNA transcription from piRNA clusters by Pol-II ChIP-seq or other experimental settings.
Fig. 8; The model should be removed. While the authors showed UHRF1 is required for TE silencing, they did not examine whether "the interaction" between UHRF1 and PRMT5 is critical for the epigenetic regulation in the nucleus and the posttranscriptional regulation in the cytoplasm.

POINT-BY-POINT RESPONSE TO REVIEWERS

GENERAL COMMENTS FOR EDITOR AND ALL REVIEWERS

We thank the Editor and Reviewers for their careful consideration of our revised manuscript (NCOMMS-18-29481A) and for their helpful comments, which have allowed us to significantly improve the study again. At this round review process, Reviewers #3 was satisfied with our revised work and acknowledged this study is now greatly improved and of broad interest. Newly recruited Reviewer #4 acknowledged the manuscript has been substantially improved after the previous round of revision. However, he/she raised an important criticism regarding the interpretation of data in light of the function of UHRF1 in retrotransposon regulation at the piRNA biogenesis and DNA methylation maintenance. To address the critical point raised by Reviewer #4, we have revised the *Introduction*, *Results* and *Discussion* sections again to clearly interpret our data to avoid the confounding effects of DNA methylation by UHRF1 mediation in spermatogenesis. This comment enabled us to significantly improve the manuscript and highlight the significance of our study. We also revised several specific comments in response to Reviewer #3 and #4, which further helped us to improve this study. In addition, following the suggestions from the Editor and Reviewer #4, we removed the model (Fig.8) from the revised manuscript to focus on solid data.

Our specific comments to each reviewer follow. Please note: *Reviewer comments are in italics.* **Our responses are in bold. The comments from the Reviewers have not been edited. We thank you again for your feedback and consideration.**

Reviewer #3 (Remarks to the Author):
The authors have endeavoured to answer all my concerns and this manuscript is now greatly improved and of broad interest. I still have a few minor comments that need addressing:

Thank you so much for evaluation of our work again. We appreciate your suggestions to further improve our study.

1. The authors still need to check their qPCR controls performed in the ChIP experiments (Figure 5). It sounds like RPL-30 is an actively transcribed gene so should be enriched for active promoter histone marks like H3K4me3 but depleted of silent histone marks like H3K9me3 compared to transposons– this is not what we appear to see on the plots? Positive control PCRs for the H3K9me3 ChIP could be any locus known to be enriched in H3K9me3. Examples are Mest or the imprinted Nnat locus. A negative control for the H3K9me3 ChIP could be any housekeeping gene promoter like Gapdh. Likewise, for the

H3K4me3 ChIP, a positive control could be any active housekeeping gene promoter and a negative control could be any inactive gene, possibly Mest or Nnat.

We performed the ChIP-qPCR again and changed the positive and negative controls for H3K9me3 and H3K4me3 CHIP according to the reviewer's suggestions. All primers are listed in Supplementary Table 9.

2. Line 309: "These results indicate that global DNA methylation status was comparable between Uhrf1 cKO and control differentiating spermatogonia, but was significantly affected in Uhrf1 cKO spermatocytes". I think this is a bit misleading as it is not clear exactly when Uhfr1 knockout takes place and DNA methylation maintenance is affected as presumably the latter is gradually more affected with increasing time. Related to this, in the discussion, it needs to be clear that the meiotic defects and resulting sterility due to retrotransposon reactivation could be due to a combination of affects ie partly due to loss of DNA methylation, partly due to a decrease in arginine methylation and potentially also due to effects on the piRNA pathway.

We changed this sentence to "These results indicate that global DNA methylation status (5mC) is comparable between *Uhrf1* cKO and control differentiating spermatogonia, but show decreased levels in *Uhrf1* cKO spermatocytes", which describes the phenomena without over-interpretation. In the discussion section, we also discussed the possibility of meiotic defects in *Uhrf1* cKO mice due to a combination of effects from DNA methylation, histone arginine methylation and the piRNA pathway.

3. Line 332: This reviewer thinks that it would be worth making this paragraph clearer and shorter.
We rewrote this paragraph to make it shorter and more precise.

4. Abstract: the sentence "Here, we show that UHRF1 is responsible for retrotransposon silencing and cooperates with the repressive epigenetic pathways in postnatal male germ cells" should be ""Here, we show that UHRF1 is responsible for retrotransposon silencing and cooperates with repressive epigenetic pathways in postnatal male germ cells". Other minor English corrections are needed throughout.
Done as suggested. In this revision, a native speaker went through the entire manuscript and corrected minor errors in English.

Reviewer #4 (Remarks to the Author)*:*
In this paper, the authors demonstrate that conditional loss of UHRF1 in postnatal germ cells causes DNA hypomethylation, upregulation of retrotransposon, and the activation of DNA damage response, leading to complete male sterility. However, it is difficult to distinguish between the possibility that UHRF1 regulates retrotransposons at the piRNA biogenesis and the maintenance abnormality of DNA methylation, using Stra8-Cre. Although the authors

substantially improved the manuscript according to the reviewer's comments, the experimental results are not enough to draw firm conclusions. While the data showing the interaction among UHRF1, PRMT5, TDRKH and MILI is potentially interesting, there is not enough data for the importance of this interaction.

We thank this Reviewer for his/her careful scrutiny of our work, and for finding that the manuscript was substantially improved in the previous round of revision. We also appreciate this Reviewer for raising several important suggestions to draw firm conclusions. In this revised manuscript, to avoid the confounding effects of DNA methylation by UHRF1 mediation in spermatogenesis, we extensively revised the *Introduction*, *Results* and *Discussion* sections to clearly interpret our data (see the text with yellow highlighting).

As pointed out by this reviewer, we agree that it is important to distinguish between the possibility that UHRF1 regulates retrotransposons at piRNA biogenesis and the possibility that piRNA transcription was abolished upon *Uhrf1* deletion. To further confirm the original conclusion of defective piRNA biogenesis in *Uhrf1* cKO mice, we performed the suggested experiment and excluded the possibility that piRNA transcription was abolished upon *Uhrf1* deletion. Specifically, we performed RNA Pol-II ChIP-seq to examine the level of piRNA transcription from piRNA clusters as this reviewer suggested. Interestingly, only ~10% of piRNA clusters showed deregulated Pol-II occupancy in *Uhrf1* cKO mice, which means the level of piRNA transcription from ~90% of piRNA clusters was not affected. Combined with the data of UHRF1 interacting with PIWI proteins (TDRKH, MIWI and MILI), we speculate that UHRF1 regulates piRNA biogenesis to repress retrotransposons by cooperating with PIWI proteins. We also understand a need to elucidate the underlying mechanism by which UHRF1 regulates piRNA biogenesis in future studies as a separate project. We hope that additional data described below provided firm conclusions.

Specific comments;
Fig. 5d-f; The quality of the western blotting is very poor. In Fig. 5d, PRMT5 was detected without UHRF1. Why?

We performed the western blotting assay multiple times and chose the best representative data. Please see the new Fig.5d-f.
In this study, we specifically knocked out UHRF1 in germ cells. However, the UHRF1 could interact with PRMT5 in somatic cells of testes as well. We suspect this is the reason why PRMT5 was detectable at a reduced level in *Uhrf1* cKO testes compared to the control testes.

Fig. 5d-f; The authors should indicate the specificity of the immunoprecipitation experiment. Silver staining data should be shown.

We performed silver staining and showed the new data in the Supplementary Fig.9c-e.

Fig. 5g and 7d; What is the transcriptional level of piRNAs in UHRF1-cKO? It is likely that a diffused pattern in the cytoplasm of uhrf1-cKO spermatocytes is affected by loss of piRNA transcription rather than that of piRNA biogenesis. The authors should examine the level of piRNA transcription from piRNA clusters by Pol-II ChIP-seq or other experimental settings.

Thank you for pointing out this important aspect. To examine the piRNA transcriptional levels from piRNA clusters (214 reported clusters in *Mol cell*, Li et al, 2013), we performed the RNA Pol-II ChIP-seq as suggested. After carefully analyzing the ChIP-seq data, we only found that a total of 22 piRNA precursors displayed dysregulated RNA Pol-II occupancy in *Uhrf1* cKO testes compared with those of WT testes, including 17 piRNA precursors showing up-regulated enrichment and 5 piRNA precursors showing down-regulated enrichment (Supplementary Fig.10a-d and Table 1). These Pol-II ChIP-seq data suggest that the level of piRNA transcription from the majority of piRNA clusters was not significantly changed upon *Uhrf1* deletion in testes.

Fig. 8; The model should be removed. While the authors showed UHRF1 is required for TE silencing, they did not examine whether "the interaction" between UHRF1 and PRMT5 is critical for the epigenetic regulation in the nucleus and the posttranscriptional regulation in the cytoplasm.

We agreed with this point. The model (Fig.8) has been removed from the revised manuscript.

REVIEWERS' COMMENTS:

Reviewer #3 (Remarks to the Author):

The authors have now sufficiently addressed all of my outstanding concerns and this manuscript is now suitable for publication.

Reviewer #4 (Remarks to the Author):

The revised manuscript is much improved over the original version and the authors addressed all comments. Thus, this reviewer recommends publication of the work in Nature Communications.
